# The demographic causes of population change vary across four decades in a long‐lived shorebird

**DOI:** 10.1002/ecy.3615

**Published:** 2022-03-03

**Authors:** Andrew M. Allen, Eelke Jongejans, Martijn van de Pol, Bruno J. Ens, Magali Frauendorf, Martijn van der Sluijs, Hans de Kroon

**Affiliations:** ^1^ Department of Animal Ecology Netherlands Institute for Ecology (NIOO‐KNAW) Wageningen The Netherlands; ^2^ Department of Animal Ecology and Physiology Radboud University Nijmegen The Netherlands; ^3^ Centre for Avian Population Studies Wageningen The Netherlands; ^4^ College of Science and Engineering James Cook University Townsville Queensland Australia; ^5^ Sovon Dutch Centre for Field Ornithology Sovon‐Texel Texel The Netherlands; ^6^ Department of Experimental Plant Ecology Radboud University Nijmegen The Netherlands

**Keywords:** demographic mechanisms, individual heterogeneity, integral projection models, population decline

## Abstract

Understanding which factors cause populations to decline begins with identifying which parts of the life cycle, and which vital rates, have changed over time. However, in a world where humans are altering the environment both rapidly and in different ways, the demographic causes of decline likely vary over time. Identifying temporal variation in demographic causes of decline is crucial to assure that conservation actions target current and not past threats. However, this has rarely been studied as it requires long time series. Here we investigate how the demography of a long‐lived shorebird (the Eurasian Oystercatcher *Haematopus ostralegus*) has changed in the past four decades, resulting in a shift from stable dynamics to strong declines (−9% per year), and recently back to a modest decline. Since individuals of this species are likely to respond differently to environmental change, we captured individual heterogeneity through three state variables: age, breeding status, and lay date (using integral projection models). Timing of egg‐laying explained significant levels of variation in reproduction, with a parabolic relationship of maximal productivity near the average lay date. Reproduction explained most variation in population growth rates, largely due to poor nest success and hatchling survival. However, the demographic causes of decline have also been in flux over the last three decades: hatchling survival was low in the 2000s but improved in the 2010s, while adult survival declined in the 2000s and remains low today. Overall, the joint action of several key demographic variables explain the decline of the oystercatcher, and improvements in a single vital rate cannot halt the decline. Conservations actions will thus need to address threats occurring at different stages of the oystercatcher's life cycle. The dynamic nature of the threat landscape is further supported by the finding that the average individual no longer has the highest performance in the population, and emphasizes how individual heterogeneity in vital rates can play an important role in modulating population growth rates. Our results indicate that understanding population decline in the current era requires disentangling demographic mechanisms, individual variability, and their changes over time.

## INTRODUCTION

Many wildlife populations across the globe have been declining dramatically in recent decades (Barnosky et al., 2011). Stopping these declines requires targeting the factors that drive these changes (Johnson et al., 2010; Newton, 2004). A first step in this identification process is to understand the demographic causes of decline (Caswell, 2000; Johnson et al., 2010), as showing where in the life‐cycle problems occur is important for identifying the environmental drivers of such change (the ultimate targets of conservation action). However, the way in which humans impact the environment is vast but never constant. Examples include the increasing impacts of climate change (Buitenwerf et al., 2015), fertilizer use and nutrient influx to ecosystems (Lu & Tian, 2017), and use of pesticides (Ydenberg et al., 2017). Furthermore, the recent recovery of avian and mammalian predators may have implications, both direct and indirect, for the demography of their prey (Paine et al., 1990; Ydenberg et al., 2017). The omnipresence of rapid anthropogenic change in many potential environmental drivers mean that the demographic causes of population decline will likely vary over time in response to this changing risk landscape.

Understanding the extent to which demographic causes of population change can vary over short ecological timeframes is challenging, not in the least because it requires time series that span decades to detect such changes. The few studies available have focused on species with fluctuating population sizes and show how the demographic mechanisms of population growth and decline vary over time (Coulson et al., 2001; Oli & Armitage, 2004). For example, in yellow‐bellied marmot (*Marmota flaviventris*), fertility was an important vital rate, but the importance of age of first reproduction and juvenile survival varied in the growth and decline phases of the population (Oli & Armitage, 2004). However, little is known about how the demographic mechanisms of species with persistent population declines may vary. Understanding the demographic mechanisms and key demographic variables of population decline forms a vital step in identifying previous and current threats to a population (Selwood et al., 2015), and how these can be effectively mitigated for population recovery (Caruso et al., 2020; Oli & Armitage, 2004).

Another challenge in understanding the demographic causes of population change is that a population consists of individuals of different states, i.e., the condition or quality of an individual, meaning that survival and reproduction is not homogenous among individuals (Coulson et al., 2001; McNamara & Houston, 1996). For instance, young, inexperienced animals often have lower survival rates than older ones (Coulson et al., 2001; Oli & Armitage, 2004). The importance of understanding individual heterogeneity in vital rates has recently been highlighted because of the consequences of changes in population structure (Coulson et al., 2001; Vindenes & Langangen, 2015) and how individuals may respond differently to the environment, such as extreme climatic events (Jenouvrier et al., 2015). Given the way that the environment is changing due to human actions and climate change, we need to understand how individuals, and their vital rates, are responding to this change. Individual heterogeneity in vital rates may be estimated using either discrete or continuous state variables like sex (Coulson et al., 2001), age (Caswell, 2001), size (Easterling et al., 2000), or body mass (Ozgul et al., 2010). Methods in population modeling have been continuously developing to include the complex life cycles of wildlife, including vital rate changes over the life span of an individual and variation among individuals (Plard et al., 2019; Rees et al., 2014). These developments are providing new opportunities to identify which vital rates of a threatened population contribute to its decline.

The Eurasian Oystercatcher (*Haematopus ostralegus*) is an example of a species with a complex life cycle that has declined significantly in recent decades (Ens & Underhill, 2014; van de Pol et al., 2014). Oystercatchers are a long‐lived species with delayed maturity and the reproductive cycle consists of different phases during which threats may vary (Ens et al., 2014; van de Pol et al., 2010b). As a long‐lived species, oystercatchers belong to the slow end of the fast‐slow life history continuum, i.e., the life history strategy is to prioritize survival over reproduction (Sæther, 1988; Stearns, 1989), and therefore population growth tends to be most sensitive to changes in adult survival (Sæther & Bakke, 2000; Van De Pol et al., 2006). General conservation advice would thus suggest that management efforts should be focused on environmental factors that improve survival to yield improvements in population growth rates (Caswell, 2000; Manlik et al., 2018; van de Kerk et al., 2013). However, the environmental canalization hypothesis postulates that the most important vital rates tend to have least temporal variation (Gaillard & Yoccoz, 2003; Manlik et al., 2016), which was also found in the oystercatcher whereby variation in adult survival was less than in other vital rates like juvenile survival (van de Pol et al., 2010c). Studies have also found less individual heterogeneity in the vital rates that have the largest effect on population growth (Jenouvrier et al., 2015; Péron et al., 2016). Studies so far have suggested that the oystercatcher decline is associated with both low reproduction and low survival (Allen et al., 2019a; Roodbergen et al., 2012), while population projections show that both survival and reproduction will be impacted by climate change albeit in opposing ways (van de Pol et al., 2010c). An outstanding question is which of these vital rates have been most important for explaining population change and whether this importance has changed over time.

We investigate how the vital rates of the Eurasian oystercatcher have changed over four decades, from a period when the population was relatively stable (1980s) through to a decline that persists to this day. We use integral projection models (IPMs; Easterling et al., 2000; Ellner & Rees, 2006), to encapsulate both individual heterogeneity in vital rates, and to describe detailed life histories of survival and reproduction. Our analysis enables an accurate assessment of how vital rates have changed from the 1980s through to present day, and how these may vary among individuals; knowledge needed to identify the demographic causes of population change and the conservation actions that target current drivers of decline.

## STUDY SPECIES AND AREA

### Oystercatcher life cycle

The Eurasian Oystercatcher is a medium‐sized long‐lived shorebird with annual survival rates of ~90% that vary among seasons and areas (Allen et al., 2019a). Individuals form long‐term pair bonds, show high breeding site fidelity and both parents defend the breeding territory where parental care is equally shared (van de Pol et al., 2014). The breeding season is from late April to late July. Clutch size varies between two and four eggs, which are incubated ~27 d. Hatched chicks average another 28 days to fledging but these rates may vary from 21 to 50 days (Kersten & Brenninkmeijer, 1995). Oystercatchers reach sexual maturity at age three although many individuals delay recruitment into the breeding population for several more years, especially if queueing for high‐quality territories (Ens et al., 1995; van de Pol et al., 2006). Once becoming a breeder, individuals tend to have high probabilities of remaining a breeder, although breeding years may be skipped following the loss of the breeding territory or partner, e.g., following divorce or widowing (Ens et al., 1993).

### Study area

Oystercatchers have been intensively monitored on the island of Schiermonnikoog (53°29′ N, 06°40′ E) from 1983 to present. Schiermonnikoog is part of the Dutch Wadden Sea, which is an internationally protected intertidal area. Oystercatchers have been ringed with unique color codes and ongoing research has maintained a high (>90%) proportion of color‐ringed individuals in the breeding population. Low rates of ring wear, along with active replacement of worn rings, mean that almost all color‐ringed individuals remain identifiable (Allen et al., 2019b). At the start of the project (1983), the local breeding population was initially saturated although the Wadden Sea population was still growing (van de Pol et al., 2010c; Ens et al., 2014). The population began steadily declining from the mid‐1990s and has continued declining through the study period (van de Pol et al., 2010c; Ens et al., 2014). When the population, and therefore the number of breeding pairs, began declining in the 1990s, the study area was gradually increased over time to maintain a sufficiently large study population (Ens et al., 2014).

## OYSTERCATCHER POPULATION MODEL

We first introduce our model's state variables, explain how vital rates were estimated and conclude with the construction of the integral projection model. Details of the regressions performed to estimate the parameters are described in Appendix S1, while the details concerning the structure of the IPM kernels are described in Appendix S2.

### State variables

We modeled oystercatcher population dynamics using integral projection models (IPMs), whereby the vital rates can be related to a continuous state variable and to one or multiple discrete state variables (Easterling et al., 2000; Rees et al., 2014). In the IPM (which has a 1‐year time step and a pre‐breeding census), individuals were characterized using three state variables: age (discrete: 1–40), breeding status (discrete: pre‐breeder, breeder, or non‐breeder conditional on having bred at least once), and lay date (continuous). Although we investigated sex differences, these were not included: oystercatchers are genetically and socially monogamous with few instances of polygyny (Heg & van Treuren, 1998), survival differences were small (van de Pol et al., 2010a; Appendix S1), recruitment ages and breeding probabilities were similar (Appendix S1), and offspring sex ratios were near 50% (Heg et al., 2000). Other potential sex‐specific differences in for example chick survival could not be estimated due to lack of sample size. The IPM is thus asexual and our estimates of survival, reproduction, and growth did not distinguish between males and females.

#### Age and breeding status

Oystercatchers have a long life expectancy that can exceed 40, and do not generally mature until age three (Ens et al., 2014). Furthermore, age is related to the state variables of breeding status and lay date: breeding probability initially increases as an individual ages, and experienced breeders tend to have earlier lay dates than inexperienced breeders (van de Pol & Verhulst, 2006). Finally, survival varies between breeding states, and also among subadult age classes and adults: first‐year birds tend to have lower survival than subadults and adults (van de Pol et al., 2006).

#### Lay date

**FIGURE 1:**
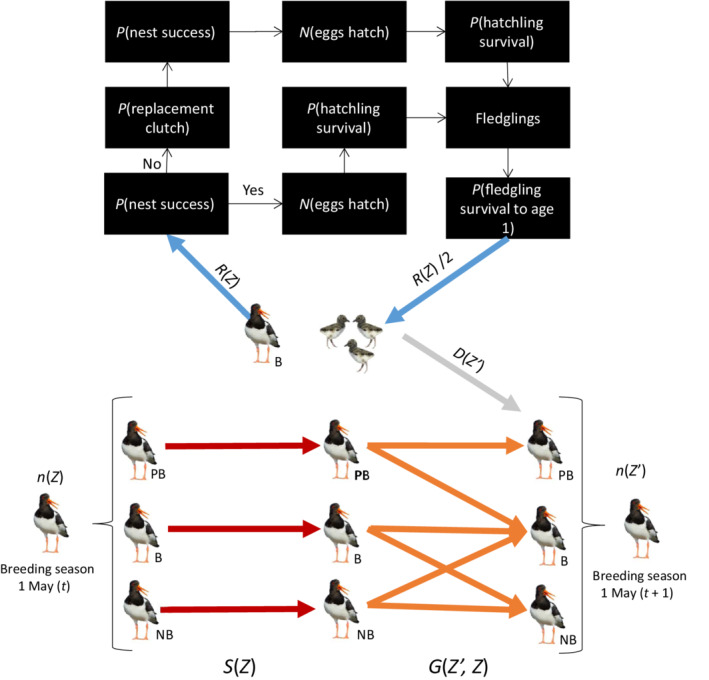
An illustration of the Oystercatcher life cycle together with the structure of the integral projection model. The model describes the development of the population, *n*(*Z*), from time *t* to time *t* + 1, *n*(*Z*′). *S*(*Z*) is survival, *G*(*Z*, *Z*′) is growth, *R*(*Z*) is reproduction, and *D*(*Z*′) is the development of new recruits. *Z* is the state of the individual, which includes age, breeding probability, and lay date. PB is pre‐breeders, NB is non‐breeders, and B is breeders. The reproduction kernel of breeders is expanded to illustrate the reproductive vital rates that comprise fecundity, i.e., individuals recruited to the population at age 1. The number of recruits are divided by two so that estimates are per parent rather than per nest. The variable *p* is the probability (binomial) and *N* is the number of (truncated Poisson)

Individuals with earlier lay dates tend to have higher reproductive performance in single‐brooded species (Daan et al., 1989), which may be due to a direct effect of breeding time (date hypothesis) or an effect of quality differences among individuals (quality hypothesis; Verhulst & Nilsson, 2008). We therefore used regression models to relate not only reproductive vital rates (Figure 1), but also survival to an individual's lay date. We used the lay date of the first clutch as this was most likely to represent an individual characteristic rather than the lay date of replacement clutches that could be influenced by several external factors (notably predation or flooding). The mean lay date of the population may vary from one year to the next due to environmental conditions. Therefore, we standardized lay date so that an individual's lay date was compared to the mean of all lay dates in a given year (i.e., relative lay date). We subtracted the mean lay date for a given year and divided it by the standard deviation of the entire study period so that the variation was comparable throughout the study period.

### Vital rates

The census moment for the population was at the start of the breeding season (May 1), and survival of each breeding state was estimated from the start of the breeding season to the subsequent breeding season (Figure 1). Fecundity included all processes leading to recruited individuals entering the population at age 1 in year *t* + 1 (Figure 1). Rather than a single compound fecundity rate, we decomposed the reproductive cycle into several components (i.e., vital rates; Figure 1) because eggs, chicks, and fledglings may experience different threats. We estimated the probability of nest success, i.e., when at least one egg hatches. Previous studies have indicated that oystercatcher nests have a higher probability of being predated during the egg‐laying phase, and that “complete” clutches with two to three eggs already showed evidence of (partial) predation (Ens, 1991; Jager et al., 2000). Consequently, we estimated the number of eggs that hatched from successful nests (i.e., a truncated Poisson), rather than estimating clutch size and subsequent egg survival. We estimated the survival of hatchlings to the fledgling stage (~28 d) and finally the survival of fledglings to age 1 (Figure 1). If a nest was not successful, we estimated the probability of initiating a replacement clutch (Appendix S1: Section 3.4) and separately estimated the replacement clutch vital rates for each subsequent stage of the reproductive cycle (Figure 1). On rare occasions, oystercatchers may have more than one replacement clutch, but we grouped all replacement clutches of a pair together in the regression analyses to obtain general vital rate estimates of replacement clutches (regardless of whether it was a second, third, etc. replacement clutch). The recruits produced from the first and replacement clutches were summed to arrive at the per‐adult number of recruits that enter the population at age one. Note that we estimate “per‐adult” number of recruits, we therefore divide the number of recruited offspring by two and assume that nests' parents were monogamous (only two parents).

### Integral projection model

All vital rates, which together describe the oystercatcher life cycle, were combined to construct our IPM's projection kernel (Figure 1). The full kernel *K*(*Z*′, *Z*) consisted of two layers

(1) $$K\left(Z^{\prime },Z\right)=P\left(Z^{\prime },Z\right)+F\left(Z\prime ,Z\right)$$

where *P* represents survival and growth from state *Z* to state *Z*′ and *F* represents the production of state *Z*′ offspring by state *Z* parents (Figure 1; Ellner & Rees, 2006). State *Z* consists of one continuous and two discrete states such that *Z* = *Z*_*L*_ + *Z*_*B*_ + *Z*_*A*_, where *L* is a continuous state variable of lay date, *B* is a discrete (binomial) state of breeding status whereby breeder = 1 and pre‐breeder/non‐breeder = 0, and *A* is a discrete state of age (1–40). Throughout the IPM, *Z* refers to the state of an individual in time *t* and *Z*′ is time *t* + 1. The layer *P* can be further described as

(2) $$P\left(Z^{\prime },Z\right)=G\left(Z^{\prime },Z\right)S\left(Z\right)$$

where *S*(*Z*) is the probability that an individual of state *Z* survives from time *t* to *t* + 1 and *G*(*Z*′, *Z*) is the probability that an individual of state *Z* at time *t* grows (i.e., ages and changes) to state *Z*′ at *t* + 1, conditional on survival. The layer *F* can be further described as

(3) $$F\left(Z^{\prime },Z\right)=D\left(Z^{\prime }\right)R\left(Z\right)$$

where *R*(*Z*) is the number of offspring produced that recruit to the population at age one between time *t* to *t* + 1 by individuals with state *Z* at time *t*. *D*(*Z*′) is the development of recruits and describes the state *Z*′ that recruits enter the population with at time *t* + 1. See Appendix S2: Section S4 where we explain why *Z*′ of recruits does not depend upon state *Z* of the parents, i.e., *D*(*Z*′, *Z*).

#### IPM structure

The structure of the IPM can thus be summarized as

(4) $$n\left(Z^{\prime }\right)=\int [G\left(Z^{\prime },Z\right)S\left(Z\right)+D\left(Z^{\prime }\right)R\left(Z\right)]n\left(Z\right)$$

where *n*(*Z*′) is the distribution of individuals with states *Z* (*Z* = *Z*_*A*_, *Z*_*L*_, *Z*_*B*_) at time *t* + 1, *n*(*Z*) is the distribution of individuals with state *Z* at time *t*, and the integral performs a sum over all possible ways (survival, growth and reproduction) of changing from state *Z* at time *t* to state *Z*′ at time *t* + 1.

#### Parameter estimation

Mark–recapture and regression analyses were performed to estimate the model‐averaged survival, growth, and reproduction parameters for the IPM (Appendix S1). These parameters were subsequently used to form the equations for each of the kernels that make up the IPM (Equation 4), which are described in Appendix S2.

## ANALYSES

### Decadal IPM construction

We analyzed the population dynamics of the oystercatcher per decade, a time unit that summarizes the interannual variation in multiple vital rates to identify persistent patterns. The choice of decade also provides a temporal period that is less likely biased (in contrast to time periods chosen based on patterns in a specific vital rate, see example in Appendix S3). We also estimated annual population growth rates but these required several simplications because numerous relationshiops could not be estimated on an annual basis (Appendix S3; Appendix S4: Figure S1). We constructed separate IPMs for the 1980s, 1990s, 2000s, and 2010s in which we could analyze the population dynamics for each decade separately and identify how the vital rates of oystercatchers changed over this period and how this influenced population growth rates.

We also constructed an average IPM containing vital rates averaged over all decades. The average IPM described the population dynamics of 1983–2019, while the four decadal IPMs described the periods of 1980s (1983–1989), 1990s (1990–1999), 2000s (2000–2009), and 2010s (2010–2019).

### IPM simulations

#### Influence of changing vital rates on population growth (λ)

We investigated which vital rates were most influential in terms of changes in λ during the study period. The average IPM for the entire study period was used as a base model, and we subsequently iterated through multiple IPM simulations whereby the parameter (i.e., intercept/slope) of a single decade‐specific vital rate replaced the parameter of the model‐averaged vital rate. For example, in the averaged IPM, we would replace the averaged intercept and slope parameters of nest success with the nest success of the 1980s, simulate the population dynamics and subsequently repeat the process using nest success of 1990s, 2000s, and 2010s. This process was performed for all vital rate parameters related to reproduction, survival and breeding probability (Figure 1). To evaluate the effect on λ, we simulated the population dynamics for *t* number of years, where *t* is the time required for λ and the age distribution to stabilize. Due to the high number of stages (40 age classes × 3 breeding states × 100 lay date bins = 12,000 stages), we did not calculate population projection analytically as is normally done with matrix models. The simulations were started with a population vector containing one individual in each of the 12,000 classes. From each simulation, we extracted λ, stable age distribution, the number of new recruits and population size. In this way, we could quantify the individual contribution of each vital rate to the overall population growth rate.

#### Reversing the population decline in the last decade

We subsequently investigated how an improvement in certain vital rates could reverse the population decline and return the λ of the last decade (2010s) above 1, i.e., to a growing population. Based on our results, we considered the vital rates of nest success, hatchling survival, and adult survival because these vital rates had declined in the last decade and explained more variation in λ than other vital rates (see *Results*). We first identified how λ of the last decade, 2010s, changed if nest success, hatchling survival and adult survival returned to previous levels (i.e., 1980s, 1990s, and 2000s), including how these three vital rates were related to lay date. We then performed simulations in which each vital rate was increased by 0.01 from the 2010s value up to a predefined maximum value. The maximum increase we considered from 2010 levels was 0.20 for nest success, 0.10 for hatchling survival, and 0.05 for adult survival. Vital rates were therefore increased to levels of previous decades, and even higher values. Given that improvements may be needed in more than one vital rate, we also considered simulations in which one of the other vital rates, or both, had already returned to 1980s levels (i.e., when the population was stable, see *Results*) while the vital rate of interest ranged between the 2010s value and the predefined maximum. For example, if nest success was the vital rate of interest, we performed simulations in which hatchling survival, adult survival, or both were at 1980s levels and the values of nest success ranged between the nest success of the 2010s and the predefined maximum.

#### Simulating changes in lay date on population growth

Lay date varied interannually, but no trend was detected in terms of oystercatcher lay dates through the study period (Appendix S4: Figure S1). Meanwhile, environmental and climatic conditions have been changing in the study area, for example, the increasing frequency of flooding events towards the end of the breeding season (van de Pol et al., 2010b) and increasing winter temperatures that influence food availability in the breeding season (van de Pol et al., 2010c). Given the relationships we identified between lay date and oystercatcher vital rates (Appendix S1), and the changing environmental conditions on Schiermonnikoog, we simulated how a shift in the mean lay date of the population may influence population growth rates. We simulated 40 alternative lay date distributions by either advancing or delaying the mean lay date of the population by 20 d (in 1‐d increments and equating to approximately two standard deviations from the mean; Appendix S4: Figure S2). As per previous simulations, the IPM was iterated *t* timesteps until λ stabilized. The λ was extracted to compare how a shift in the mean lay date of the population may influence the population's development.

## RESULTS

### Influence of changing vital rates on population growth

**FIGURE 2:**
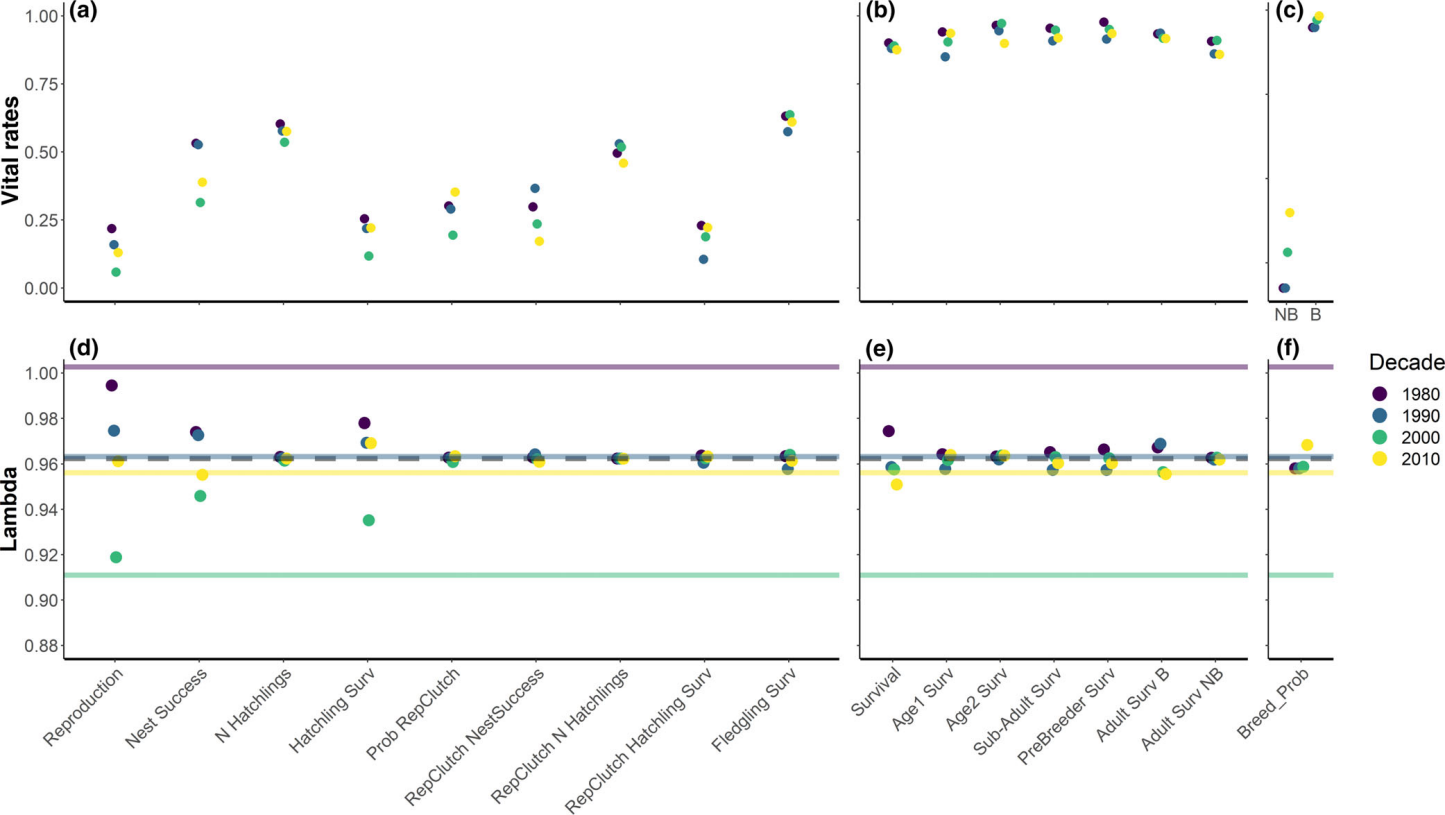
(a, b, c) Vital rates and (d, e, f) population growth rates (lambda) for each decade split between reproduction (a, d), survival (b, e), and breeding probability (e, f). Almost all vital rates (a, b, c) were bounded between 0 and 1, except number of hatchlings, which was normalized for visualization purposes by dividing by 4 (in general the maximum clutch size). Vital rates of breeding probability (c) are shown for non‐breeders (NB) and breeders (B) but combined in the population growth rate simulations (f). Population growth rates (b, d, e): The colored solid lines depict lambda from the integral projection models (IPMs) for each decade, while the dashed line is the lambda from the average IPM for the entire study period. The colored points should be compared to the dashed line (average IPM) and depict the change in lambda when a vital rate is changed from the average values of the study period, to the decade‐specific parameter instead. Colored points that are further from the dashed line indicate a greater relative contribution with either a higher/lower lambda (above/below the dashed line). The *x*‐axis labels “Reproduction” and “Survival” combine all appropriate parameters (i.e., those that follow in the panel). Abbreviations: N Hatchlings, number of hatchlings; Hachtling Surv, hatching survival; Prob RepClutch, probability of replacement clutch; RepClutch NestSuccess, nest success of replacement clutch; RepClutch N Hatchlings, number of hatchlings from replacement clutch; RepClutch Hatchling Surv, hatchling survival of replacement clutch; Fledgling Surv, fledling survival; Age1 Surv, survival from age one to two; Age2 Surv, survival from age two to three; Sub‐Adult Surv, survival from age one to three (i.e., Age1 and Age2 combined); PreBreeder Surv, survival of all pre‐breeder age classes; Adult Surv B, survival of adult breeders; Adult Surv NB, survival of adult non‐breeders; Breed_Prob, breeding probability

The projected annual population growth (λ) of the averaged IPM for the entire study period of 1983–2019 was 0.962, indicating a population decline of 3.8% per year. The rates of population growth varied among decades and was stable in the 1980s, while a decline began in the 1990s of 3.7%, before dramatic declines followed in the 2000s of nearly 9% per annum. Population growth rates recovered somewhat in the 2010s, albeit with the population still declining at 4.4% per annum (Figure 2). These decadal population growth rates are consistent with previous studies and the decline in the number of breeding pairs in the study area (Appendix S5). Population growth rates were also similar between the sex‐specific IPMs (<0.01 difference among lambdas), which included sex‐specific values of adult survival and breeding probability (Appendix S6).

Vital rates associated with the reproduction phase explained most decadal variation in λ, especially nest success and hatchling survival (Figure 2). Worsening fecundity rates account for most of the decline in λ in the 2000s (Figure 2a,d). Nest success in the 2000s reduced the average λ by 0.02 while hatchling survival reduced average λ by 0.03 (Figure 2d). However, other vital rates are also important to consider. Fecundity improved in the last decade (2010s), especially hatchling survival, along with improved breeding probabilities and subadult survival (Figure 2). Adult survival of breeders was similar in the 1980s and 1990s (0.936 and 0.937, respectively) but deteriorated in the 2000s and 2010s (0.918 and 0.917, respectively). The decline in breeding adult survival probability of 0.02 in the 2010s reduced average λ by 0.009 in comparison with the combined action of all reproduction parameters of the 2010s, which reduced average λ by 0.001 (Figure 2d,e).

### Reversing the population decline in the last decade

**FIGURE 3:**
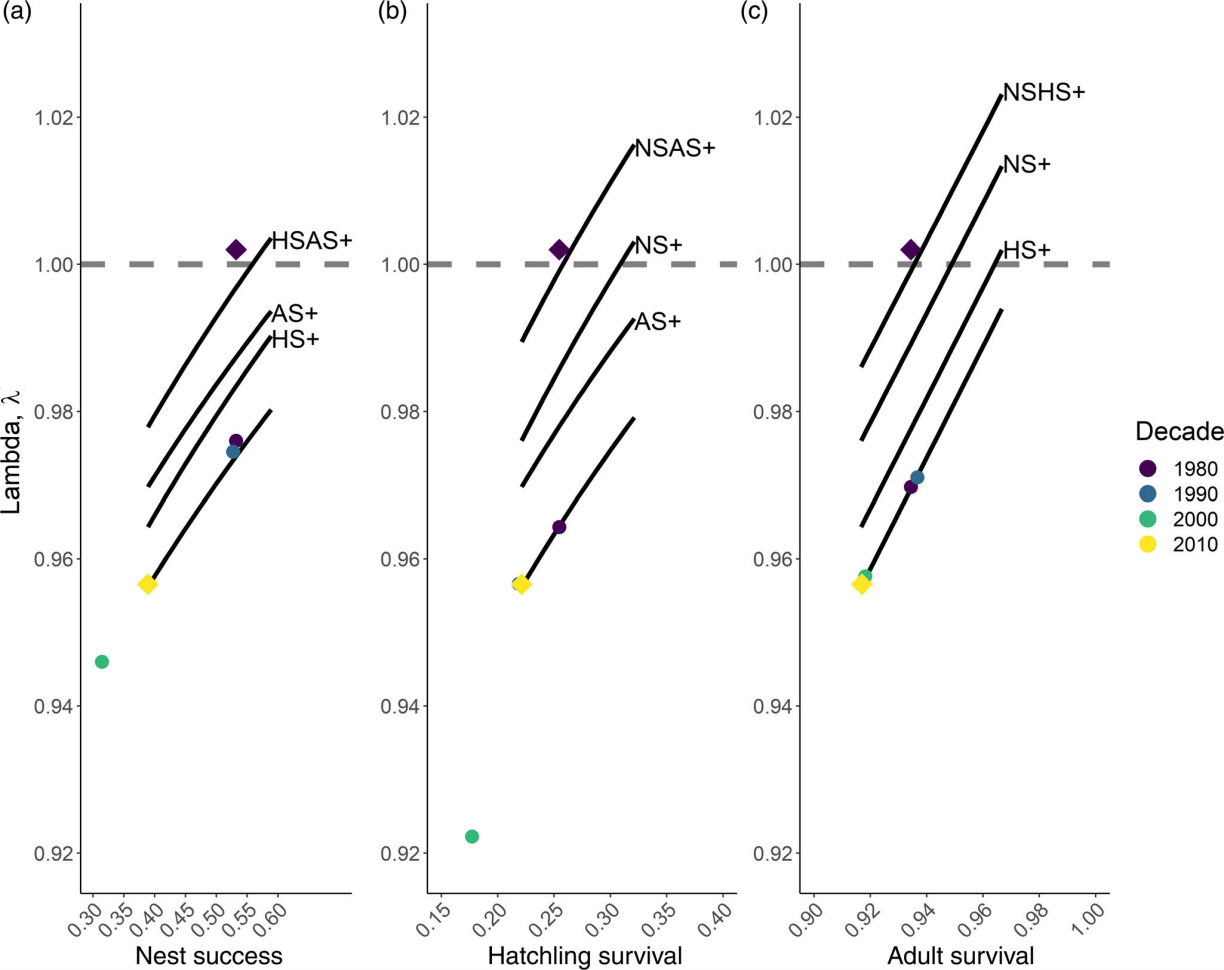
Response of population growth in the last decade (2010s) if key vital rates of (a) nest success, (b) hatchling survival, and (c) breeding adult survival, were to improve to the value on the *x*‐axis (solid line). Scenarios are also depicted for the vital rate in each panel in which another vital, i.e., nest success (NS+), hatchling survival (HS+), adult survival (AS+), or a combination, had already returned to the levels of the 1980s. The yellow and purple diamonds are the lambda and vital rates of the 2010s and 1980s IPM, respectively. The colored points indicate how population growth would change if that specific vital rate returned from 2010 values to the value of a previous decade (1980, 1990, 2000). Note that these points do not fall on the solid line because previous decades had a different relationship with lay date compared to the 2010s. The dashed horizontal line indicates λ = 1

Given the vital rates of the oystercatcher population in the last decade (2010s), an improvement in a single vital rate would not yield a λ above 1, even if the vital rate increased above historical levels (over the range that we considered; Figure 3). Our simulations outline how population growth rates respond to improvements in just a single vital rate, or in combination with an improvement of other vital rates to levels observed in the 1980s (Figure 3). Halting the decline of the oystercatcher requires an improvement in at least two vital rates. Unless the vital rates exceed levels observed previously, then realistically all three vital rates nest success, hatchling survival, and breeding adult survival would need to return to levels of the 1980s for stable population growth rates (Figure 3). Nest success, hatchling survival, and breeding adult survival averaged 0.532, 0.255, and 0.936, respectively in the 1980s, compared with 0.389, 0.221, and 0.917 in the 2010s. Should all these three vital rates return to the level of the 1980s, λ would increase to 0.999.

### Simulating changes in lay date on population growth

The mean lay date of the breeding population on Schiermonnikoog was May 22, with the earliest mean lay date in 2011 on May 17, and the latest in 2007 on May 28 (Appendix S4: Figure S3). Average lay dates did not advance significantly during the study period (β = −0.018 per year, *p* = 0.69; *R* ^2^ = 0.005) and a quadratic relationship only provided a minor improvement to model fit (AIC 190.0 vs 190.5) but neither term was significant (*p* > 0.05, *R* ^2^ = 0.06). Average lay dates varied significantly among decades (ANOVA; *F*_3,5848_ = 30.67, *p* < 0.001) but the absolute differences were only minor: compared to the 1980s, lay dates were on average 2 d later in the 1990s and 2000s and 1.2 d earlier in the 2010s (Appendix S4: Table S1).

**FIGURE 4:**
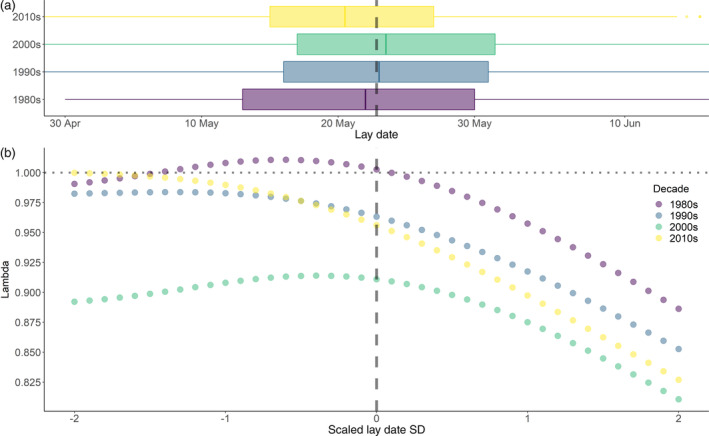
Influence on population growth rate (λ) if mean lay date of the breeding population were to become earlier or later. (a) Box plot showing distribution of lay dates in each decade. The box includes the interquartile range and the whiskers 95%. The solid line is the median and points are outliers. The range of the *x*‐axis was trimmed to match that of plot (b) and hence excludes some early or late nests (i.e., >2 SD). (b) Results of IPM perturbations where the mean lay date was incrementally shifted by 1 d (solid points for each perturbation) to a max of 20 d either side of the current mean. The vertical dashed line is the mean lay date during the entire study period and the dotted horizontal line indicates a stable population growth rate (i.e., λ = 1)

Our simulations that shifted the mean lay date of the population by up to a maximum of 20 d (later or earlier), in increments of 1 d, revealed contrasting patterns among the four decades. The average lay date of the 1980s and 2000s was close to the level that yielded maximum population growth, with both decades exhibiting reduced growth rates for earlier lay dates (Figure 4). In contrast, the 1990s and 2010s did not have a parabolic relationship, instead growth rates peaked for early lay dates, with a higher peak in the 2010s compared to the 1990s. Theoretically, a stable population growth rate could be achieved for the 2010s with an advance in lay date alone, but this would require an advance of 2 weeks (Figure 4). The regression analyses we performed in estimating the relationship between the vital rates and lay date (Appendix S1), show that nest success and hatchling survival were the most important variables for explaining this pattern, along with the probability of a replacement clutch (Appendix S1). These three vital rates had significant model support for decadal variation in their relationship with lay date (Appendix S1: Tables S10, S14, S16). The decade‐specific reproduction kernels of the IPM illustrate how the date for the peak number of recruits per adult in relation to lay date has advanced, meaning fewer birds in the population have high success (Appendix S1: Figure S15).

## DISCUSSION

The population trend of the studied oystercatcher population over the last four decades generally mirrors that of the global population, from initial stability to variable rate of decline, resulting in its near‐threatened status today. Despite a persistent decline in the last three decades, the demographic mechanisms of decline have been altered: as found in previous research of oystercatchers, lower fecundity rates explained most variation in the decline of the projected annual population growth rate (λ) in the 1990s to 2000s. However, these rates improved in the 2010s and it is the decline in adult survival that contributed the most in the decline in *λ* in the last decade. Our analysis emphasizes how demographic drivers of population dynamics can change and demonstrates how the importance of key demographic variables has varied over time. Understanding this complexity is required for identifying causes of decline and recovery strategies, especially given that our results indicate that threats have not remained constant over time. The dynamic nature of the threat landscape is further supported by the finding that the average individual (in terms of lay date) no longer has the highest performance in the population, and thus emphasizes how individual heterogeneity in vital rates can play an important role in modulating population growth rates.

### Causes of oystercatcher declines since the 1990s

Contrasting demographic mechanisms of population growth are well known among species with cyclic population dynamics (Myllymäki, 1977; Oli & Armitage, 2004), but has been less often described for long‐lived species with persistent declines. Research on seabirds have shown how a population of Emperor Penguins (*Aptenodytes forsteri*) crashed due to declining survival (Barbraud & Welmerskirch, 2001; Jenouvrier et al., 2005) and did not recover because of variable breeding success (Jenouvrier et al., 2009). Similarly, research on the Gray Partridge (*Perdix perdix*) showed that, although a change in a single vital rate explained the species' decline, an improvement in multiple vital rates was required to achieve stable population growth rates (Bro et al., 2000). These studies highlight the need for understanding the demographic mechanisms of population change so that effective conservation actions can be identified (Selwood et al., 2015). The need to understand the demographic mechanisms of population change are emphasized by our findings, the demographic drivers of decline have changed over time while halting the decline of the oystercatcher cannot be achieved with improvements in any single vital rate.

In line with life history theory of long‐lived species, oystercatchers likely prioritize their own survival over reproduction (Ens et al., 2014; Gaillard & Yoccoz, 2003; Pfister, 1998). Adult survival can vary considerably from 1 year to the next due to, for example, severe winters (Appendix S1; Allen et al., 2019a; van de Pol et al., 2010c), but over longer temporal scales (e.g., decades), we show that adult survival had low temporal variation as expected by the environmental canalization hypothesis. We also found low levels of individual variation in adult survival based on our state variables of lay date and sex, neither of which were important for explaining variation in adult survival and instead most variation was explained by breeding status, although variation in non‐breeder survival had minimal influence on λ (Figure 2; Appendix S1). Our results do however raise concerns that adult survival of breeders fell in the last two decades, and despite the minor decrease in survival (0.02) it had a relatively large effect on population growth rates. These results are especially concerning when considering predictions that, instead of decreasing, adult survival should actually increase following milder winters under climate change (van de Pol et al., 2010c). The decline in survival suggests that cold and/or severe winters are no longer a driver of winter adult mortality, which requires further research about the cascading effects of mild winters. For example, survival may have declined in recent years due to climate change reducing reproduction but increasing adult survival of cockles (Beukema & Dekker, 2020), and aging shellfish stocks may increase the risk of bill damage in oystercatchers foraging on such perilous prey (Rutten et al., 2006). Given the decline in adult survival in recent decades, understanding the causes should be a priority for future studies.

While low reproductive output was already known to be an issue for the population (Roodbergen et al., 2012; van de Pol et al., 2014), our results emphasize the importance of splitting reproduction into the different stages and revealed that the main driver of decline was low nest success and hatchling survival (but see Appendix S3 where hatchling survival was not a main driver when using demographic‐based time periods instead of decadal ones). These vital rates were especially low in the 2000s, and while hatchling survival has recovered somewhat, nest success has only had marginal improvements. Hatchling survival may be related to availability of preferred prey during the breeding season in the study area, namely ragworm (*Hediste diversicolor*) and Baltic tellin (*Limecola balthica*; Heg & van der Velde, 2001, van de Pol et al., 2010c). Ragworm abundance is negatively related to winter temperatures, which have increased in recent years (van de Pol et al., 2010c). While Baltic tellin declined at the turn of the century (Beukema & Dekker, 2019; Drent et al., 2017), it has been increasing in the last decade (Drent et al., 2017), which may partially explain the higher hatchling survival in the 2010s. Nest success, however, remains low and is the main driver of low fecundity today. The last two decades experienced increasing frequency and magnitude of extreme climate events, namely flooding of nests (van de Pol et al., 2010b). Nests are not flooded every year though, while nest success remains low, meaning that other factors also need to be considered such as changes in predator densities or how effects experienced during winter may carry over to influence reproductive success in the summer (Ens et al., 2014). Whilst some contributing causes of the oystercatcher decline have already been identified, and new research may identify other contributing causes of decline, data may not be available to fully understand what drove past population changes. This highlights the importance for population studies to consider from an early stage onwards which environmental variables should be measured so that these can be related to changes in population dynamics and thus inform conservation actions.

### Individual heterogeneity in vital rates and population growth

Our state variables explained significant variation in vital rates and especially lay date was important for explaining individual variation in reproduction. The 1980s had a parabolic relationship with average‐timed individuals producing more young, but the last decade had a monotonic decreasing relationship, with early‐nesting individuals producing the most young. Our simulations suggest that based on the vital rates of the last decade, population growth rates could theoretically increase by 4% to stable levels if the average population lay date were to advance by 2 weeks (all else being equal). However, average lay dates have scarcely advanced a day in the last four decades, highlighting how environmental changes are influencing individual variation in vital rates, through its impacts on individuals with average lay dates, and subsequently impacting population growth rates. Our results therefore raise concerns about how local environmental conditions are influencing the success of the average bird in the population. Increasing risk of flooding events is a known threat that is also directly tied to when individuals initiate their clutch, as the risk of nest flooding increases through the season (Bailey et al., 2017; van de Pol et al., 2010c), but this would not explain why the success, in absolute terms, of early birds would increase (Appendix S1: Figure S15). Alternative environmental drivers thus need to be identified and an understanding of which environmental changes have driven the change in the relationship between lay date and reproduction may be key to identifying additional causes of low reproduction.

## CONCLUSION

Our analysis identifies how the demographic causes for a species experiencing a persistent population decline have varied over time: information that is important for understanding whether past threats continue to affect a species and which threats remain relevant today. In the case of the oystercatcher, nest success is clearly an important vital rate: it has declined dramatically, remains low today and has been one of the key demographic cause of decline. In this regard, individual heterogeneity in vital rates clearly influences population growth: the relationship between lay date and reproduction has changed, whereby the average individual is no longer successful and instead earlier birds do better. These findings draw parallels with research on phenological mismatches and climate change, and calls for a deeper understanding of how environmental conditions interact with individual lay dates to influence reproductive success. However, it would be wrong for conservation actions to only be oriented towards a single phase of the oystercatcher's life history. Although improvements will help to slow the rate of population decline, restoring single vital rates like nest success to historical levels will not halt the decline. Improvements are also needed in other demographic stages like hatchling survival and adult survival. Furthermore, a comprehensive overview is required of how environmental changes impact different stages and vital rates of a species' life cycle simultaneously.

## CONFLICT OF INTEREST

The authors declare no conflict of interest.

## Supporting information


Appendix S1

Appendix S2

Appendix S3

Appendix S4

Appendix S5

Appendix S6

## Data Availability

All data and code (Allen et al., 2021) are available from Data Archiving and Networked Services (DANS) EASY at: https://doi.org/10.17026/dans-245-vfzd.
