# Supplementary material for: The demographic causes of population change vary across four decades in a long‐lived shorebird
Source: Ecology. 2022 Mar 3;103(4):e3615. doi: 10.1002/ecy.3615 (PMC9286424; doi:10.1002/ecy.3615)
Supplement: Supplementary file 1 — Appendix S1 [file ECY-103-0-s001.pdf]

The demographic causes of population change vary across four decades in a long-lived shorebird

Andrew M. Allen, Eelke Jongejans, Martijn van de Pol, Bruno J. Ens, Magali Frauendorf, Martijn van de Sluijs, Hans de Kroon

Ecology

Appendix S1 – Model Selection for Vital Rates

SECTION S1 - SURVIVAL S(Z)

We performed a capture-mark recapture (CMR) analysis to estimate survival of oystercatchers in relation to the state variables of age (Z_A), breeding status (Z_B) and lay date (Z_L). Survival was investigated using a Multistate Live & Dead recoveries model. We only present model results and model comparisons for the survival parameter. The parameters of resighting probability (p), dead recovery (r) and transition (Psi) were investigated beforehand. The top resighting and dead recovery structure included the variables breeding state and an interaction between breeding state and time (i.e. stratum + stratum:time) whilst transition probability took the structure of stratum:tostratum + stratum:tostratum:time. This structure was therefore consistent when investigating the alternative model structures for the survival (S) parameters. Results of the alternative model structures for resighting, transitions and dead recovery are however presented in the survival results section below. The age class variables varied, with only adult age classes (3+) for adult survival (i.e. of breeders and non-breeders), and alternative age structures were considered for fledgling and pre-breeder survival (see section *Pre-Breeder survival models*). Note that first year survival is equivalent to fledgling survival (see *Reproduction*). In addition, the age of oystercatchers is not measured in calendar years (CY), which is traditionally done during bird ringing for example, but in whole years to our census moment on May 1.

The vital rate functions of the IPM only consider the model coefficients and not the standard errors around these coefficients. Therefore, to account for model uncertainty in situations where more than one model receives statistical support, we used model-averaging so that all supported models contribute to the final coefficient. The weighting was based on Akaike weights and all models were included since models with little to no support will have a low weighting. It is not possible to average the beta coefficients in Mark nor RMark (currently it is only possible to average the real parameter estimates; Cooch and White 2019), therefore we used the par.avg function in the r package MuMIn (Barton 2020) to average each of the parameters in the final models. The function included the parameter estimates, their standard errors and the Akaike weights. We estimated the full average, as opposed to a conditional average, which assumes that a variable is included in every model and hence the coefficient is set to zero in models where a variable does not occur (Barton 2020). To verify our approach, we compared the predictions of our model-averaged betas to that of the model-averaged real

estimates from the RMark function *covariate.predictions* and there was almost perfect overlap.

Two separate datasets were used to estimate survival. The first only included oystercatchers that were ringed before fledging and where the lay date of the nest from which they hatched was known (Table S1). We used this dataset to estimate survival for fledglings and pre-breeders. The sample size of this dataset was too small to also estimate decade-specific adult survival once the fledglings matured, and whether adult survival was related to the lay date of future breeding attempts. However, the models clearly indicated differences in survival between sub-adult and adult age classes, therefore we used a separate dataset to estimate adult survival (Table S1). Adults included birds that were known to be adults (i.e. either ringed or observed as adults; Table S1), and had bred at least once in the study area, so that we could relate survival to the lay date of adult's breeding attempts. In total 1,328 adults were observed to breed at least once in the study area and were colour-ringed (Table S1). The number of adults in the dataset remained high across decades, and numerous individuals were observed nesting in multiple decades. Another 529 adults were colour-ringed but were never observed breeding. These adults were included to aid estimates of non-breeder survival but were excluded from any lay date effects. The census moments for the mark-recapture model were May, June and July, i.e. the breeding period. Observations of oystercatchers were collected during field studies, and given the high observation effort, breeding individuals were almost always identified, the protocol of which is described in Van De Pol et al. (2006). The breeding status of an individual could thus be accurately determined from the field observations. We supplemented the resighting dataset with observations from an online database wadertrack (www.wadertrack.nl). Wadertrack includes citizen science observations, and given that the status of breeders was known, the additional observations improved knowledge about the status of non-breeding birds.

Misreading of rings is an issue that may bias survival estimates (Tucker et al. 2019). The field-collected data likely includes few to no misreadings since the status of an individual (i.e. breeder/non-breeder) is the summary of many observations which were typically performed by two observers (at least three careful observations but individuals will often have >100 observations). Out of the ordinary observations were also double and triple checked before entering the database (e.g. new settlements). Observation effort was lower for non-breeders but, like the recommendations of Tucker et al. 2019, single observations of non-breeders were excluded from the database unless it was accompanied by a remark that the observer was sure of the observation. The risk of misreadings is however higher in the citizen science data, and following the recommendations of Tucker et al. (2019), we excluded all single observations of individuals (n = 544) and only included instances where at least two observations of a bird was made. Misreadings due to ring wear or loss are unlikely to be an issue given the low rates of wear and loss in this population (Allen et al. 2019) along with active replacement of rings during the study period.

Section S1.1 Fledgling and sub-adult survival models

We investigated whether fledging and sub-adult survival was related to the lay date of the nest that they were recruited from. We also determined whether survival differed between decades, whether pre-breeder survival was age-specific, and whether the relationship between lay date and survival varied across decades. We considered four alternative age classes for pre-breeders, including a numeric variable for age (i.e. 0 to 40), an age class that included first year, second year, third year and above (agebin123), an age class that separated survival between the third and seventh year (agebin1237; by which time most pre-breeders are not sighted anymore) and a final age class that estimated each age separately up to age seven, after which the ages were combined (agebin1to7). The model included two states, breeder and pre-breeders, with only one-way transitions in that pre-breeders could become breeders (from age three onwards) but breeders could not become pre-breeders. Survival was only estimated for a single age class of breeders, i.e. the different age variables described were only applied to the pre-breeding state. We considered models where lay date influenced first year survival, first and second year, or first through to third year survival. We did not expect survival to only influence later years (e.g. second year) without also influencing first year survival. We did however consider models where the slope of the relationship between survival and lay date may vary with each age class, or a single slope coefficient. To model these effects, we created five dummy variables that were coded as “1” when the effect of lay date should be included and “0” when not. The five dummy variables were Age0, Age1, Age2, Age01, Age012, where Age0 is survival after fledging in the first year, Age1 is survival from age one to two, Age2 is survival from age two to three, Age01 is a single variable for the first two years and Age012 is a single variable for the first three years. The dummy variables were included as an interaction in the model so that only the desired age class was related to the lay date (e.g. Age0:LayDate). Since lay date is an individual covariate, we took this step to avoid lay date influencing survival probability across the entire lifespan of the individual.

Table S1 - Sample sizes for mark-recapture analyses of survival for fledglings in the first year, and sub-adults (second and third year) with known lay date of nest (Survival_FL). Exact sample sizes of sub-adults are unknown because relating survival of sub-adults to lay dates meant they had to survive the first year. In total 1328 adults (Survival_AD) were colour-ringed and had known lay dates; sample sizes reflect individuals with known lay date per decade. A further 529 individuals were observed on the island but never bred in the study area. Sample sizes are also shown for individuals where the sex was known (n = 1,303) for F = Females (n = 657 and M = Males (n = 646). Non-breeding individuals with no lay date included 122 females, 132 males and 275 of unknown sex. Fecundity is the number of nests that were followed, providing info for all vital rates described in Figure 1 (except fledgling survival).

Vital Rate	1980s	1990s	2000s	2010s
Survival_FL	126	457	80	190
Survival_AD	566	579	482	429
Survival_F	279	287	254	214
Survival_M	272	288	222	212

Fecundity	1112	2114	1261	1365

Section S1.1.1 Fledgling survival results

Several models exhibited strong support for explaining variation in fledgling and pre-breeder survival (Table S2). These included models with lay date effects on survival in the first two and first three years, along with age effects. A model with ages estimated separately for pre-breeders to age seven (Agebin1to7) showed stronger support than a combined age group for adults (Agebin123) but had very low support when comparing decadal variation (Models 9 and 18). Decade effects in interaction with lay date were therefore only considered for Agebin123. Survival of fledglings was lowest, with survival increasing for second year and again for third year birds (Table S3; Figure S1), with birds hatching from nests with early lay dates having higher survival (Figure S1). In addition, survival of fledglings and second year birds tended to be lower in the 1990s (Table S3; Figure S1) whilst pre-breeders had the highest survival in the 1980s. Note that due to data limitations, survival of pre-breeders could not be estimated for birds aged older than seven in the 1980s, given that our starting year was 1983. The parameters for age six birds were therefore used for this class, which had near identical overlap in the other decades (Figure S1g and h).

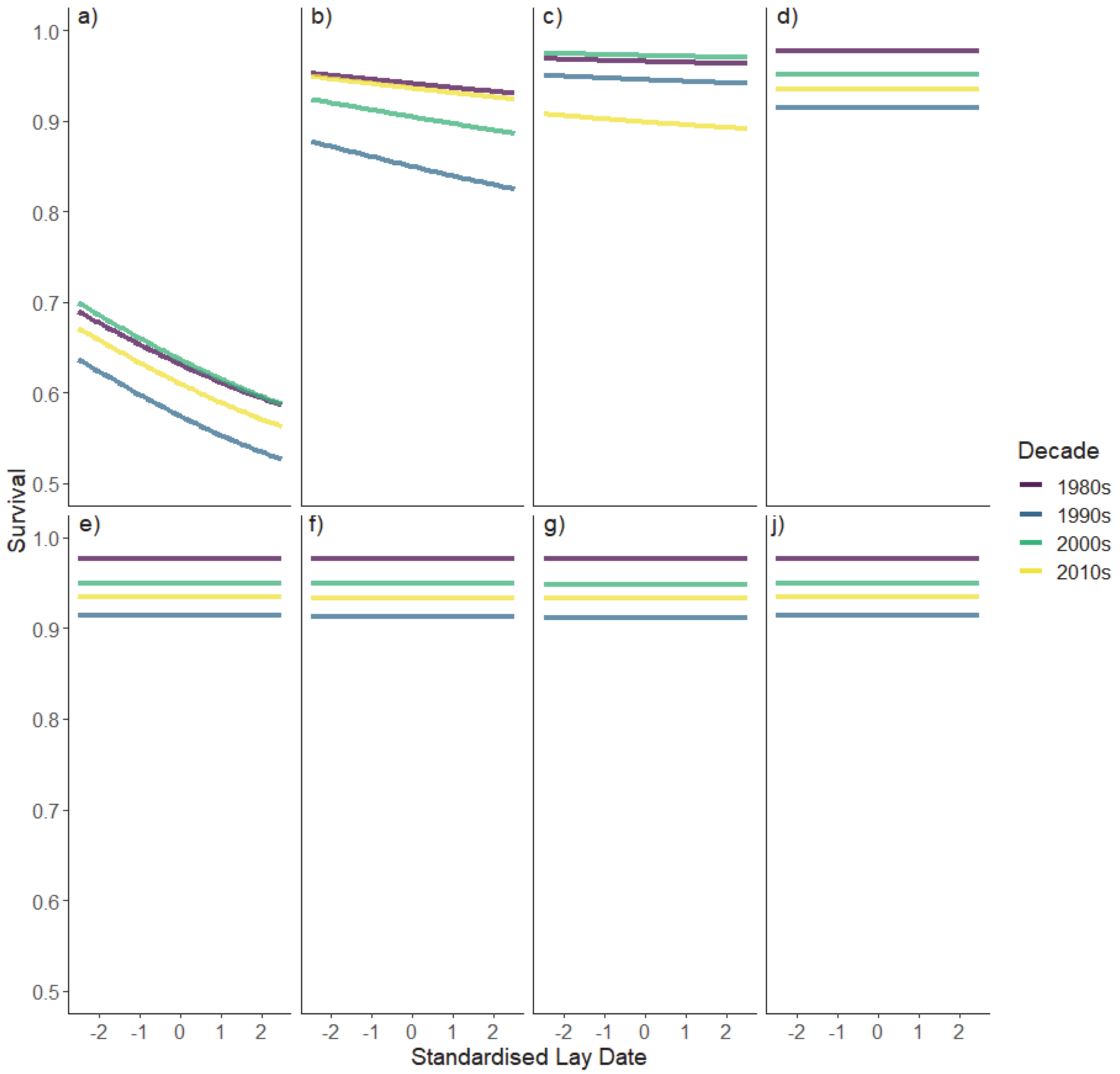

Figure S1 – Predicted survival of pre-breeders per age class including a) first year, b) second year, c) third year, d) fourth year, e) fifth year, f) sixth year, g) seventh year and h) eighth year and older birds, ringed as fledglings, in relation to the standardised lay date of the nest they hatched from. Coefficients were averaged from all considered survival models (Table S2) and weighted by the Akaike weight. Confidence intervals have been excluded since these are wide and overlapping (e.g. Figure S2), and only the modelled relationship shown is used in the IPM. Standard errors of the model-averaged coefficients are shown in Table S3.

Table S2 –Model comparison results of a Multistate live and dead recoveries model for the fledgling and pre-breeder survival parameters. All models contained the same structure of resighting probability = stratum + stratum:time, dead recovery = stratum + stratum:time and transition = stratum:tostratum + stratum:tostratum:time. Nr is the model number, Model is the structure of the survival parameters, npar is the number of parameters, ΔAICc is delta AIC (the top model had an AICc of 10815.84), w_i is the Akaike weight. Agebin123 consisted of four age classes, namely fledglings (Age0), Age1, Age2 and adults, Agebin1237 included an additional agebin to separate survival of pre-breeders aged three to seven, Agebin1to7 had

separate bins for each age from one to seven and combined older age classes and Age was a numeric variable for age. All models shown estimated survival of breeders separately and thus included an interaction with stratum. LD is lay date. “Age” is the dummy variable for either each age separately (Age0, Age1, Age2) or combined (Age01, Age012).

Nr	Model	npar	ΔAICc	w_i
1	~Agebin123 * Dec + Age01:LD	201	0.00	0.22
2	~Agebin123 * Dec + Age012:LD	201	0.34	0.18
3	~Agebin1to7	179	1.21	0.12
4	~Agebin123	174	1.28	0.12
5	~Agebin123 * Dec + Age01:LD + Age01:LD^2	202	1.95	0.08
6	~Agebin123 * Dec + Age012:LD + Age012:LD^2	202	2.28	0.07
7	~Agebin123 * Dec + Age0:LD	201	3.01	0.05
8	~Agebin37	175	3.33	0.04
9	~Agebin123 * Dec	201	4.43	0.02
10	~Agebin123 * Dec + Age0:LD + Age0:LD^2	202	4.69	0.02
11	~Agebin123	176	5.08	0.02
12	~Agebin123 * Dec + Dec:Age01:LD + Dec:Age01:LD^2	208	5.16	0.02
13	~Agebin123 * Dec + Dec:Age01:LD	204	5.21	0.02
14	~Agebin123 * Dec + Dec:Age012:LD	204	6.21	0.01
15	~Agebin123 * Dec + Dec:Age012:LD + Dec:Age012:LD^2	208	8.59	0.00
16	~Agebin123 * Dec + Dec:Age0:LD	204	8.85	0.00
17	~Agebin123 * Dec + Dec:Age0:LD + Dec:Age0:LD^2	207	9.36	0.00
18	~Agebin1to7 * Dec	214	29.23	0.00
19	~Stratum + PB:Age + PB:Age2	174	99.60	0.00
20	~Stratum + PB:Age	173	156.45	0.00
21	~Stratum*Age	174	164.82	0.00
22	~Age	173	262.79	0.00
23	~Stratum	172	348.47	0.00
24	NULL	171	424.19	0.00

Survival models that included an interaction with lay date were well supported based on AICc (Table S2), but these patterns were consistent across decades with a general declining survival for birds hatching from later nests (Figure S2). Decadal differences in this relationship were not well supported, and instead only differences in the intercept were well supported with lowest fledgling survival in the 1990s.

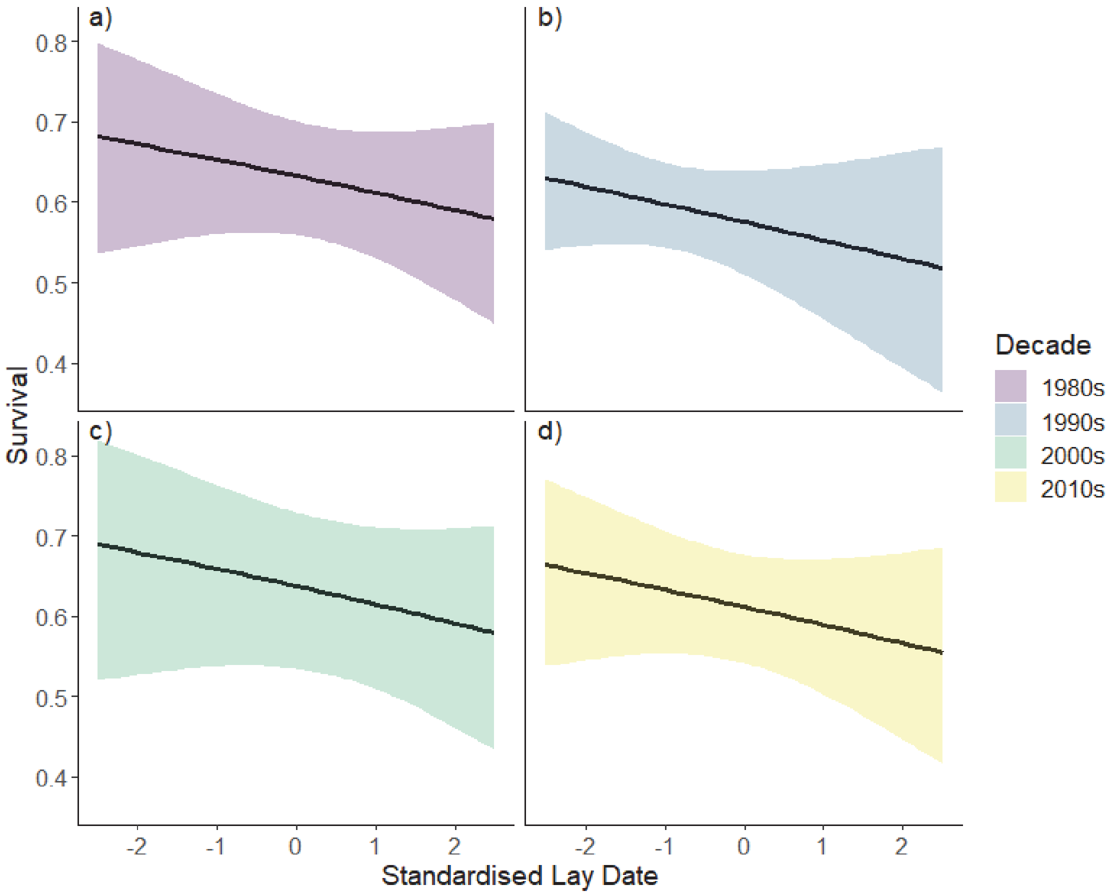

Figure S2 – Model averaged predictions for first year survival of fledglings in relation to (standardised) lay date of the nest from which they hatched in a) 1980s, b) 1990s, c) 2000s and d) 2010s. Results for each decade are shown in separate panels due to the large (and overlapping) confidence intervals, which are colour coded for clarity. The solid line depicts the modelled relationship between survival and lay date.

Table S3 – Model-averaged coefficients used in the IPM for survival of fledgling, sub-adults and pre-breeders in relation to decade and lay date. The intercept is the 1980s and fledglings (i.e. first year birds). Age0 is first year survival, Age1 is second year survival, Age2 is third year survival et cetera to Age7+ which is the final age class of pre-breeders. Note that survival of adult breeders and non-breeders is described below (see *Adult Survival*). SE is the standard error, LCL and UCL are the 95% lower and upper confidence limits.

Parameter	Coefficient	SE	LCL	UCL
Age0 (Intercept)	0.54	0.54	-0.52	1.60
Age1	2.23	2.31	-2.29	6.76
Age2	2.79	2.82	-2.73	8.32
Age3	3.22	3.32	-3.29	9.73
Age4	3.20	3.34	-3.34	9.74
Age5	3.19	3.33	-3.34	9.73
Age6	3.18	3.34	-3.36	9.71

Age7+	3.21	3.32	-3.30	9.72
1990s	-0.24	0.32	-0.87	0.39
2000s	0.02	0.05	-0.08	0.13
2010s	-0.09	0.12	-0.32	0.14
Age1_1990s	-0.80	1.10	-2.95	1.35
Age1_2000s	-0.55	0.76	-2.03	0.93
Ag1_2010s	0.00	0.00	0.00	0.00
Age2_1990s	-0.24	0.34	-0.91	0.42
Age2_2000s	0.20	3.87	-7.38	7.78
Age2_2010s	-1.06	1.45	-3.91	1.79
Age3-7+_1990s	-1.14	1.57	-4.22	1.93
Age3-7+_2000s	-0.83	1.13	-3.03	1.38
Age3-7+_2010s	-1.00	1.35	-3.65	1.65
Age0:LD:_1980s	-0.09	0.13	-0.34	0.16
Age0:LD:_1990s	-0.09	0.13	-0.34	0.16
Age0:LD:_2000s	-0.10	0.17	-0.43	0.23
Age0:LD:_2010s	-0.09	0.14	-0.37	0.18
Age1:LD:_1980s	-0.08	0.11	-0.30	0.13
Age1:LD:_1990s	-0.08	0.11	-0.30	0.13
Age1:LD:_2000s	-0.09	0.12	-0.32	0.14
Age1:LD:_2010s	-0.08	0.11	-0.30	0.13
Age2:LD:_1980s	-0.04	0.07	-0.17	0.10
Age2:LD:_1990s	-0.04	0.07	-0.18	0.10
Age2:LD:_2000s	-0.04	0.07	-0.18	0.11
Age2:LD:_2010s	-0.04	0.07	-0.18	0.10
Age0:LD^2:1980s	0.01	0.12	-0.24	0.25
Age0:LD^2:1990s	0.01	0.02	-0.04	0.05
Age0:LD^2:2000s	0.01	0.04	-0.07	0.08
Age0:LD^2:2010s	0.01	0.12	-0.22	0.23
Age1:LD^2:1980s	0.00	0.04	-0.08	0.09
Age1:LD^2:1990s	0.00	0.02	-0.03	0.04
Age1:LD^2:2000s	0.00	0.02	-0.03	0.04
Age1:LD^2:2010s	0.00	0.04	-0.08	0.09
Age2:LD^2:1980s	0.00	0.01	-0.02	0.02
Age2:LD^2:1990s	0.00	0.01	-0.02	0.02
Age2:LD^2:2000s	0.00	0.01	-0.02	0.02
Age2:LD^2:2010s	0.00	0.01	-0.02	0.02

Section S1.2 Adult survival models

We investigated whether adult survival differed between breeders and non-breeders, and whether it was related to the average lay date of their breeding attempts (for breeders only, per decade, if an individual only nests once then this value is used), thereby investigating

whether lay date may be a measure of individual quality. To analyse the relationship between survival and lay dates in specific decades, we created four dummy variables for each decade to avoid lay dates in the 1980s influencing survival in the 2010s. Since not all individuals nested in all decades, and Program Mark cannot accept NAs in the individual covariates, we assigned the mean value (i.e. 0 because the data is standardised) to those individuals that were missing data. Our approach for adults avoided including lay date as a time-varying individual covariate where a separate covariate would be needed for each year of the study. Our approach narrows the maximum number of lay date values for an individual to just four, but still captures differences that may emerge due to birds nesting on average earlier or later than the mean of the population during different phases of their life.

Section S1.2.1 Sex-specific survival

We also investigated whether survival varied between males and females, which proves challenging in oystercatchers because of their similarity biometrically and morphometrically (van de Pol et al. 2009). Over the years, a number of birds have been sexed via DNA (n = 717, 54% of dataset for individuals that bred at least once), and from these it was possible to sex the partner for monogamous nests (n =331). Few nests had more than one partner and in these instances the partners were not sexed. During the course of the study, observations had also been made of individuals during copulation, and over the course of several seasons the certainty of sex increased. We only considered individuals where the sex was certain (n = 62), and their partners sexed another 44 individuals. Finally, we combined the observation data with biometrics and performed a conditional inference tree (Hothorn et al. 2006) in the R package partykit (Hothorn and Zeileis 2015). Only individuals with a prediction probability of 95% were considered, and after combining the biometric sexing with observational data, another 127 individuals were sexed. The most important biometric identifiers for females were bill lengths greater than 76.1mm in combination with bill heights less than 10.8mm whilst males had bills less than 74.9mm and bill heights >10.5mm. From the individuals we sexed, bill length for females averaged 79.9mm (sd = 27.4mm) and for males 70.0mm (sd = 24..1mm) whilst bill height for females averaged 10.3mm (sd = 3.3mm) and for males 10.7mm (sd = 4.3mm). Finally, partners of individuals sexed through the combination of observational and biometric data could be sexed (n = 22). The remaining 25 individuals had an uncertain biometry and too few observations and could thus not be sexed. The multi-step process of sexing individuals meant that several checks could be performed, for example same-sex partnerships in which individuals were sexed through different methods. We only detected one individual where this occurred and although some errors may be present, we believe this to be a minority.

Section S1.2.2 Adult survival results

Only a single model was supported (i.e. ΔAIC <10) which included breeding state in interaction with decade (Table S4). The alternative structures for resighting, transition and dead recovery are displayed in Table S5, where again only a single model was supported (i.e. ΔAIC <10). Survival of breeders was relatively constant through the decades, albeit with a drop between the 1990s and 2000s, with average survival in the 1980s of 93.4%, 1990s of

93.7%, a drop in the 2000s to 91.8% and remaining lower in the 2010s at 91.7% (Figure S3). Non-breeder survival was however more variable with higher survival in the 1980s and 2000s (90.6% and 91.0% respectively) and lower survival in the 1990s and 2010s (86.1% and 85.8% respectively; Figure S3). The lack of support for models with lay date meant there was no contribution to model averaging and the coefficients were thus zero (Table S6; Figure S3). Our approach of using average lay dates for an individual relies on accurate estimation of first clutch lay dates, and hence a lack of a lay date effect could potentially be the result of inaccurate lay date estimates. However, the majority of nests were found during the egg-laying phase, suggesting a high detection probability and that clutches were accurately assigned as first clutches rather than replacement clutches. Unfortunately trying to resolve issues where replacement clutches were identified as first clutches post-hoc would be difficult given that large inter-annual changes in lay date are plausible given local environmental and/or individual effects. We are however confident that this issue affects a minority of the data.

Including sex did not improve model fit (ΔAICc = 1.17). Sex-specific variation in survival of breeders was low among decades and the overall average for the study period was similar (M = 0.929, F = 0.926; Figure S4). Annual estimates of survival for males and females is shown in Appendix S4 where it can be seen that survival varied among years, and in some years males had higher survival but in other years females had higher survival, but once averaging survival over decades the survival rates become similar (Figure S4). The similarity in survival, and lack of clear support for sex-specific survival means that differences were further reduced after model-averaging and the only apparent differences are in non-breeding survival (Figure S4). There was also less overlap of non-breeder survival estimates between the models that included or excluded sex, however, a large proportion of birds that that never bred on the island were never sexed meaning the sample-sizes of sex-specific non-breeding survival were substantially lower (Figure S4). The standard errors were consequently also larger. The potential influence of sex-specific survival estimates of breeders and non-breeders on population growth rates were further explored using sex-specific IPMs, the results of which are described in Appendix S6.

Table S4 – Results of the model comparison for the adult survival parameters. All models contained the same structure for other parameters, namely resighting probability = stratum + stratum:time, dead recovery = stratum + stratum:time, transitions = stratum:tostratum + stratum:tostratum:time. Nr is the model number, Model is the structure of the survival parameters, npar is the number of parameters, ΔAICc is delta AIC and the top model had an AICc of 26151.1. Stratum is the breeding state (breeders, non-breeders and pre-breeders), Dec is decade and LD is lay date.

Nr	Model	npar	ΔAICc	w_i
1	~stratum * Decade	313	0.00	0.64
2	~stratum * Sex * Decade†	321	1.17	0.36
3	~stratum	306	14.48	0.00
4	~stratum * Decade +LD1980s + LD1990s + LD2000s + LD2010s	339	43.41	0.00
5	~Decade	310	59.50	0.00

6	~stratum * Decade + LD1980s + LD1990s + LD2000s + LD2010s + LD1980^2 + LD1990^2 + LD2000^2 + LD2010^2	357	65.65	0.00
7	~1	306	128.24	0.00

† Note that sample sizes differed in the model that included Sex, as only individuals with known sex were included. The ΔAICc and $\boldsymbol{w_i}$ of model 1 and 2 thus refers to models that only included sexed individuals (Table S1), but given the lack of support for sex-specific survival, the full dataset including unsexed individuals was used to estimate survival.

Table S5 - Results of model selection for the resighting, transition and dead recovery model structure. Here we present all models that included the top survival structure (i.e. stratum*Decade) along with models containing a null survival structure (i.e. intercept only survival). Str is the breeding status, Dec is decade, time is a factorial variable with a parameter estimated for each year whilst Time is a continuous estimates with a single parameter estimated, Null is an intercept only model. ΔAICc is the difference in AIC to the top performing model, which had an AIC of 26151.1

Rank	Survival	Resighting	Transition	DeadRec	npar	ΔAICc
1	Str * Dec	Str + Str:time	Str:toStr + Str:toStr:time	Str + Str:time	313	0.00
2	Str * Dec	Str + Str:time	Str:toStr + Str:toStr:time	time	241	41.42
3	Str * Dec	Str + Str:time	Str:toStr + Str:toStr:time	Str	208	54.23
4	Str * Dec	Str + Str:time	Str:toStr + Str:toStr:time	Null	206	58.24
5	Null	Str + Str:time	Str:toStr + Str:toStr:time	Str + Str:time	306	128.24
6	Null	Str + Str:time	Str:toStr + Str:toStr:time	time	234	174.30
8	Str * Dec	Str + Str:time	Str:toStr + Str:toStr:Time	Str + Str:time	181	234.41
9	Str * Dec	Str + Str:time	Str:toStr	Str + Str:time	177	240.02
7	Null	Str + Str:time	Str:toStr + Str:toStr:time	Str	201	256.28
10	Null	Str + Str:time	Str:toStr + Str:toStr:Time	Str + Str:time	174	299.29
11	Null	Str + Str:time	Str:toStr	Str + Str:time	170	337.00
12	Null	Str + Str:time	Str:toStr + Str:toStr:time	Null	199	522.47
13	Str * Dec	Str + Str:time	Null	Str + Str:time	174	577.80
14	Null	Str + Str:time	Null	Str + Str:time	167	635.40
15	Str * Dec	Str	Str:toStr + Str:toStr:time	Str + Str:time	223	1228.83
16	Null	Str	Str:toStr + Str:toStr:time	Str + Str:time	216	1354.83
17	Str * Dec	time	Str:toStr + Str:toStr:time	Str + Str:time	255	2752.98
18	Null	time	Str:toStr + Str:toStr:time	Str + Str:time	248	2917.30
19	Str * Dec	Null	Str:toStr + Str:toStr:time	Str + Str:time	221	3406.63
20	Null	Null	Str:toStr + Str:toStr:time	Str + Str:time	183	3435.88

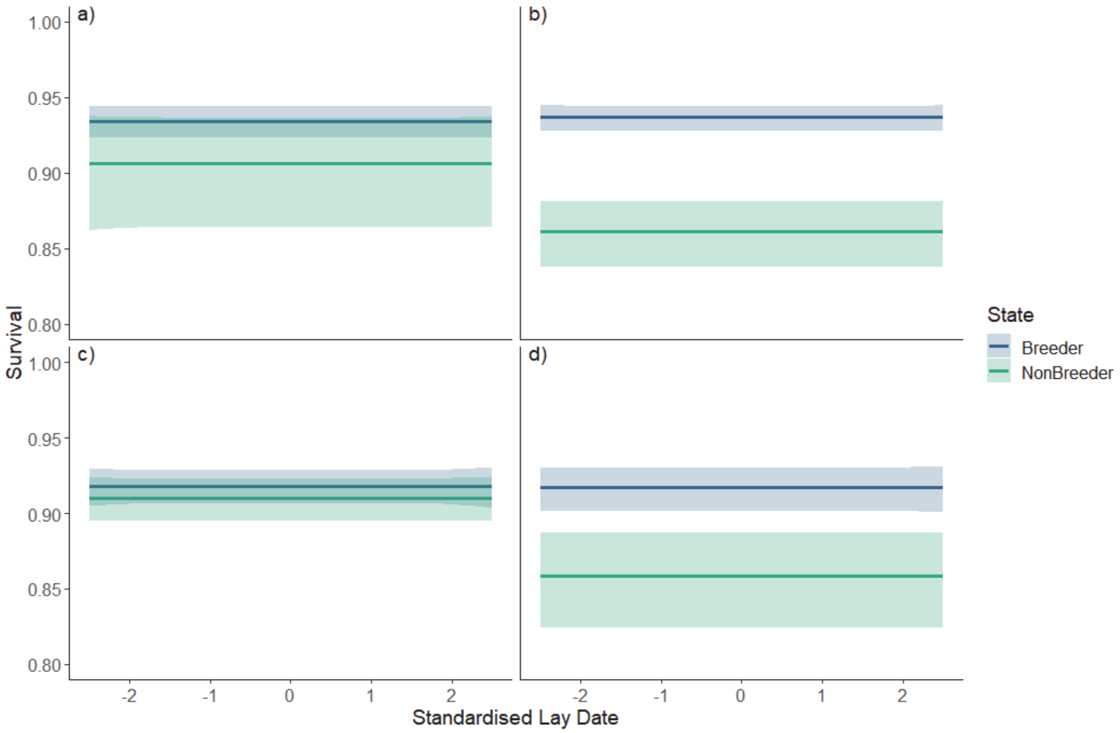

Figure S3 – Predicted adult survival of breeders and non-breeders in relation to (standardised) lay date (solid line) of breeding attempts for a) 1980s, b) 1990s, c) 2000s and d) 2010s. Coefficients were averaged from all considered adult survival models and weighted by the Akaike weight.

Table S6 – Model-averaged beta coefficients for models considered for adult survival. Parameters were weighted according to the Akaike weights shown in Table S4. The intercept is adult survival of breeders in the 1980s, NB is non-breeders, se is the standard error, lcl and ucl are the 95% lower and upper confidence limits. Note that sex-specific parameters are shown in Appendix S6: Table S1.

Parameter	Coefficient	se	lcl	ucl
Intercept	2.65	0.09	2.49	2.82
1990s	0.04	0.11	-0.18	0.26
2000s	-0.24	0.11	-0.46	-0.02
2010s	-0.25	0.13	-0.50	-0.00
NB	-0.38	0.23	-0.84	0.08
NB 1990s	-0.49	0.27	-1.01	0.03
NB 2000s	0.28	0.26	-0.24	0.80
NB 2010s	-0.22	0.29	-0.78	0.35
LD	0.00	0.00	0.00	0.00
LD^2	0.00	0.00	0.00	0.00

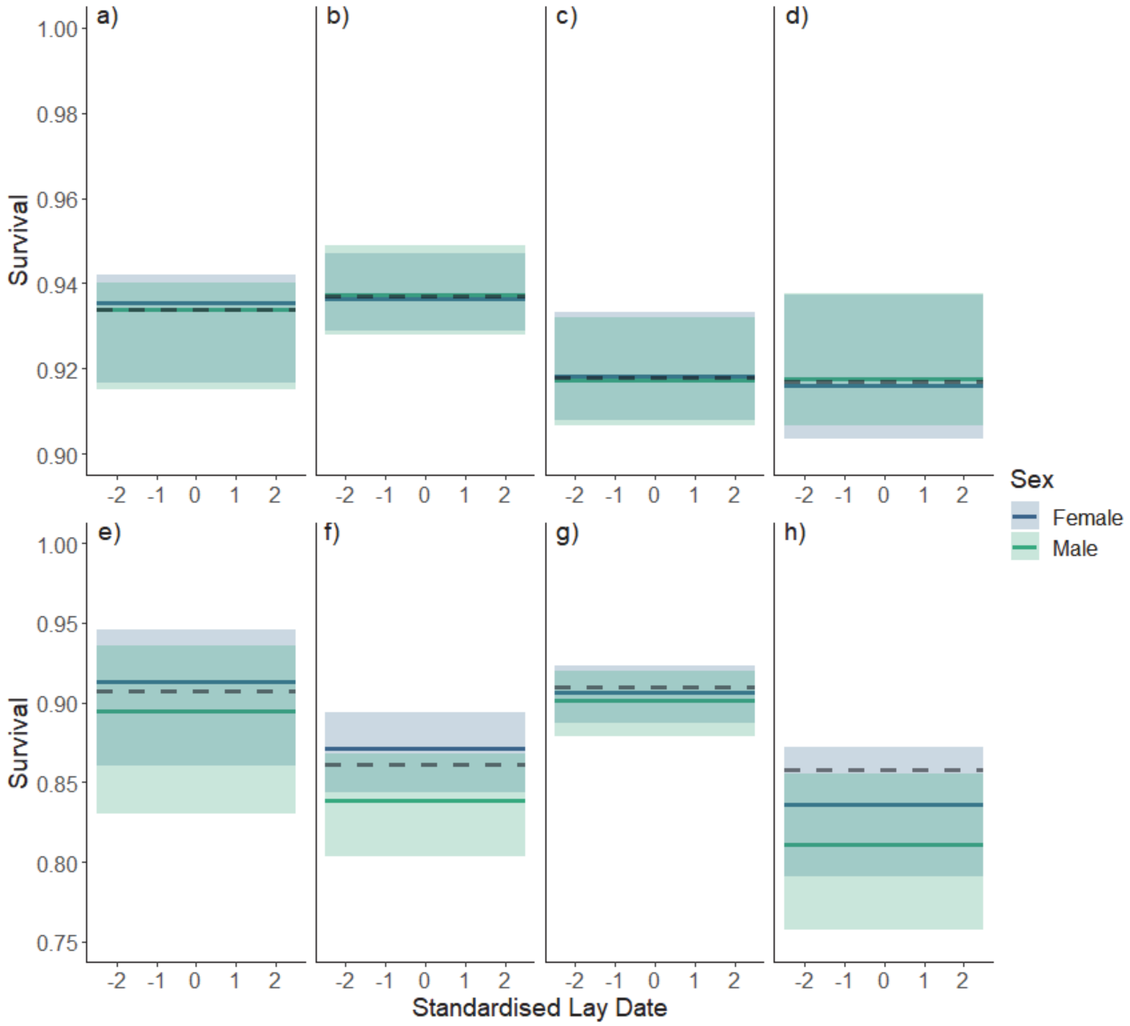

Figure S4 – Model-averaged survival estimates per sex and breeding state for breeders (a – d) and non-breeders (e – h) in the 1980s (a, e), 1990s (b, f), 2000s (c, g) and 2010s (d, h). The survival estimates were model-averaged according to the Akaike weights shown in Table S4. The dashed line is the survival estimate from of model 1 in Table S4, i.e. the estimates used in the IPM. The shaded area is the 95% confidence limits. Note that the sample size of model 2 was different as only individuals of known sex were included (Table S1).

SECTION S2 - GROWTH G(Z', Z)

Growth (or more generally: the distribution of state variable values next year) here involves changes in age, breeding status and lay date.

Section S2.1 Age

No model was needed for age, as age advanced by one each year.

Section S2.2 Breeding Status

We investigated alternative model options for next year's breeding probability, including an intercept model and whether breeding probability depended upon age, current breeding status or both. Only one individual was detected during the study period (1983 - 2018) breeding at an age of two. Therefore, we removed this observation and set a minimum breeding age of three. In total there were 2,794 observations of 702 individuals where the breeding status was known. We considered models that include a linear effect of age, or a quadratic relationship with age where breeding probability initially increases with age before later declining as individuals experience senescence. These variables were included separately and as an interaction effect. As per other analyses, we also considered how breeding probability may vary across the four decades of our study period. Due to the low number of older individuals in the 1980s (note, to know the age of an oystercatcher, it had to be ringed as a chick or sub-adult), we also considered a categorical variable where the 1980s and 1990s were combined, thus three levels of pre2000s, 2000s and 2010s instead of the four decades. We also considered how breeding probability may vary between males and females. This consisted of a smaller dataset (2372 observations of 409 individuals) where individuals had been previously sexed (see details of sexing in section *1.2.1*). We considered the same set of models as above except we included an interaction with Sex.

Section S2.2.1 Breeding status results

The top performing model based on AIC included an interaction between decade and current breeding state, an interaction between current breeding state and age and an interaction between current breeding state and age-squared (Table S7). No other model was statistically supported and hence the model-averaged parameters are only based on this model (weights w_i are shown in Table S7). Nonbreeders had a lower probability of breeding next year than breeders, across all life stages (Table S8, Figure S5). Breeding probability had a significant quadratic relationship with age with young individuals (<7) and old individuals (>25) having a lower probability of breeding (Table S8, Figure S5). In all cases a model with a simplified structure for decade outperformed the model containing all four decades (ΔAIC > 4). Breeding probabilities during the last decade (2010s) were significantly higher than the 2000s (intercept year) or the 1980s and 1990s (i.e. pre2000s; Table S8). The model containing Sex did not perform better than a model that excluded sex (ΔAIC = 0.53). Visual inspection of the results indicated that most differences were in the latter age classes, along with minor differences for middle-aged non-breeders in the Pre2000s (Figure S6). The potential influence of sex-specific differences in breeding probability on population growth rates are described in Appendix S6.

Table S7 – Results of model selection for next year's breeding probability. AIC is the Akaike information criterion, df is degrees of freedom, BS is current breeding status. In all cases, the simplified variable of decade containing only three classes (Pre2000s, 2000s, 2010s) outperformed the variable containing four classes, thus the results of the latter are excluded.

Model	df	AIC	ΔAIC	w_i
Decade*BS + BS*Age + BS*Age^2	10	726.70	0	1
BS*Age + BS*Age^2	6	750.79	24.09	0

Decade*BS + BS*Age	8	913.19	186.48	0
Decade + BS	4	920.60	193.89	0
BS	2	938.84	212.14	0
Decade*Age + Decade*Age^2	9	946.33	219.62	0
Age + Age^2	3	1008.55	281.85	0
Age	2	1435.17	708.47	0
Decade	3	1747.28	1020.57	0
NULL	1	2177.34	1450.64	0

Table S8 – Model-averaged coefficients, of models shown in Table S7, for next-year's breeding probability. P-values shown in bold are significant (i.e. P<0.05). The intercept is Breeder in 2000s. Note that sex-specific parameters are shown in Appendix S6: Table S2.

Parameter	Estimate	SE	P-value
(Intercept)	-0.887	0.763	0.245
NonBreeder	-4.080	0.922	**<0.001**
2010s	1.209	0.478	**0.011**
Pre2000s	0.097	0.442	0.827
Age	0.646	0.098	**<0.001**
Age^2	-0.020	0.003	**<0.001**
NonBreeder:2010s	0.395	0.664	0.552
NonBreeder:Pre2000s	-0.490	0.537	0.362
NonBreeder:Age	0.342	0.144	**0.018**
NonBreeder:Age^2	-0.017	0.005	**0.001**
Age:2010s	0.000	0.000	1.000
Age:Pre2000s	0.000	0.000	1.000
Age^2:2010s	0.000	0.000	1.000
Age^2:Pre2000s	0.000	0.000	1.000

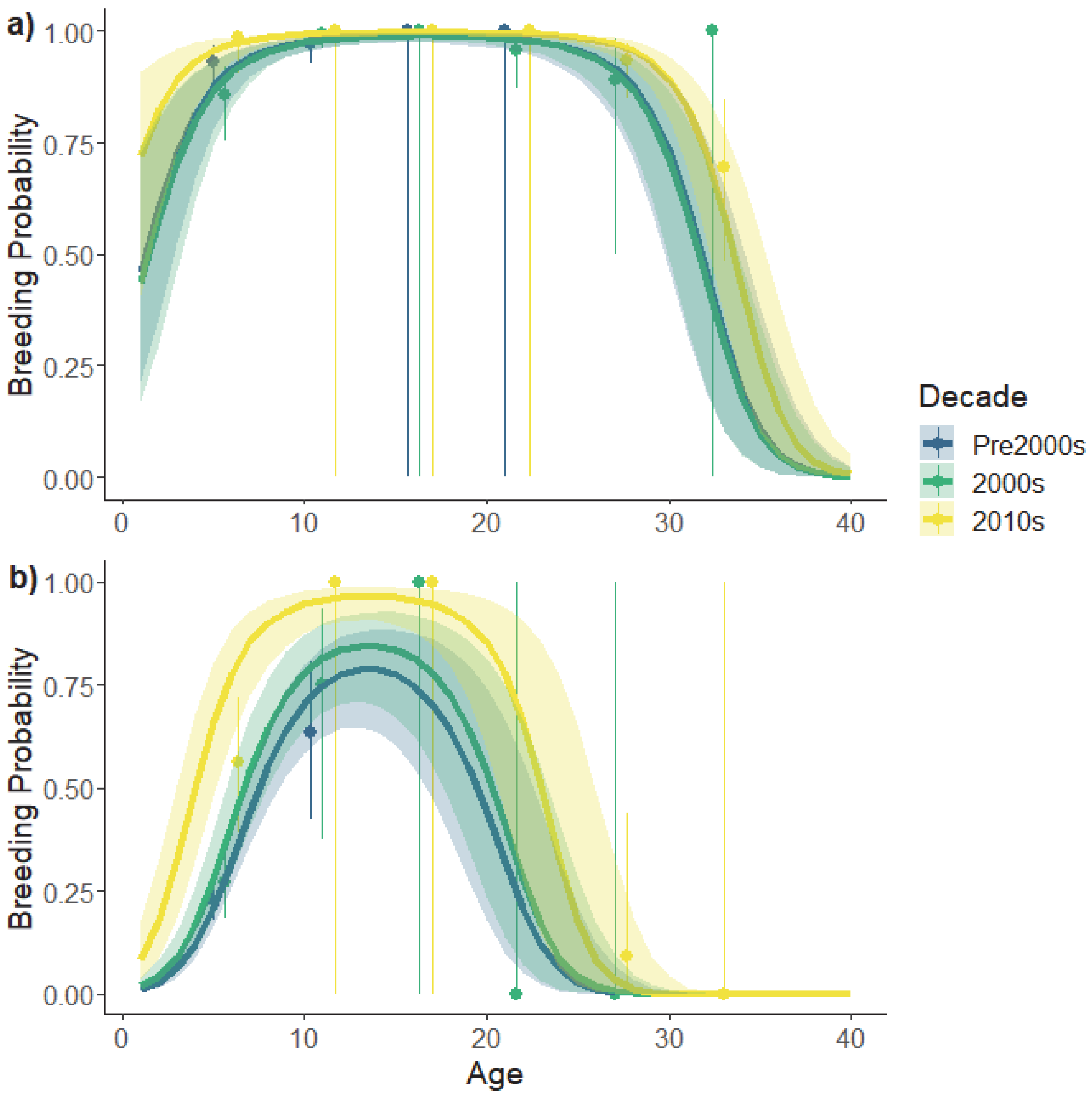

Figure S5 – Predictions and 95% confidence intervals, based on model-averaged coefficients, for breeding probability of a) Breeders and b) Non-breeders. Predictions are across three time periods (pre2000s = 1980s and 1990s) and forty age classes. The points are the average breeding probability across ten bins of age class, and the vertical lines represent standard deviations. The points and error bars were jittered to avoid overlap. The values for the binned ages were estimated through a separate glm that contained categorical values for each age bin.

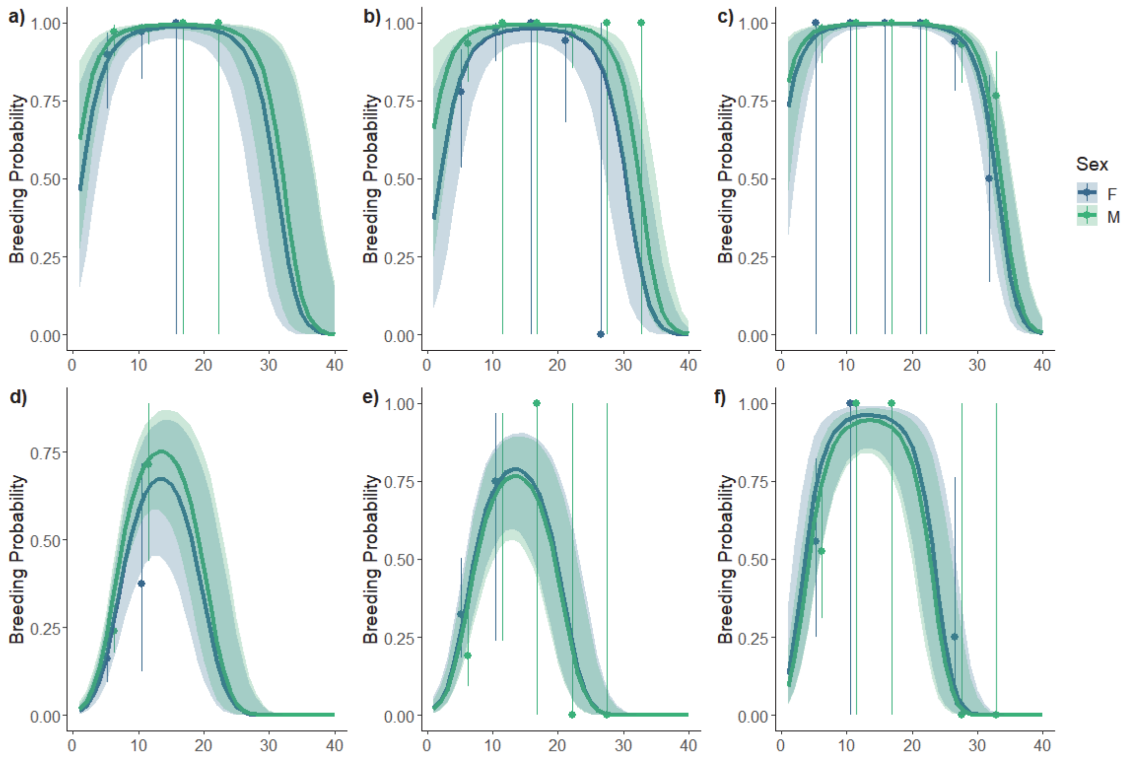

Figure S6 – Sex-specific predictions and 95% confidence intervals, based on model-averaged coefficients, for breeding probability of Breeders (a,b,c) and Non-breeders (d,e,f) for the 1980s and 1990s combined (a,d), 2000s (b,e) and 2010s (c,f) in relation to age. The points are the average breeding probability across ten bins of age class, and the vertical lines represent standard deviations. The points and error bars were jittered to avoid overlap. The values for the binned ages were estimated through a separate glm that contained categorical values for each age bin.

Section S2.3 Lay date

Since not all individuals nested every year, or were not detected each year, we considered two model structures whereby we only considered consecutive lay dates, i.e. lay dates at time t and $t + 1$, or an alternative structure for lay dates at time t and $t + n$ to increase sample size and detect growth for individuals that skip breeding in some years. However, model results were comparable between the two methods, and we used estimates of the former model based on birds that nested in consecutive years (541 nests and 97 individuals). The structure of our IPM meant that lay date could only advance for breeders, whilst non-breeders would keep their most recent lay date until they resumed breeding. Therefore, the state variable of breeding status was not considered in our lay date model. We did however consider a model structure with age to determine if the development of lay date depended on the age of an individual. The models were fitted using generalised least squares to allow for unequal variances, using the gls function in R (Pinheiro et al. 2020). We considered alternative variance structures and chose the best performing model based on differences in AIC and by visually inspecting the fitted models.

Section S2.3.1 Lay date results

An exponential variance structure that contained lay date provided the best model fit, and hence all presented models contain this variance structure (Table S9). The variance function coefficient was 0.0706 and thus took the form $\exp(2\theta v)$ where v is the variance covariate (lay date) and θ is the variance function coefficient. The top performing model only included lay date (Table S9; Figure S7), which was significantly related to lay date in the next year (FigureA3; Table S10). The model of Age + LayDate provided little statistical support despite Age having a significant relationship with next year's lay date (P = .045), but the low Akaike weight meant that Age had a negligible effect on the model-averaged coefficients used in the IPM (Table S9; Table S10).

Table S9 - Results of model selection for next-year's lay date. AIC is the Akaike information criterion, df is degrees of freedom.

Model	Df	AIC	ΔAICc	w_i
LayDate	3	1249.18	0	0.93
LayDate + LayDate^2	4	1256.15	6.96	0.03
Age + LayDate	5	1256.32	7.14	0.03
Age + LayDate + LayDate^2	5	1263.25	14.06	0
Age + Age^2 + LayDate	5	1270.84	21.65	0
Age + Age^2 + LayDate + LayDate^2	6	1277.76	28.58	0
NULL	1	1360.65	111.47	0
Age	3	1360.71	111.53	0
Age + Age^2	4	1375.04	125.86	0

Table S10 – Model-average coefficients, of models shown in Table S9, for next-year's lay date. P-values in bold are significant (i.e. P < 0.05)

Parameter	Coefficient	SE	P-value
Intercept	-0.055	0.044	0.211
LayDate	0.451	0.039	**<0.001**
LayDate^2	-0.000	0.006	0.962
Age	0.000	0.002	0.883
Age^2	0.000	0.000	0.999

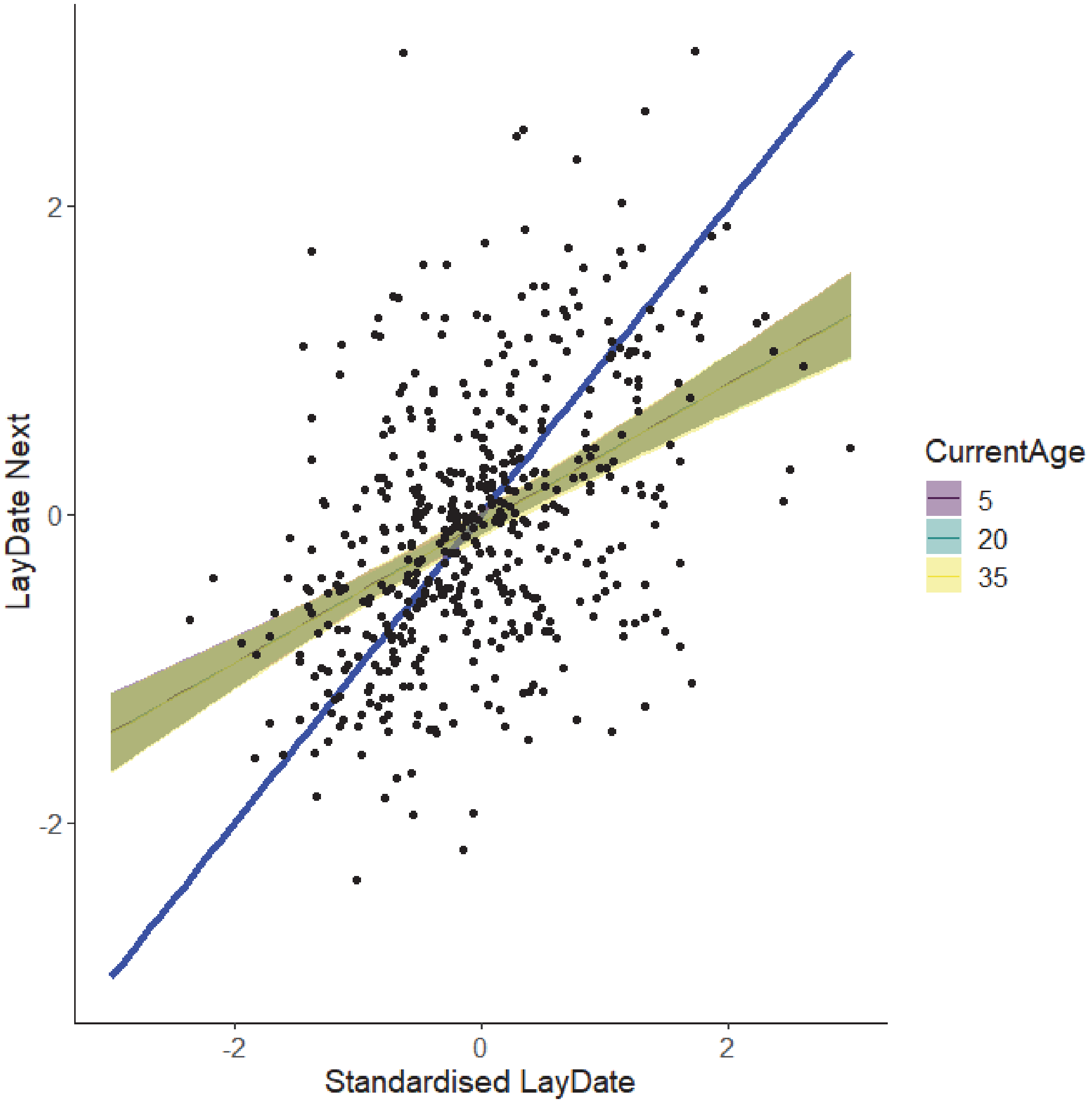

Figure S7 – Predictions and 95% confidence intervals, based on model-averaged coefficients, for lay date in the next year as a function of the current lay date and age. Model predictions are shown for ages 5, 20 and 35 and the shaded areas show the 95% confidence intervals for the model predictions. Age had a negligible effect and hence the model predictions largely overlap. Black points are the nesting data of oystercatchers which nested in consecutive years (n = 541) and the solid blue line represents a 1:1 line (i.e. if oystercatchers were to keep the same lay date year on year).

SECTION S3 - REPRODUCTION R(Z)

As described in the main text, reproduction consisted of several phases including nest success, number of hatchlings, hatchling survival, fledgling survival, probability of a replacement clutch and the vital rates for the replacement clutch. Nest searches were conducted between the end of April and the end of July, and territories were visited every other day to either search for new nests or check existing nests for new eggs (van de Pol et al.

2006). The number of nests monitored annually exceeded 100 nests across the study period (Appendix S4: Figure S1). The reproduction protocol in the study area is described in van de Pol et al. (2006). For each vital rate, we considered alternative models for vital rate regressions. Of the three state variables, we only consider lay date for the reproduction vital rate regressions. Only breeders can reproduce, and the study setup presented challenges in considering age. Individuals less than three do not reproduce and may not initiate breeding until age five or six (Figure S5; Ens et al. 2014). Furthermore, age is only known for individuals ringed as chicks meaning that "old" individuals (e.g. >25) are not present in the study until the late 2000s, when reproduction had declined (Roodbergen et al. 2012), and thus it may be difficult to separate age effects from environmental effects – whilst decadal comparisons would be impossible. We therefore only investigate the relationship between reproduction vital rates and lay date, together with a categorical variable of decade. We considered models with and without an interaction between decade and lay date, and with a quadratic term for lay date. Regressions were not performed for fledgling survival, and instead this was analysed using a mark-recapture analysis (see *Fledgling and Sub-adult Survival* above).

Section S3.1 Nest Success

The top model included an interaction between decade and a quadratic relationship with lay date (Table S11). In all decades the relationship between nest success and lay date was parabolic, with nest success generally reaching its maximum before the mean lay date (Figure S8). Nest success, and its relationship with lay date varied across decades and the 2000s and 2010s had significantly lower nest success than the intercept of 1980s (Table S12; Figure S8). In all decades, the parabolic relationship is present although the shape may vary, for example in the 2010s the success of early lay dates was more similar to that of mean lay dates, whereas in the 2000s early lay dates had a much lower success than average lay dates (Figure S8). Average nest success was 0.53 in both the 1980s (±0.02) and 1990s (±0.02), 0.31 in the 2000s (±0.02) and 0.39 in the 2010s (±0.02).

Table S11 – Results of model selection for nest success. df is degrees of freedom, AICc is the Akaike Information Criterion and w_i is the Akaike weight.

Model	df	AICc	ΔAICc	w_i
Decade*LayDate + Decade* LayDate^2	12	6361.46	0	1
Decade*LayDate	8	6432.89	71.43	0
LayDate + LayDate^2	3	6544.14	182.69	0
LayDate	2	6601.73	240.27	0
Decade	4	6627.42	265.96	0
Null	1	6790.44	428.98	0

Table S12 – Model-averaged coefficients, of models shown in Table S11, for nest success. Models were weighted by Akaike weights. P-values in bold are significant (i.e. P < 0.05)

Parameter	Coefficient	SE	P-value
Intercept	0.349	0.086	**<0.001**
LayDate	-0.346	0.073	**<0.001**

LayDate^2	-0.204	0.056	**<0.001**
1990s	-0.033	0.105	0.750
2000s	-0.901	0.121	**<0.001**
2010s	-0.731	0.117	**<0.001**
LayDate:1990s	0.007	0.090	0.942
LayDate:2000s	-0.065	0.111	0.558
LayDate:2010s	-0.217	0.114	0.056
1990s:LayDate^2	-0.024	0.069	0.730
2000s:LayDate^2	-0.134	0.097	0.167
2010s:LayDate^2	0.001	0.095	0.990

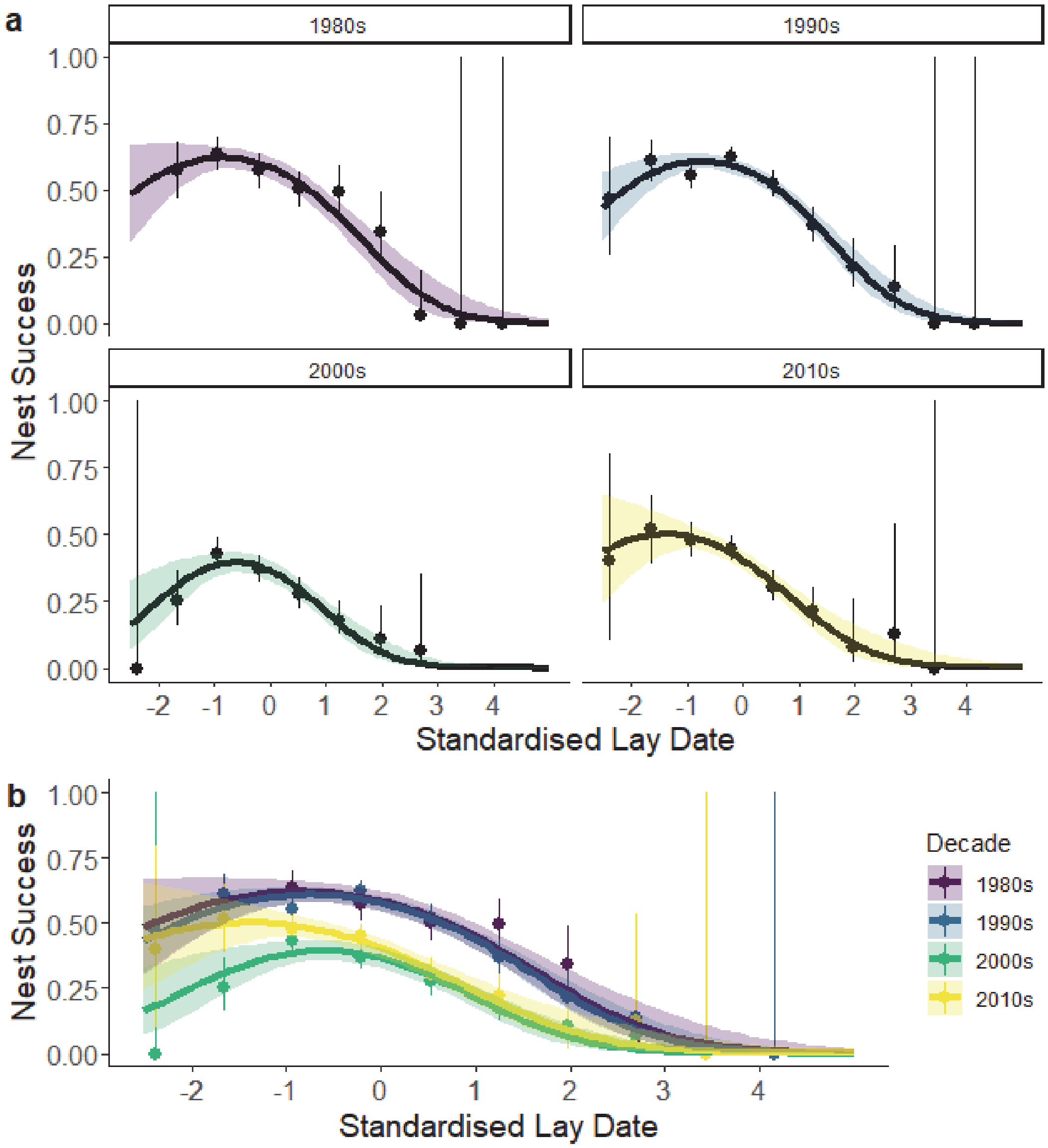

Figure S8 – Predictions and 95% confidence interval (shaded area) of model-averaged coefficients of nest success in relation to lay date across the four decades of the study period. a) shows the predictions for each decade separately and b) aids comparison across decades by overlaying these. The points are the average nest success across ten bins of lay date, and the vertical lines represent standard deviations. Sample sizes were smaller for very early and very late lay dates. The values for the binned lay dates were estimated through a separate glm that contained categorical values for each lay date bin.

Section S3.2 Number of hatchlings

The number of hatchlings were only estimated for nests that were successful. We therefore used a zero-truncated poisson model. Models that included LayDate had strong statistical support but there was also some statistical support for differences among decades (i.e. ΔAICc <4; Table S13). The number of hatchlings declined significantly with later lay dates (Table

S14; Figure S9). The average number of hatchlings were 2.4 in the 1980s (± 0.04), 2.3 in the 1990s (± 0.05) and 2010s (± 0.06), and 2.1 in the 2000s (± 0.06).

Table S13 – Results of model selection for the number of hatchlings from successful nests. df is degrees of freedom, AICc is the Akaike Information Criterion and w_i is the Akaike weight.

Model	df	AICc	**ΔAICc**	w_i
LayDate + LayDate^2	3	6235.96	0	0.46
LayDate	2	6236.79	0.82	0.31
Decade*LayDate	8	6238.10	2.14	0.16
Decade*LayDate + Decade*LayDate^2	12	6239.61	3.65	0.07
Decade	4	6248.26	12.30	0.00
Null	1	6251.80	15.84	0.00

Table S14 – Model-averaged coefficients, of models shown in Table S13, for number of hatchlings. Models were weighted by Akaike weights. P-values in bold are significant (i.e. P < 0.05)

Parameter	Coefficient	SE	P-value
(Intercept)	0.689	0.028	**<0.001**
LayDate	-0.096	0.022	**<0.001**
LayDate^2	-0.014	0.020	0.500
1990s	-0.009	0.025	0.723
2000s	-0.035	0.069	0.612
2010s	-0.014	0.034	0.687
1990s:LayDate	0.009	0.025	0.710
2000s:LayDate	0.005	0.029	0.853
2010s:LayDate	-0.001	0.027	0.958
1990s:LayDate^2	-0.004	0.016	0.815
2000s:LayDate^2	-0.008	0.032	0.805
2010s:LayDate^2	-0.006	0.026	0.807

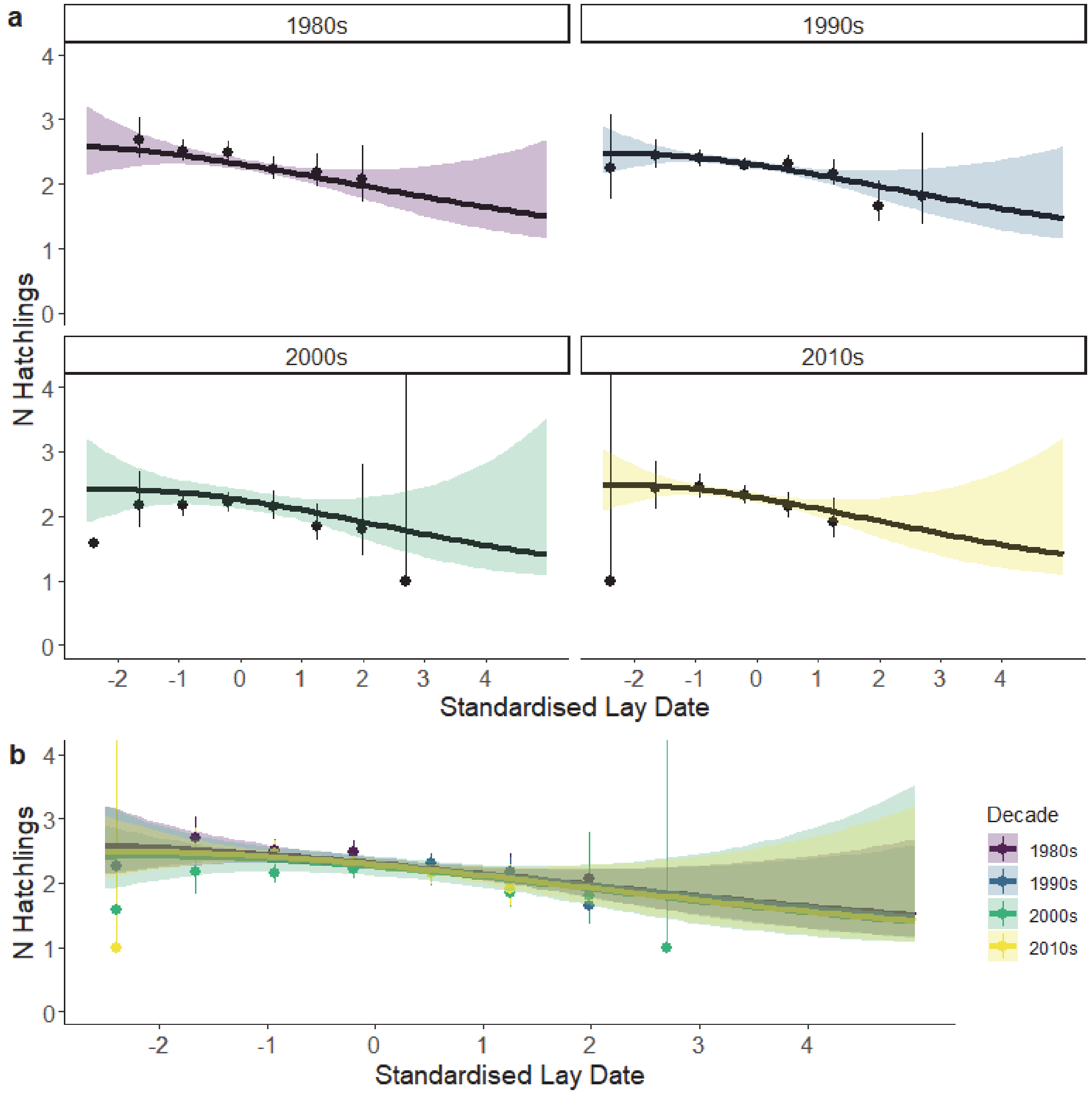

Figure S9 – Predictions and 95% confidence interval (shaded area) of model-averaged coefficients for the number of hatchlings in relation to decade and lay date. a) shows the predictions for the four decades and b) overlays these to aid comparison. The points are the average number of hatchlings across ten bins of lay date, and the vertical lines represent standard deviations. Sample sizes were smaller for very early and very late lay dates. The values for the binned lay dates were estimated through a separate glm that contained categorical values for each lay date bin.

Section S3.3 Hatchling Survival

We analysed the survival of individual hatchlings up to fledging with binomial regression models. Two models performed similarly in explaining hatchling survival which included an interaction between decade and lay date, and with lay date squared (Table S15). Hatchling survival was significantly lower in the 1990s and 2000s. Interestingly, hatchling survival appeared to increase, rather than decrease, as lay date increased (Figure S10; Table S16). However, this pattern is not present for nests later than two standard deviations from the

mean, where hatching survival appeared to be zero (Figure S10). Average hatchling survival was highest in the 1980s (0.25 ±0.01), lowest in the 2000s (0.12 ±0.01) and similar in the 1990s (0.22 ±0.01) and 2010s (0.22 ±0.02).

Table S15 – Results of model selection for hatchling survival. df is degrees of freedom, AICc is the Akaike Information Criterion and w_i is the Akaike weight.

Model	df	AICc	**ΔAICc**	w_i
Decade*LayDate	8	4055.09	0	0.63
Decade*LayDate + Decade*LayDate^2	12	4056.44	1.35	0.32
Decade	4	4060.40	5.31	0.04
LayDate	2	4109.50	54.42	0
LayDate + LayDate^2	3	4111.51	56.42	0
Null	1	4112.76	57.67	0

Table S16 – Model-averaged coefficients, of models listed in Table S15, for hatchling survival. Models were weighted by Akaike weights. P-values in bold are significant (i.e. $P < 0.05$)

Parameter	Coefficient	SE	P-value
Intercept	-1.044	0.098	**<0.001**
LayDate	-0.060	0.081	0.456
LayDate^2	-0.058	0.095	0.539
1990s	-0.279	0.120	**0.020**
2000s	-0.931	0.167	**<0.001**
2010s	-0.251	0.132	0.058
1990s:LayDate	-0.113	0.106	0.284
2000s:LayDate	0.315	0.181	0.082
2010s:LayDate	-0.056	0.139	0.688
1990s:LayDate^2	0.069	0.113	0.541
2000s:LayDate^2	0.077	0.145	0.596
2010s:LayDate^2	0.054	0.108	0.615

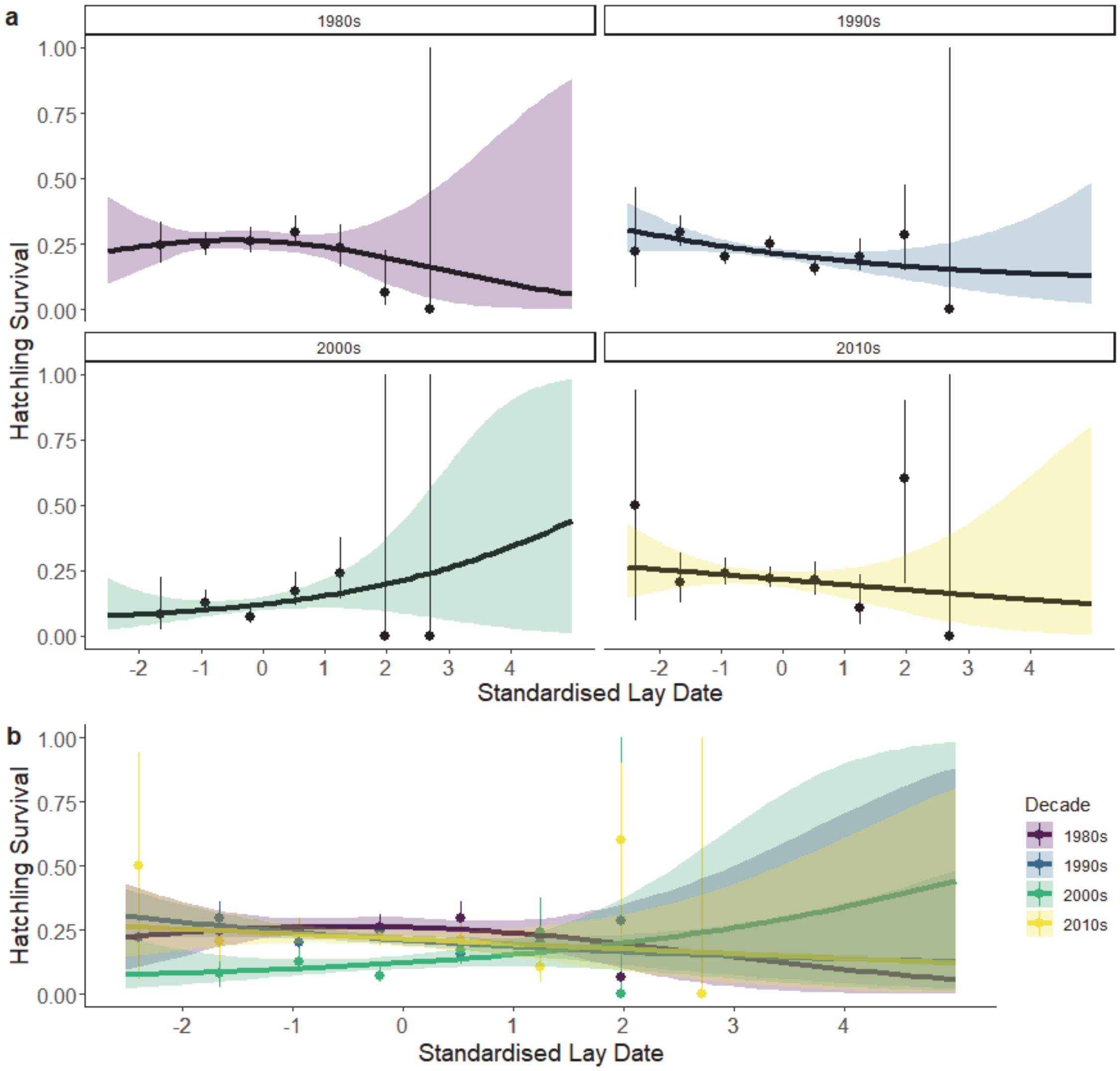

Figure S10 – Predictions and 95% confidence interval (shaded area) of model-averaged coefficients for hatchling survival (up to fledging) in relation to decade and lay date. a) shows the predictions for the four decades and b) overlays these to aid comparison. The points are the average hatchling survival across ten bins of lay date, and the vertical lines represent standard deviations. Sample sizes were smaller for very early and very late lay dates. The values for the binned lay dates were estimated through a separate glm that contained categorical values for each lay date bin.

Section S3.4 Replacement Clutch Probability

If their first nest fails, oystercatchers may or may not attempt a replacement clutch. Here we analyse that probability. The top performing model based on AIC included an interaction between decade and a quadratic relationship with lay date (Table S17). No other models were within ($\Delta AIC < 4$). The probability of a replacement clutch decreased significantly with later

lay dates (Figure S11; Table S18) and were similar for the 1980s, 1990s and 2010s but were significantly lower in the 2000s (Figure S11; Table S18)

Table S17 – Results of model selection for the probability of a replacement clutch if the first fails. df is degrees of freedom, AICc is the Akaike Information Criterion and w_i is the Akaike weight.

Model	df	AICc	ΔAICc	w_i
Decade*LayDate + Decade*LayDate^2	12	2895.08	0	0.92
Decade*LayDate	8	2899.84	4.76	0.08
LayDate + LayDate^2	3	2934.84	39.76	0
LayDate	2	2944.16	49.08	0
Decade	4	3178.90	283.82	0
Null	1	3220.61	325.53	0

Table S18 – Model-averaged coefficients, of models listed in Table S17, for the probability of a replacement clutch. Models were weighted by Akaike weights. P-values in bold are significant (i.e. $P < 0.05$)

Parameter	Coefficient	SE	P-value
(Intercept)	-0.566	0.160	**<0.001**
LayDate	-0.884	0.141	**<0.001**
LayDate^2	-0.285	0.155	0.066
1990s	-0.231	0.181	0.201
2000s	-0.784	0.191	**<0.001**
2010s	-0.043	0.190	0.819
1990s:LayDate	0.130	0.167	0.438
2000s:LayDate	0.145	0.186	0.436
2010s:LayDate	0.028	0.180	0.878
1990s:LayDate^2	0.189	0.156	0.225
2000s:LayDate^2	0.115	0.165	0.486
2010s:LayDate^2	0.278	0.178	0.119

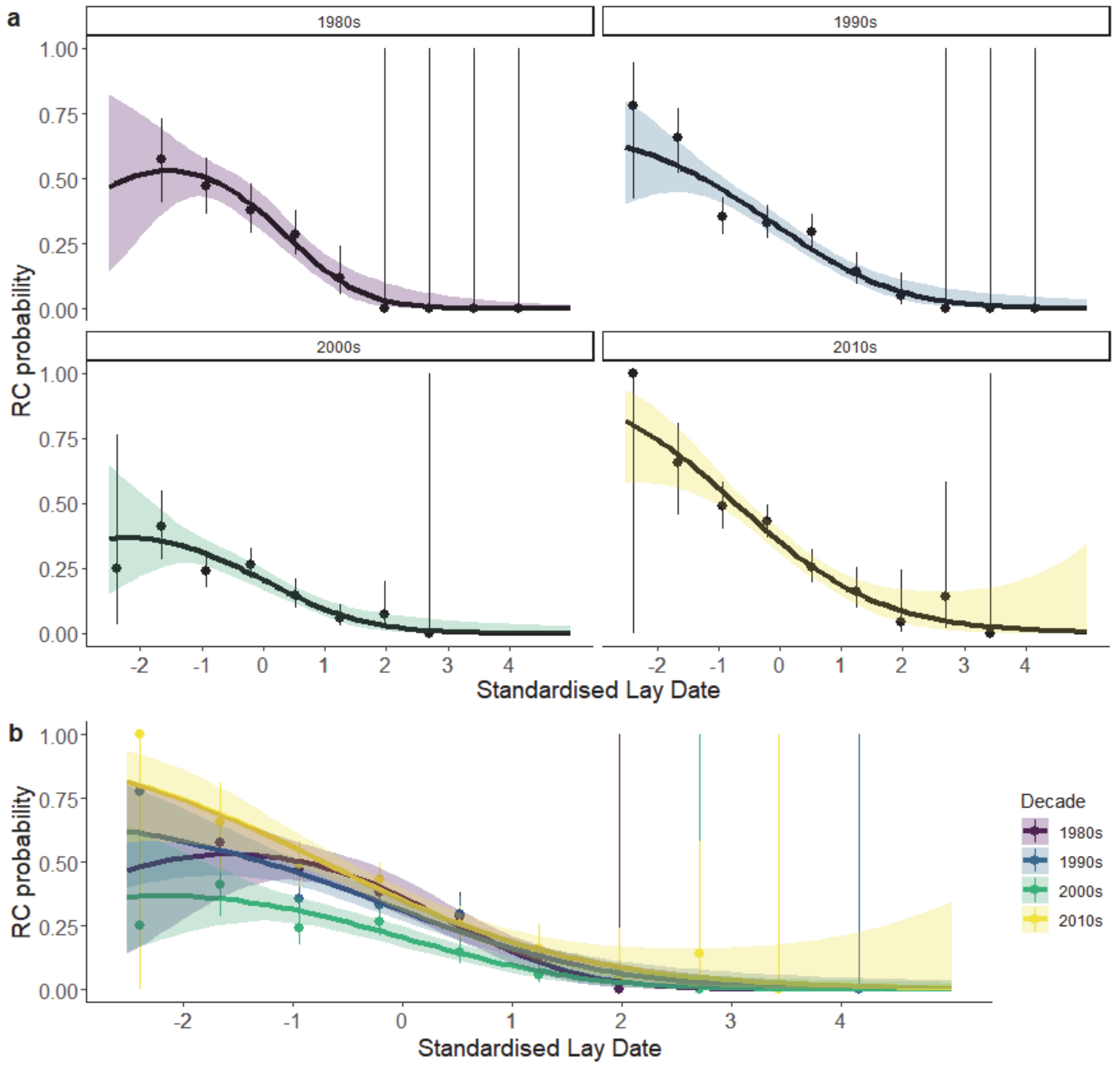

Figure S11 – Predictions and 95% confidence interval (shaded area) of model-averaged coefficients for probability of a replacement clutch (RC) decade and lay date. a) shows the predictions for the four decades separately and b) overlays these to aid comparisons. The points are the average replacement clutch probability across ten bins of lay date, and the vertical lines represent standard deviations. Sample sizes were smaller for very early and very late lay dates. The values for the binned lay dates were estimated using a separate glm that contained categorical values for each lay date bin.

Section S3.5 Replacement Clutch Nest Success

The top performing model based on AIC included an interaction between decade and lay date (Table S19). Other models received little support and the model-averaged coefficients are based primarily on the top model (Table S19; Table S20). The 1980s (intercept year) contrasted with other decades in having a positive relationship between nest success of replacement clutches and lay date, as opposed to a negative relationship in other decades

(Table S20; Figure S12). Average rates of nest success were similar for the 1980s, 1990s but the 2000s and 2010s had significantly lower nest success (Figure S12; Table S20)

Table S19 – Results of model selection for nest success of replacement clutches. df is degrees of freedom, AICc is the Akaike Information Criterion and w_i is the Akaike weight.

Model	df	AICc	ΔAICc	w_i
Decade*LayDate	8	1043.36	0	0.97
Decade*LayDate + Decade*LayDate^2	12	1050.09	6.73	0.03
Decade	4	1062.23	18.87	0
LayDate	2	1073.78	30.42	0
LayDate + LayDate^2	3	1075.79	32.43	0
Null	1	1086.17	42.81	0

Table S20 – Model-averaged coefficients, of models listed in Table S19, for nest success of replacement clutches. Models were weighted by Akaike weights. P-values in bold are significant (i.e. P < 0.05).

Parameter	Coefficient	SE	P-value
Intercept	-0.704	0.183	**<0.001**
LayDate	0.320	0.206	0.121
LayDate^2	-0.003	0.041	0.936
1990s	-0.038	0.234	0.871
2000s	-0.665	0.292	**0.023**
2010s	-1.059	0.268	**<0.001**
1990s:LayDate	-0.853	0.257	**0.001**
2000s:LayDate	-0.914	0.325	**0.005**
2010s:LayDate	-0.824	0.311	**0.008**
1990s:LayDate^2	0.004	0.050	0.938
2000s:LayDate^2	-0.002	0.066	0.971
2010s:LayDate^2	-0.004	0.064	0.948

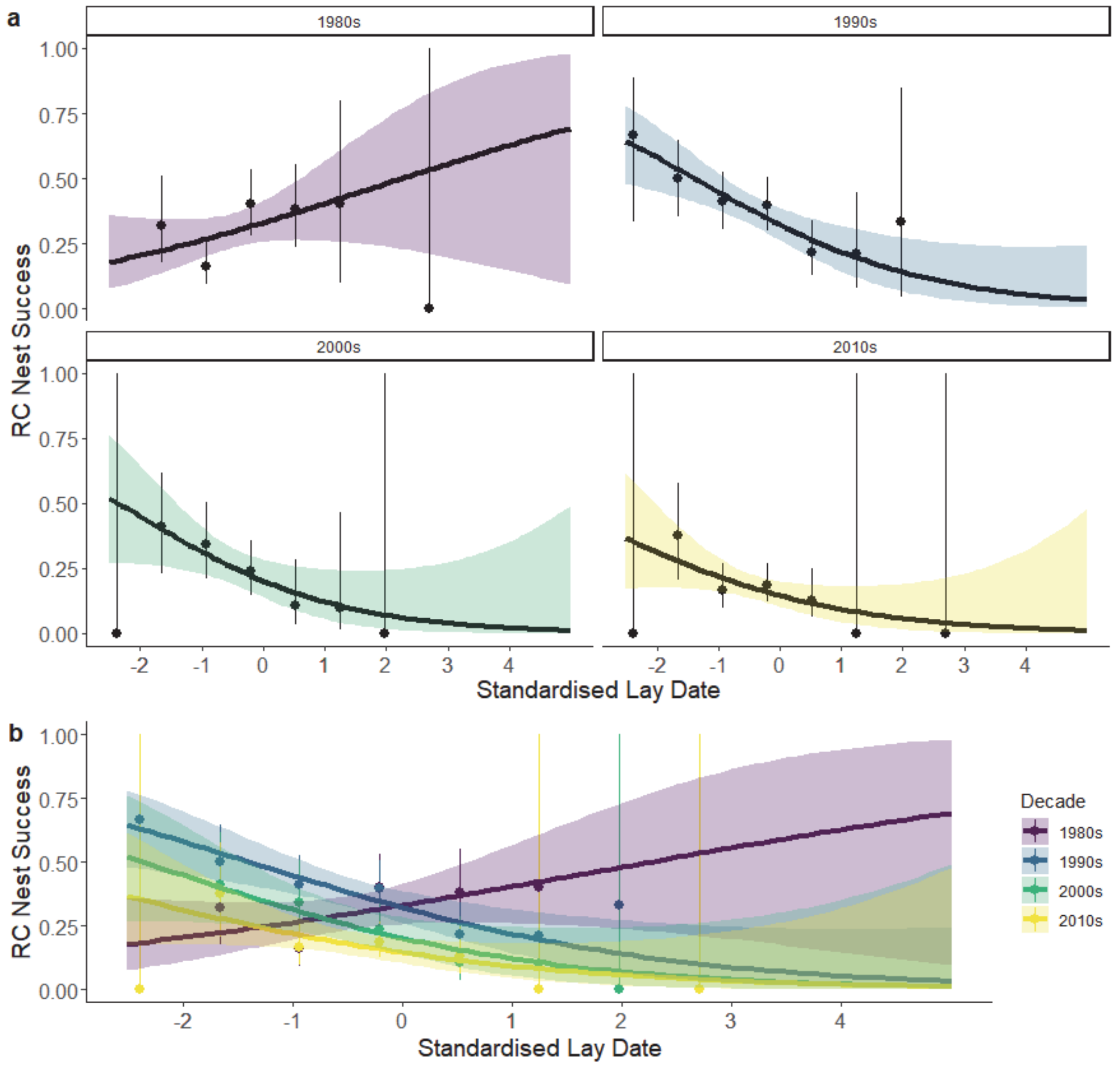

Figure S12 – Predictions and 95% confidence interval (shaded area) of model-averaged coefficients for nest success of replacement clutches (RC) in relation to decade and lay date. a) shows the predictions for the four decades separately and b) overlays these to aid comparisons. The points are the average nest success of replacement clutches across ten bins of lay date, and the vertical lines represent standard deviations. Sample sizes were smaller for very early and very late lay dates. The values for the binned lay dates were estimated using a separate glm that contained categorical values for each lay date bin.

Section S3.6 Number of Hatchlings in a successful Replacement Clutch

No model performed significantly better than a null model meaning that neither decade nor lay date explained much variation in the number of hatchlings of replacement clutches (Table S21). Models containing lay date had similar statistical support and hence carried some weight when averaging model coefficients (Table S21). The number of hatchlings declined

slightly with increasing lay date but all other parameters were near zero (Figure S13; Table S22).

Table S21 – Results of model selection for the number of hatchlings in successful replacement clutches. df is degrees of freedom, AICc is the Akaike Information Criterion and w_i is the Akaike weight.

Model	df	AICc	ΔAICc	w_i
Null	1	648.28	0	0.40
LayDate	2	648.40	0.11	0.38
LayDate + $LayDate^2$	3	650.15	1.87	0.16
Decade	4	651.95	3.66	0.06
Decade*LayDate	8	657.35	9.07	0.00
Decade*LayDate + Decade*$LayDate^2$	12	661.52	13.24	0.00

Table S22 – Model-averaged coefficients, from models listed in Table S21, for the number of hatchlings from successful replacement clutches. Models were weighted by Akaike weights. P-values in bold are significant (i.e. P < 0.05).

Parameter	Coefficient	SE	P-value
Intercept	0.452	0.062	**<0.001**
LayDate	-0.047	0.067	0.481
LayDate^2	0.006	0.028	0.820
1990s	0.007	0.040	0.859
2000s	0.004	0.042	0.923
2010s	-0.010	0.052	0.853
1990s:LayDate	-0.001	0.015	0.965
2000s:LayDate	-0.001	0.024	0.963
2010s:LayDate	0.000	0.020	0.987
1990s:LayDate^2	0.000	0.007	0.985
2000s:LayDate^2	0.000	0.013	0.983
2010s:LayDate^2	0.000	0.015	0.983

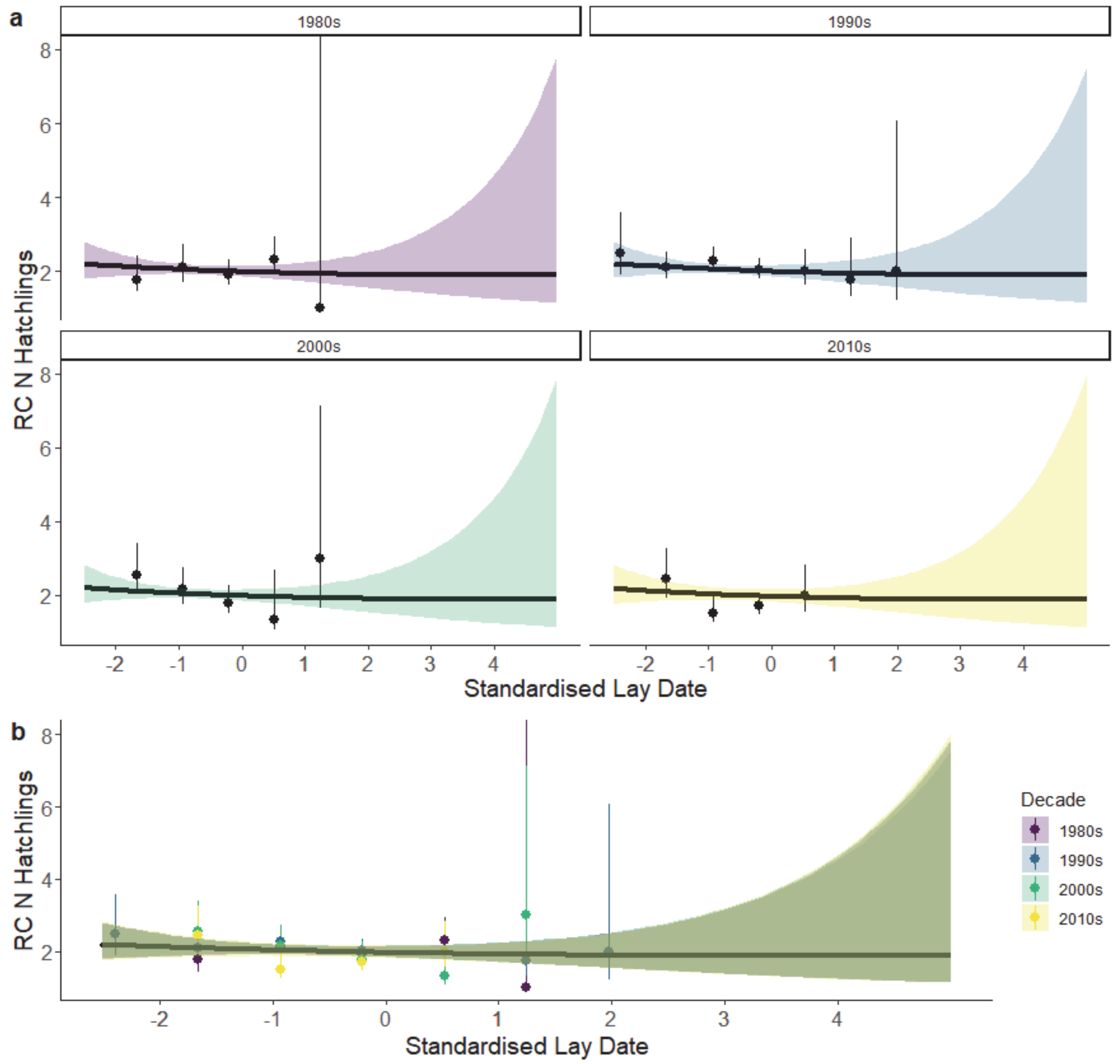

Figure S13 – Predictions and 95% confidence interval (shaded area) of model-averaged coefficients for number of hatchlings of replacement clutches (RC) in relation to lay date. a) separates the predictions across the four decades separately, b) shows the fitted model to all four decades in a single panel. The points are the average number of hatchlings from replacement clutches across ten bins of lay date, and the vertical lines represent standard deviations. Sample sizes were smaller for very early and very late lay dates. The values for the binned lay dates were estimated using a separate glm that contained categorical values for each lay date bin.

Section S3.7 Replacement clutch hatchling survival

Two models performed similarly in explaining hatchling survival which included decade, and an interaction between decade and lay date (Table S23). Given the similar support in the models, we selected the model that included lay date because we wanted to include this state variable in the vital rates that constitute our IPM where statistical support exists. Patterns of hatchling survival in relation to lay date also contrasted among decades, for example no relationship between replacement clutch hatchling survival and lay date in the 1990s, a

positive relationship in the 2000s and negative in the 1980s and 2010s (Figure S14; Table S24). However, the standard errors are large and only the lower hatchling survival of replacement clutches in the 1990s was significant (Figure S14; Table S24)

Table S23 – Results of model selection for hatchling survival of replacement clutches. df is degrees of freedom, AICc is the Akaike Information Criterion and w_i is the Akaike weight.

Model	df	AICc	ΔAICc	w_i
Decade	4	375.32	0	0.60
Decade*LayDate	8	376.78	1.47	0.29
Decade*LayDate + Decade*LayDate^2	12	380.20	4.89	0.05
Null	1	381.10	5.78	0.03
LayDate	2	383.05	7.73	0.01
LayDate + LayDate^2	3	384.02	8.70	0.01

Table S24 – Model-averaged coefficients, from models listed in Table S23, for hatchling survival from replacement clutches. Models were weighted by Akaike weights. P-values in bold are significant (i.e. $P < 0.05$).

Parameter	Coefficient	SE	P-value
Intercept	-1.280	0.260	**<0.001**
LayDate	-0.109	0.251	0.665
LayDate^2	0.010	0.107	0.923
1990s	-0.831	0.393	**0.035**
2000s	-0.055	0.460	0.905
2010s	-0.158	0.424	0.711
1990s:LayDate	0.103	0.295	0.729
2000s:LayDate	0.271	0.526	0.606
2010s:LayDate	-0.213	0.640	0.740
1990s:LayDate^2	-0.011	0.116	0.928
2000s:LayDate^2	-0.051	0.268	0.849
2010s:LayDate^2	-0.070	0.358	0.845

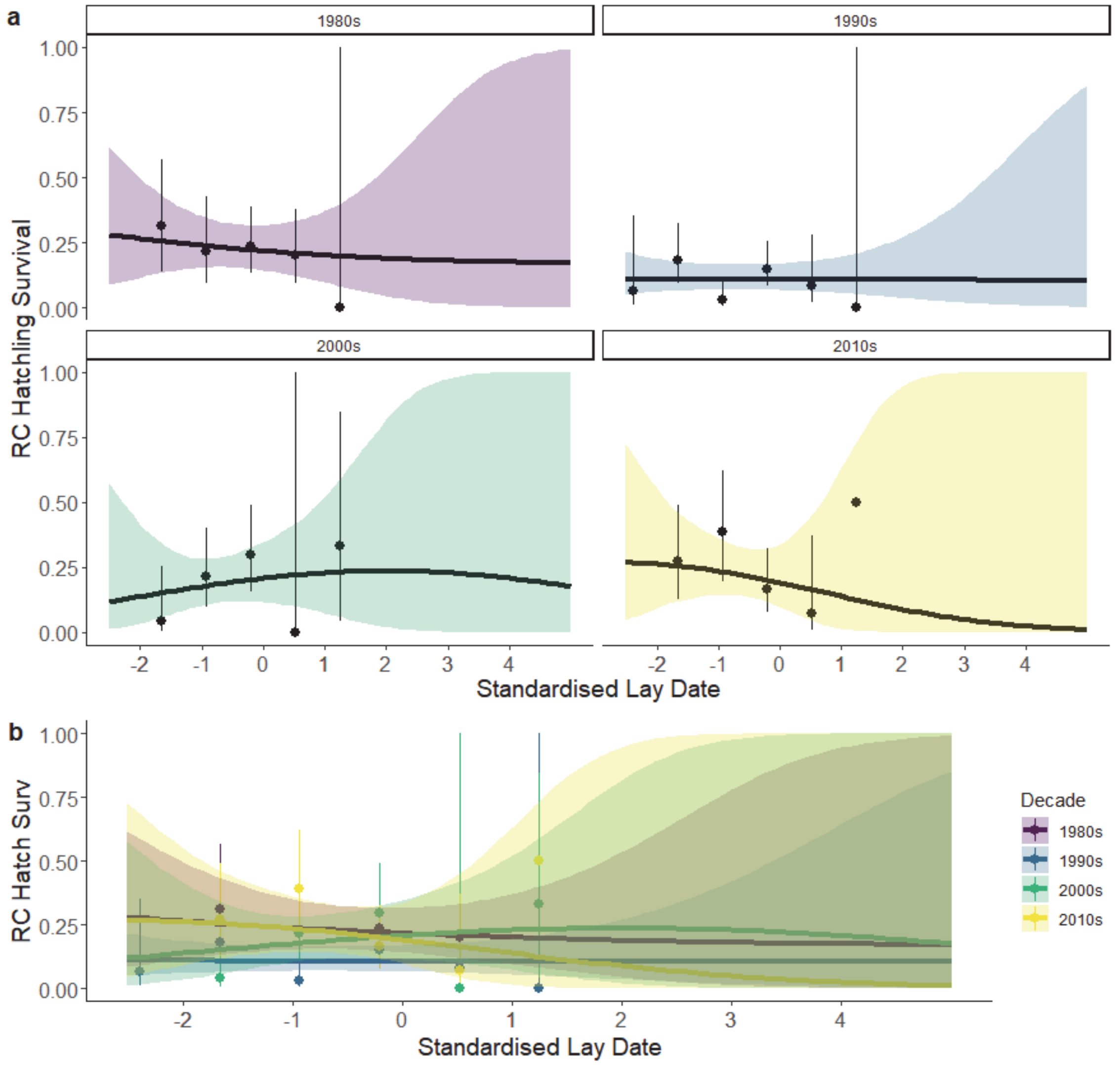

Figure S14 – Predictions and 95% confidence interval (shaded area) of model-averaged coefficients for hatchling survival of replacement clutches (RC) in relation to decade and lay date. a) shows the fitted model for the four decades separately and b) overlays these to aid comparisons. The points are the average hatchling survival of replacement clutches across ten bins of lay date, and the vertical lines represent standard deviations. Sample sizes were smaller for very early and very late lay dates. The values for the binned lay dates were estimated using a separate glm that contained categorical values for each lay date bin.

Section S3.8 Number of recruits

The results of the regression analyses for reproductive vital rates were combined to estimate the number of recruits per individual given its lay date, i.e. the output of the reproduction kernel R(Z) of the IPM shown in Figure 1, which is the number of individuals entering the population at Age 1 given the lay date of the parent. The results illustrate the low reproduction of the 2000s, with a peak of only 0.037 recruits per individual. Meanwhile, the peak number of recruits per individual was similar among the 1980s, 1990s and 2010s (0.137, 0.125, 0133). However, apart from the poor decade of the 2000s, the lay date at which the number of recruits per individual peaks is advancing (Figure S15). This advancement of the relationship between number of recruits and lay date illustrates how the average individual in the population (i.e. standardised lay date = 0; Figure S15) is doing worse. This change in relationship provides additional context for how population growth rates could improve if the population-level average lay dates were to advance (Figure 4 in the main article).

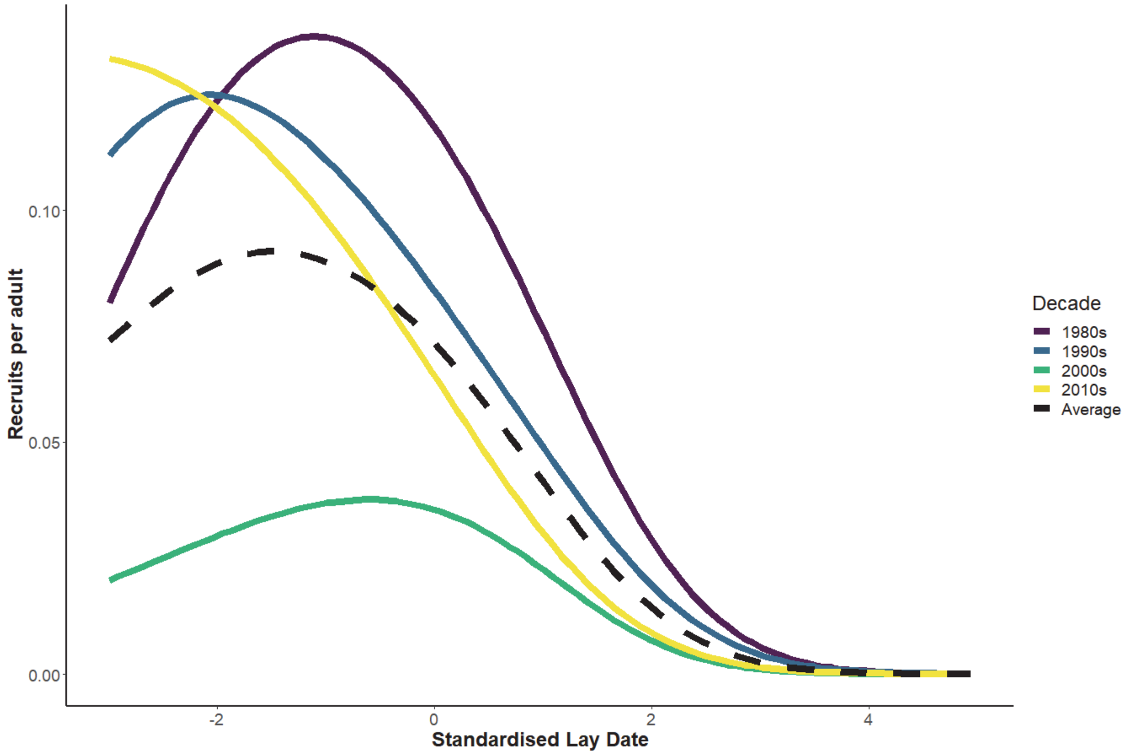

Figure S15 – The number of recruits per adult given the lay date of the adult shown for each decade along with the average for the entire study period (dashed line). The number of recruits per adult is estimated by combining all vital rates associated with the reproductive phase, i.e. it is the output of the reproduction kernel R(Z) shown in Figure 1 of the main text. A recruit is an individual that enters the population at Age 1 (Figure 1 in main text), and is thus not be confused with an individual that is "recruited" to a breeding population.

SECTION S4 REFERENCES

Allen, A. M., B. J. Ens, M. van de Pol, H. van der Jeugd, M. Frauendorf, H. J. van der Kolk, K. Oosterbeek, J. Nienhuis, and E. Jongejans. 2019. Colour-ring wear and loss effects in citizen science mark-resighting studies. Avian Research 10:11.

Barton, K. 2020. MuMIn: Multi-Model Inference. R package version 1.43.17. https://CRAN.R-

project.org/package=MuMIn. R Package version 1.43.17.

Cooch, E. G., and G. C. White. 2019. Adding constraints: MARK and linear models. Pages 6–102 Program MARK: A Gentle Introduction.

Ens, B. J., M. van de Pol, and J. D. Goss-Custard. 2014. The Study of Career Decisions. Oystercatchers as Social Prisoners. In: Advances in the Study of Behavior 46:343–420.

Hothorn, T., K. Hornik, and A. Zeileis. 2006. Unbiased recursive partitioning: A conditional inference framework. Journal of Computational and Graphical Statistics 15:651–674.

Hothorn, T., and A. Zeileis. 2015. partykit : A Toolkit for Recursive Partytioning. Journal of Machine Learning Research 16:3905–3909.

Pinheiro, J., D. Bates, S. DebRoy, and R Core Team. 2020. nlme: Linear and Nonlinear Mixed Effects Models. R Package version 3.1-144.

Van De Pol, M., T. Bakker, D. J. Saaltink, and S. Verhulst. 2006. Rearing conditions determine offspring survival independent of egg quality: A cross-foster experiment with Oystercatchers Haematopus ostralegus. Ibis 148:203–210.

van de Pol, M., K. Oosterbeek, A. L. Rutten, B. J. Ens, J. M. Tinbergen, and S. Verhulst. 2009. Biometric sex discrimination is unreliable when sexual dimorphism varies within and between years: An example in Eurasian Oystercatchers Haematopus ostralegus. Ibis 151:171–180.

Roodbergen, M., B. van der Werf, and H. Hötker. 2012. Revealing the contributions of reproduction and survival to the Europe-wide decline in meadow birds: Review and meta-analysis. Journal of Ornithology 153:53–74.

Tucker, A. M., C. P. McGowan, R. A. Robinson, J. A. Clark, J. E. Lyons, A. Derose-Wilson, R. Du Feu, G. E. Austin, P. W. Atkinson, and N. A. Clark. 2019. Effects of individual misidentification on estimates of survival in long-term mark-resight studies. Condor 121:1–13.
