# Supplementary material for: The demographic causes of population change vary across four decades in a long‐lived shorebird
Source: Ecology. 2022 Mar 3;103(4):e3615. doi: 10.1002/ecy.3615 (PMC9286424; doi:10.1002/ecy.3615)
Supplement: Supplementary file 2 — Appendix S2 [file ECY-103-0-s002.pdf]

The demographic causes of population change vary across four decades in a long-lived shorebird

Andrew M. Allen, Eelke Jongejans, Martijn van de Pol, Bruno J. Ens, Magali Frauendorf, Martijn van de Sluijs, Hans de Kroon

Ecology

**Appendix S2 – IPM construction**

SECTION S1 - SURVIVAL S(Z)

We performed a mark-recapture analysis to investigate survival and whether survival was related to the individual co-variates of age, breeding status and lay. Survival was estimated using a Multi-state model that included both live and dead recoveries (Appendix S1). Our analysis focused on individuals that were ringed on Schiermonnikoog and did not include individuals that had been ringed elsewhere. We only considered age classes and hence whether survival varied among fledglings (first year survival), sub-adult (second and third survival), pre-breeding ages (see Appendix S1) and adults (four year or above; Figure 1). Survival of adults was modelled against the lay date of their breeding attempts, which was estimated as the average lay date during the period of interest. In contrast, sub-adult survival was modelled against the lay date of the nest from which the individual was recruited. In order to know the lay date of the nest, sub-adults had to first survive from fledgling to age one, hence the same individuals were used to estimate fledgling survival (see *Fecundity*) and sub-adult survival (Appendix S1). As described in Appendix S1, the parameters for survival were obtained by model-averaging all considered models. Hence all age classes included a quadratic relationship with lay date and the logistic equation used in the IPM for survival took the form

$$S(Z_{AB}) = \frac{1}{1 + e^{-(\hat{\beta}0 + \hat{\beta}1 Z_L + \hat{\beta}2 Z_L{}^\wedge 2)}}$$

where $Z_A$ are age classes of fledgling, sub-adult (second and third year) and adults, with breeding status B of Pre-Breeder, Breeder or Non-Breeder, which each have their own coefficients whereby $\hat{\beta}0$ is the intercept, $\hat{\beta}1Z_L$ is the beta coefficient for lay date and $\hat{\beta}2Z_L$^2 is the beta coefficients for lay date squared. The coefficients are shown in Appendix S1: Table S3 and S6.

SECTION S2 - GROWTH G(Z',Z)

Growth considers how all three states ($Z_A$, $Z_L$ and $Z_B$) develop from *t* to *t* + 1. Conditional on an individual surviving, an individual first ages ($Z_A$). The lay date ($Z_L$) would then develop for birds that were breeders, whilst non-breeders kept the same lay date. Finally, the breeding probability ($Z_B$) was determined for the next breeding season.

Age has a simple directional model such that

$$G(Z'_A, Z_A) = Z_A + 1$$

where $Z_A$ age increases by one annually until a maximum age of 40 after which all remaining old-age birds die. Note that age in our model is in years from the census moment (May 1), as opposed to general bird ringing terminology that refers to calendar years (CY). So, for example a fledgling is age 0 (as opposed to 1CY) and enters the population at Age 1 (Figure 1) when in bird ringing terminology it is 2CY.

We considered alternative model structures for the growth of lay date including whether lay date was a fixed trait (i.e. constant over time), if lay date at *t* + 1 ($Z'_L$) depended on current lay date ($Z_L$) and if it changed as an individual aged ($Z_A$; Appendix S1). The parameters were determined through model-averaging and consequently included parabolic relationships with both age and lay date (Appendix S1). The growth models were fitted using generalised least squares which allows errors to be correlated or to have unequal variances. We considered different weighting structures to model heteroscedasticity in growth of lay date, with an exponential variance structure providing the best fit. The equation for lay date at time *t* + 1 ($Z'_L$), which only applies to individuals that were a breeder at time *t*, (i.e. $Z_B = 1$), was

$$G(Z'_L, Z_L) = \hat{\beta}0 + \hat{\beta}1 z_L + \hat{\beta}2 z_L{}^{\wedge 2} + \hat{\beta}3 z_A + \hat{\beta}4 z_A{}^{\wedge 2}$$

where $\hat{\beta}0$ is the intercept, $\hat{\beta}1Z_L$ is the slope with lay date at time *t*, $\hat{\beta}1Z_L{}^{\wedge 2}$ is the slope with lay date squared, $\hat{\beta}3Z_A$ is the slope with age and $\hat{\beta}4Z_A{}^{\wedge 2}$ is the slope with lay date squared. Individuals that were non-breeders (i.e. $Z_B = 0$) were multiplied with a diagonal matrix so that the lay date remained the same from *t* to *t* + 1. The coefficients for each parameter are shown in Appendix S1:Table S10. To be able to discretise the continuous state variable of lay date in the IPM, we identified the minimum and maximum lay dates to identify the boundary points, increased these by 10% and subsequently created 100 bins using the midpoint rule.

The final step in growth was to estimate breeding probability for *t* + 1 ($Z_B'$). Prior research indicates that breeding status is not only age dependent, but it also depends on the previous breeding status, whereby breeders are more likely to remain breeders than non-breeders becoming breeders (Ens et al. 1995). The model selection thus considered alternative model structures containing age and previous breeding status (Appendix S1). The model parameters included prior breeding status ($Z_B$), a quadratic relationship with age ($Z_A$) and an interaction between breeding status and age. The logistic equation for probability of breeding at time *t* + 1 ($Z_B'$), with the constraint that $Z_A$ is three or higher, was

$$G(Z'_B, Z_B) = \frac{1}{1 + e^{-(\hat{\beta}0 + \hat{\beta}1 Z_B + \hat{\beta}2 Z_A + \hat{\beta}3 Z_{A}2 + \hat{\beta}4 Z_B Z_A + \hat{\beta}5 Z_B Z_{A}2)}}$$

where $\hat{\beta}0$ is the intercept, $\hat{\beta}1Z_B$ is the current breeding status with 0 = non-breeder and 1 = breeder, $\hat{\beta}2Z_A$ is the slope with a continuous variable of age, $\hat{\beta}3Z_{A2}$ is the slope with age squared, $\hat{\beta}4Z_BZ_A$ is the slope for the interaction between current breeding status and age and $\hat{\beta}5Z_BZ_{A2}$ is the slope for the interaction between current breeding status and age squared. Due to sample size restrictions, it was not possible to estimate separate, age-specific breeding probabilities for non-breeders and pre-breeders. However, since pre-breeders are by default a non-breeding class, the non-breeding breeding probabilities were used for the pre-breeding

state as well. The structure of the IPM was such that it was only possible to move from Pre-Breeder to a Breeder, and once a Breeder, it was possible to alternate between Breeder and Non-Breeder (but impossible to return to Pre-Breeder). The parameter coefficients are shown in Appendix S1:Table S8.

SECTION S3 - FECUNDITY R(Z)

Fecundity, i.e. the number of offspring that were recruited at age one, took the general function of

$$R(Z_L) = \{(NS_1 * NH_1 * HS_1) + (CL_2 * NS_2 * NH_2 * HS_2)\} * FS$$

where NS, NH, HS, CL and FS are vital rates for nest success, number of hatchlings, hatchling survival, probability of initiating a replacement clutch and fledgling survival respectively, for the first (1) and second (2) clutches (Figure 1; Appendix S1). The probability distribution of NH was estimated using a truncated Poisson distribution and took the general form of

$$P(x|x > 0) = \frac{\lambda^x}{x!\,(e^{\lambda} - 1)}$$

where *x* is the number of eggs that hatch (1, 2, …,n), with a zero-truncated Poisson distribution λ. The probability distribution of all other vital rates (NS, HS, CL, FS) were estimated using a binomial distribution and took the general form of

$$P(X = 1) = \frac{1}{1 + e^{-(\hat{\beta}x)}}$$

where P(X) is the probability of success for vital rates NS, HS, CL and FS (Figure 1), and $\hat{\beta}x$ are the beta estimates for the variables *x* included in the vital rate function (Appendix S1).

We only considered whether reproduction vital rates were dependent upon the state variable of lay date ($Z_L$), and not on age ($Z_A$) nor breeding probability ($Z_B$), since only breeders could reproduce ($Z_B = 1$) and breeding probability was dependent upon age ($Z_A$). Multiple regression analyses were performed to determine the relationship of reproductive vital rates with lay date (Appendix S1). We considered three model structures, namely an intercept model, lay date and a quadratic relationship with lay date, and model-averaged the coefficients from all considered models (Appendix S1). Almost all regression analyses for fecundity were performed using a single dataset containing 5,852 nest records (Appendix S1). The parameter coefficients for each of the reproductive stages are shown in Appendix S1: Table S12, S14, S16, S18, S20, S22, S24. The only vital rate for fecundity that was not estimated from this data was fledgling survival to age one. Similar to survival for adults, we performed a multi-state live and dead recoveries mark-recapture analysis to estimate survival to age one for individuals ringed during the fledgling phase (Appendix S1).

SECTION S4. DEVELOPMENT D(Z')

Oystercatchers that were recruited from R(Z,t) were automatically assigned an age ($Z_A = 1$) and a pre-breeding state ($Z_B = 0$). Although oystercatchers only begin breeding at a minimum

age of three, we also assigned newly recruited individuals with a lay date ($Z_L$), but as described in the *Growth* section, this lay date would remain constant until individuals became breeders. We investigated whether the lay date of the parents, from $R(Z_L)$, was associated with the initial lay date of their offspring, with the expectation that parents with earlier than average lay dates may produce offspring with earlier than average lay dates, i.e. D(Z',Z). However, we found no relationship (Appendix S1) and subsequently assigned recruits with a lay date as

$$D(Z') = N(\mu Z_L, \delta Z_L)$$

where lay dates were drawn from a normal distribution with mean ($\mu$) and variance (δ) lay date $Z_L$.

SECTION S5 - REFERENCES

Ens, B. J., F. J. Weissing, and R. H. Drent. 1995. The despotic distribution and deferred maturity: Two sides of the same coin. American Naturalist 146:625–650.
