# Supplementary material for: The demographic causes of population change vary across four decades in a long‐lived shorebird
Source: Ecology. 2022 Mar 3;103(4):e3615. doi: 10.1002/ecy.3615 (PMC9286424; doi:10.1002/ecy.3615)
Supplement: Supplementary file 3 — Appendix S3 [file ECY-103-0-s005.pdf]

The demographic causes of population change vary across four decades in a long-lived shorebird

Andrew M. Allen, Eelke Jongejans, Martijn van de Pol, Bruno J. Ens, Magali Frauendorf, Martijn van de Sluijs, Hans de Kroon

Ecology

**Appendix S3 – Reconstructing the IPM based on other time periods**

SECTION S1 - INTRODUCTION

The main article focuses on a temporal period of decades, which summarises inter-annual changes in vital rates to identify persistent patterns that explain the ongoing decline of the oystercatcher. We also estimated annual population growth rates (λ; Figure S1f; Appendix S4: Figure S1), which required several simplifications to the parameters of the IPM. To construct annual-IPMs, we only estimated the annual intercept of reproduction and survival associated parameters (see Figure 1 and Appendix S1 for details of these parameters). The values of the average IPM (for the study period) were used for the relationship between the vital rates and lay date, along with age-specific breeding probability. Estimating these relationships on an annual basis would not be possible due to data limitations. In addition, certain parameters like age-specific survival of sub-adult classes could not be estimated on an annual basis. To avoid erroneous survival estimates (i.e. zero or 1), in years when survival was not estimated, we made the assumption that survival was the average value for the decade. These decisions emphasise the challenges in building an annual IPM for a species with a complex life cycle, even for a species as well studied as the oystercatcher. The annual population growth rates (Figure S1f) clearly show how stochastic weather events like severe winters (1986, 1995, 2011) can have large effects on λ due to high mortality (Figure S1e). However, there is also clear evidence of a persistent decline, i.e. the majority of years have λ < 1 but that the contribution of vital rates towards this decline vary among years (e.g. Figure S1c-e). Therefore, to identify which vital rates cause the persistent decline of the oystercatcher, we summarised the inter-annual variation and in the main article we chose an unbiased period of decade which is not determined a-priori by patterns in the data.

An alternative approach would be to define the time periods according to patterns in a specific vital rate. We therefore explored and describe the results of an IPM in which the IPM was analysed in three temporal periods which were related to changes in reproduction. A visual inspection of the patterns of nest success shows a decline in nest success from 1998, with levels remaining low thereafter (Figure S1). Although there is no clear recovery in nest success, average levels of nest success along with fledgling production improve from 2008 onwards (Figure S1). We therefore focus on these three periods (Phase 1 = 1983 to 1998, Phase 2 = 1998 to 2008, Phase 3 = 2008 to 2019) in this appendix.

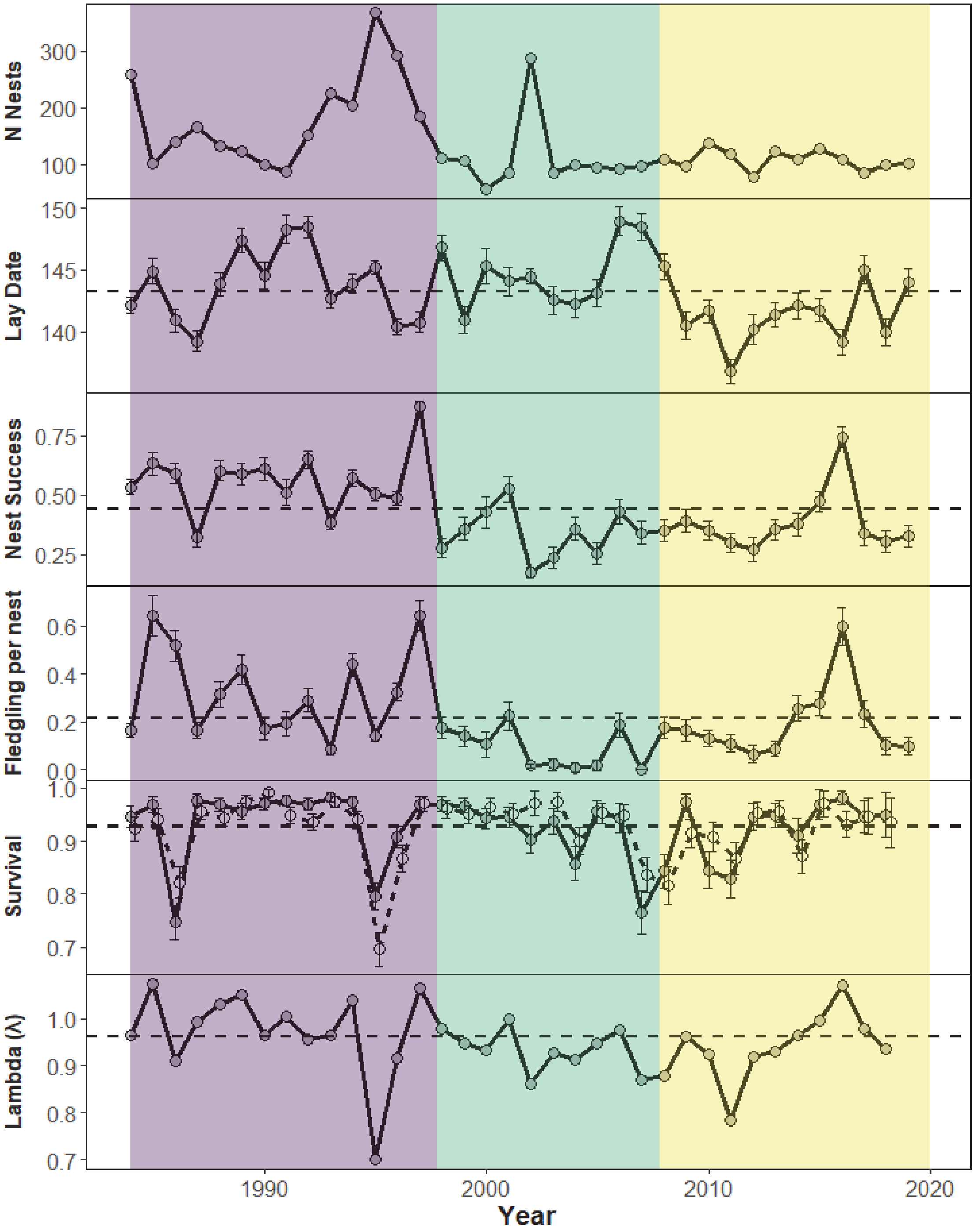


**Figure S1** Visualisation of data for a) number of nests, b) average lay date, c) nest success, d) fledglings per nest, e) sex-specific survival of adult breeders and f) population growth rates (λ). The shaded regions show the three study periods according to changes in nest success with Phase 1 (purple, left shaded area) having higher levels of nest success, Phase 2 (green, middle shaded area) having low nest success and Phase 3 (yellow, right shaded area) having generally higher nest success and fledglings per nest than Phase 2. The error bars are the standard error and in e) the grey points with solid lines are females and hollow points with

dashed line are males. The horizontal dashed line in each panel is the average through the entire study period.

We replicated the analyses from the main article in that we used regression analyses to identify the relationships between reproductive vital rates and lay date, and whether these varied amongst the three periods. Regression analyses were also used to estimate age-specific breeding probabilities in each period. Multi-state live and dead recovery analyses were used to investigate adult survival and how this varied with breeding status, lay date and the three periods. Multi-state live and dead recovery analyses were also used to obtain age-specific estimates of pre-breeder survival and how these varied among the three temporal periods and the lay dates of nests from which chicks hatched. Similar to the main article, a single growth function for lay date was used, i.e. period-specific growth functions of lay date were not incorporated.

## SECTION S2 - RESULTS

The projected average population growth rate (λ) during the whole study period was 0.961, compared with the λ based on decadal periods of 0.962. The lambda in each of the three periods were 0.968, 0.942 and 0.963 for Phase 1, 2 and 3, respectively (Figure S2).

Nest success explained most variation in reproduction (Figure S2a, d) and an important contrast with the main article is that no period-specific variation was detected in adult survival of breeders and non-breeders (Figure S2b, e). A survival model containing an interaction between breeding state and the temporal period had a ΔAICc of 59.05 compared to a model with breeding state alone. The average survival rates used in the IPM for breeders was 0.928 and non-breeders was 0.887. Similar to the main article, the most recent period has experienced higher breeding probabilities which is buffering the decline (Figure S2c, f).

## SECTION S3 - DISCUSSION

The two approaches (decadal periods in the main text; phases based on patterns in nest success in this appendix) yielded similar results insofar as the average population growth rates were near identical in this approach and that of the main article. This result provides additional confirmation about the projected decline of the species, especially when considering that the relationships between vital rates and temporal periods sometimes varied between the decadal approach and the demographic phases approach.

Changes in nest success explained most variation in lambda, with almost no contribution from hatchling survival. Furthermore, there was no temporal trend in adult survival, for which variation amongst decades was found but not within the demographic phases identified from nest success. These results outline the potential risk in defining temporal periods by a single vital rate: the very changes in the selected rate are likely to be amplified in the results of the IPM and may subsequently mask the contributions of other vital rates.

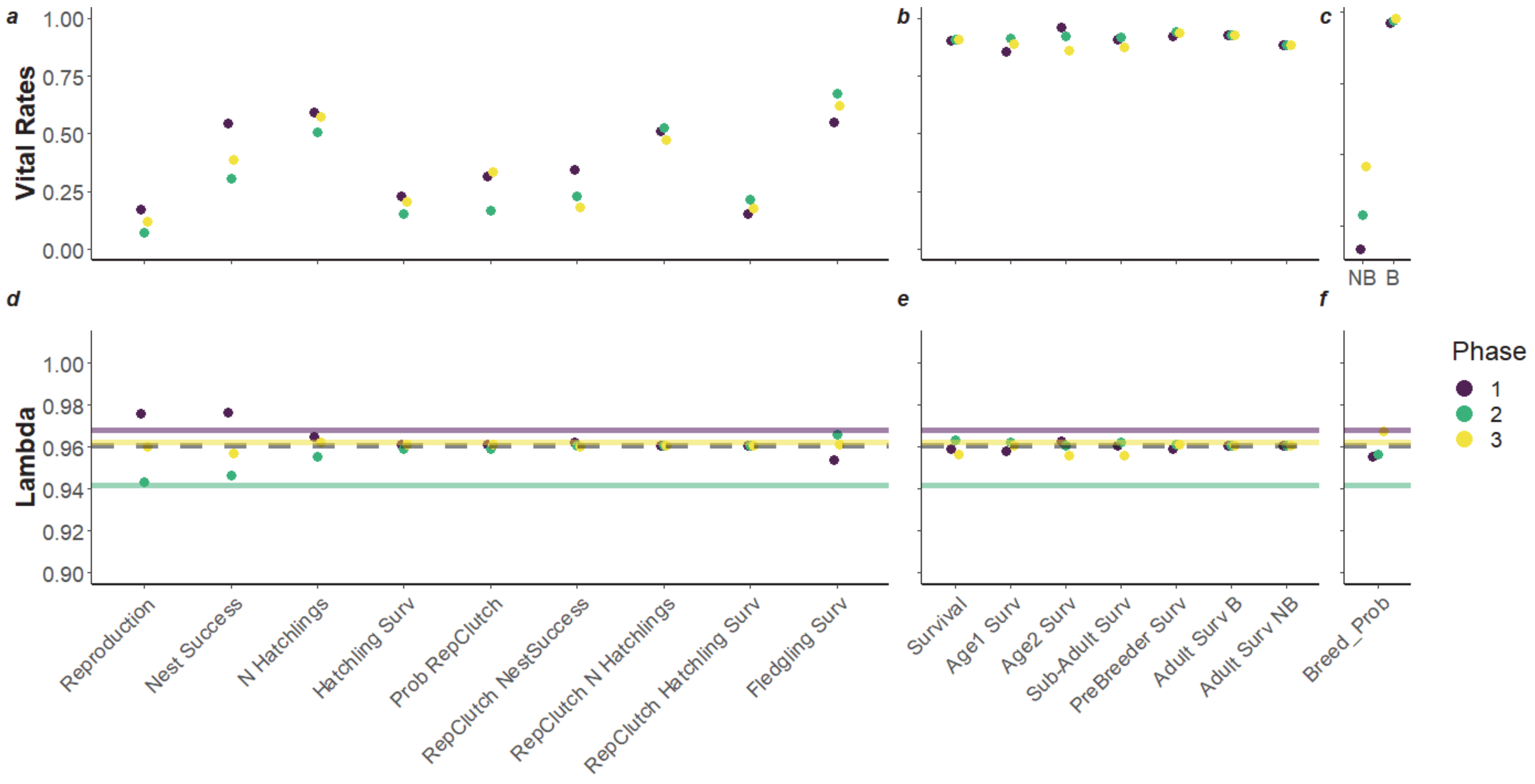


**Figure S2** - Vital rates (a, b, c) and population growth rates (lambda, λ) (d, e, f) in three demographic phases (Figure S1) for reproduction (a, d), survival (b, e) and breeding probability (c, f). Number of hatchlings (a) was divided by 4 (maximum clutch size) for visualisation. Breeding probability is shown for non-breeders (NB) and breeders (B) in (c) but combined in (f). Population growth rates (d, e, f): The coloured lines depict λ from the IPMs for each phase, and the dashed line is the average λ. The coloured points should be compared to the dashed line and depict the change in λ when a vital rate is changed from the study average to the phase-specific value. Coloured points further from the dashed line indicate a greater relative contribution. “Reproduction” (a,d) and “Survival” (b,e) combines all parameters (i.e. to right in the panel).

Temporal periods defined by nest success also lack a temporal period in which the population was stable. The results of Figure 2 in the main article show that nest success in the 1990s was near identical to the 1980s. However, the population had already begun declining in the 1990s due to lower reproduction in the 1990s, largely due to lower hatchling and fledgling survival, along with lower survival of sub-adult and pre-breeder age classes. These dynamics of the 1990s are partly evident in Figure S2, where the first phase has lower fledgling survival, second year (Age1) survival, and pre-breeder survival, than later phases. The lack of a stable period (e.g. the 1980s) means that follow-on simulations that explore how stable population growth rates can be achieved becomes more challenging.

The approach we follow in this appendix can therefore be helpful to explore the contribution of specific vital rates during pre-defined periods of interest. The pre-defined periods can be defined by temporal changes in a vital rate, or as we show here, they may need to be defined by changes in several vital rates. Exploring these options thus helps identify how the chosen time-window may influence the results of the IPM. Another approach in defining temporal periods may be towards specific conservation or management goals – for example, how do the last three or five years compare to the average of the past forty years (in the case of the oystercatcher). These approaches tend to lean towards a specific research or management question however, and the results here demonstrate that using a "random" temporal period, i.e. one that is not defined by a vital rate, remains a viable alternative for detecting demographic mechanisms of population decline without being influenced by *a priori* choices.
