# Supplementary material for: The demographic causes of population change vary across four decades in a long‐lived shorebird
Source: Ecology. 2022 Mar 3;103(4):e3615. doi: 10.1002/ecy.3615 (PMC9286424; doi:10.1002/ecy.3615)
Supplement: Supplementary file 4 — Appendix S4 [file ECY-103-0-s004.pdf]

The demographic causes of population change vary across four decades in a long-lived shorebird

Andrew M. Allen, Eelke Jongejans, Martijn van de Pol, Bruno J. Ens, Magali Frauendorf, Martijn van de Sluijs, Hans de Kroon

Ecology

Appendix S4 - Supplementary Tables and Figures

Table S1 – Results of a Tukey HSD test comparing mean lay dates across the four decades of the study.

Comparison	Difference	Lower	Upper	P adj.
1990s-1980s	1.67	0.63	2.71	<0.001
2000s-1980s	2.36	1.21	3.52	<0.001
2010s-1980s	-1.22	-2.35	-0.08	0.029
2000s-1990s	0.69	-0.31	1.69	0.281
2010s-1990s	-2.89	-3.86	-1.92	<0.001
2010s-2000s	-3.58	-4.68	-2.49	<0.001

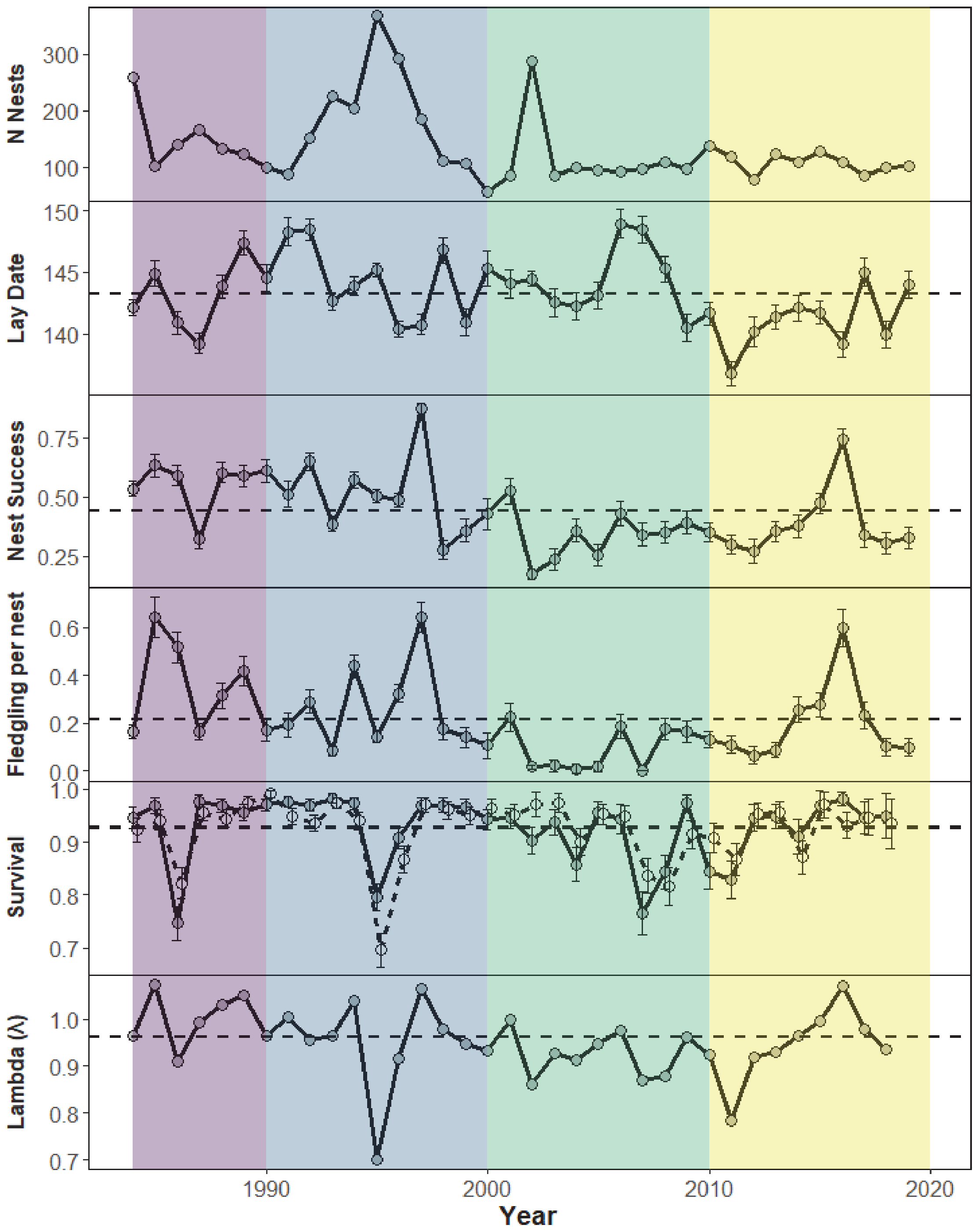

Figure S1 – Inter-annual population dynamics for the Eurasian oystercatcher on Schiermonnikoog between the years 1984 and 2019. a) is the number of first clutches monitored, b) the average lay date, c) average nest success, d) the average number of fledglings per nest, e) sex-specific survival of breeding adults and f) population growth rates (λ). Panel e) shows sex-specific survival with females shown as solid lines with grey filled points and males shown with dashed lines and a hollow point. The points are jittered slightly in e) to avoid overlapping error bars. The horizontal dashed lines are the average for the

entire period, the shaded regions demarcate the different decades (years on x-axis) and error bars are the standard errors.

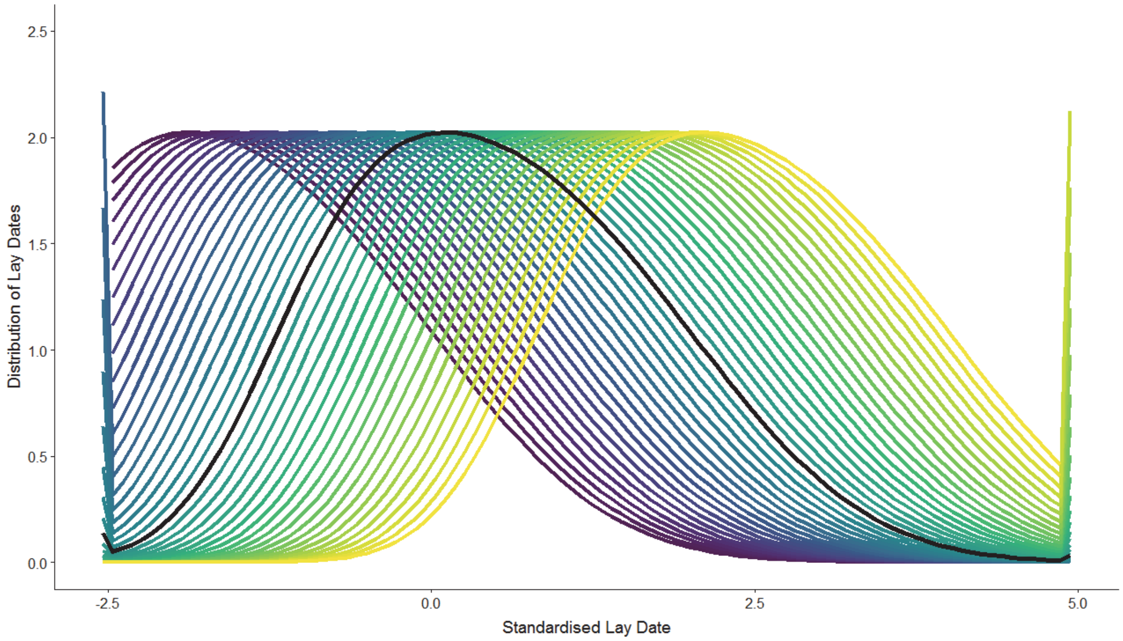

Figure S2 – Lay date distributions within the study system shown in black and the remaining coloured distributions show 40 different lay date distributions with the mean increasing incrementally by one day until a max 20 days earlier and 20 day later than the study mean.

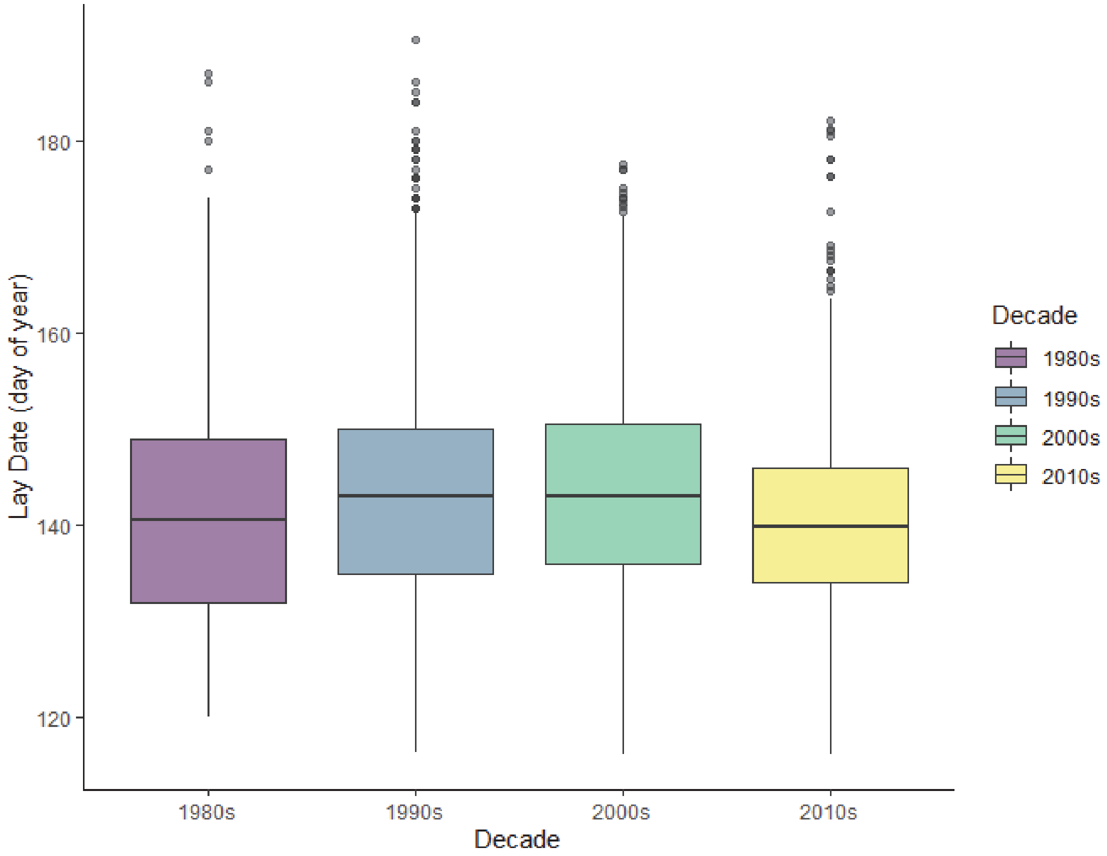

Figure S3 - Median lay date during the four decades of the study period. The boxes show the interquartile range and the lines include 95% of observations and points lie outside the 95% range.
