# Supplementary material for: The demographic causes of population change vary across four decades in a long‐lived shorebird
Source: Ecology. 2022 Mar 3;103(4):e3615. doi: 10.1002/ecy.3615 (PMC9286424; doi:10.1002/ecy.3615)
Supplement: Supplementary file 5 — Appendix S5 [file ECY-103-0-s006.pdf]

The demographic causes of population change vary across four decades in a long-lived shorebird

Andrew M. Allen, Eelke Jongejans, Martijn van de Pol, Bruno J. Ens, Magali Frauendorf, Martijn van de Sluijs, Hans de Kroon

Ecology

Appendix S5 – Comparing population growth rates with number of breeding pairs

SECTION S1 - INTRODUCTION

Our analysis focuses on the population growth rates of an island population of Eurasian Oystercatchers. Due to processes like immigration, emigration and settlement rates, the population growth rate may not necessarily reflect the status of the breeding population. This may be especially true for a long-lived species which may occupy territories for several years and hence turnover of individuals may be low. The growth rates we estimate align with those of earlier studies, i.e. that the population was initially stable before a sharp decline ensued of nearly 9% (Oosterbeek et al. 2006), whilst van de Pol (2006) estimated an average rate of decline 5% compared to the value in our study of 3.8% which covers a longer time period. We further validated our results by comparing the population growth rates from the integral project model (IPM) to the number of breeding pairs in our study area. The number of breeding territories was only estimated in those areas that were monitored during the period of decline, i.e. from the mid-1990s until present day. We estimated the three-year average number of breeding territories for the period 2000 (1999, 2000, 2001), 2010 (2009, 2010, 2011) and 2019 (2017, 2018, 2019), and compared the percentage change between each period (Table S1).

SECTION S2 - RESULTS

The rate of decline in breeding numbers appears to be less variable than the population growth rates estimated in the IPM (Figure 2; Table S1). Whilst the IPM estimated a period of stark decline (2000s; Figure2) followed by a more modest decline (2010s; Figure 2), the rates of decline have been more constant in the number of breeding territories (Table S1). The results of the IPM are supported in that the oystercatcher population is experiencing a persistent decline, which averages 3.8% in the IPM whilst the rates of decline for the number of breeding territories varies amongst study areas and per decade (average for 2000s = -2.9% and 2010s = -2.7%). The difference may be due to the high survival and fidelity of the oystercatcher, meaning that territories could be occupied for a number of years. In addition, the IPM shows how reproduction parameters were primarily responsible for the change in population growth rates, and hence lower rates of recruitment may only become evident in later years. The comparison of the IPM results to that of previous studies, together with the status of breeding territories, and the similarity in overall rates of decline is thus reassuring in validating the estimated population growth rates.

Table S1 – Three year averages for the number of breeding territories in 2000, 2010 and 2019 and the change (%) in the number of breeding territories from 2000 to 2010 (2000s) and from 2010 to 2019 (2010s) for six study areas which were monitored during the study period.

Area	2000	2010	2019	2000s	2010s
1	20.0	14.3	11.0	-2.8%	-2.3%
2	17.0	10.7	5.3	-3.7%	-5.0%
3	19.0	9.3	6.7	-5.1%	-2.9%
4	22.7	20.3	21.7	-1.0%	0.6%
5	19.0	16.0	9.3	-1.6%	-4.2%
6	11.3	7.7	6.0	-3.2%	-2.2%

SECTION S3 - REFERENCES

Oosterbeek KH, van de Pol M, de Jong ML, Smit CJ, Ens BJ (2006) Scholekster populatie studies; bijdrage aan de zoektocht naar de oorzaken van de sterke achteruitgang van de Scholekster in het Waddengebied. Alterra-Rapport 1344, SOVON-onderzoeksrapport 2006/2005 Alterra, Wageningen. Available at: https://library.wur.nl/WebQuery/wurpubs/fulltext/21248

van de Pol, M. 2006. State-dependent life-history strategies: a long-term study on Oystercatchers. Thesis. University of Groningen, Groningen, The Netherlands. Available at: https://research.rug.nl/en/publications/state-dependent-life-history-strategies-a-long-term-study-on-oyst
