# Supplementary material for: The demographic causes of population change vary across four decades in a long‐lived shorebird
Source: Ecology. 2022 Mar 3;103(4):e3615. doi: 10.1002/ecy.3615 (PMC9286424; doi:10.1002/ecy.3615)
Supplement: Supplementary file 6 — Appendix S6 [file ECY-103-0-s003.pdf]

The demographic causes of population change vary across four decades in a long-lived shorebird

Andrew M. Allen, Eelke Jongejans, Martijn van de Pol, Bruno J. Ens, Magali Frauendorf, Martijn van de Sluijs, Hans de Kroon

Ecology

Appendix S6 - Sex-specific Integral projection models

SECTION S1 - INTRODUCTION

As described in the main text, sex differences can only be analysed for adult survival (breeders and non-breeders) and breeding probability. In the case of survival, a model including sex had a worse AICc than a model excluding sex (ΔAICc = 1.17), but would nonetheless contribute weighting to the model-averaged parameters should it be included (weightings were 0.64 excluding sex versus 0.36 including sex; Appendix S1: Table S4). The model-averaged parameter coefficients for sex-specific adult survival are shown in Table S1. In the case of breeding probability, a model including sex had a lower AICc than a model excluding sex (ΔAICc = 1.28), and the model-averaged sex-specific parameters for breeding probability are shown in Table S2. As described in the main article, and in Appendix S1, *inter alia* given the similar performance of models and that most variation in survival was due to non-breeders which had minimal influence on population growth rates, the main article did not distinguish between males or females. However, to further explore the influence that differences in sex-specific adult survival (Table S1) and breeding probability (Table S2) have for population growth rates, we built sex-specific integral projection models (IPMs), that is an IPM for males and an IPM for females. Reproduction, lay date and fledgling/sub-adult/pre-breeder survival were the same for both the male and female IPMs. We did not include sex as an extra state variable in the model, as this would require marriage functions within the IPM (e.g. Stubberud et al. 2019) and we do not have sufficient knowledge to parameterise such an IPM, importantly how vital rates during the reproduction phase, fledgling survival and dispersal may influence the sex distribution of the population. Therefore, like the main text, here we estimated sex-specific average and decade-level population growth rates and how changes in each of the vital rates explained changes in population growth rates among decades.

Table S1 - Model-averaged beta coefficients for models of adult survival that also included sex. Parameters were weighted according to the Akaike weights shown in Appendix S1:Table S4. The intercept is adult survival of female breeders in the 1980s, NB is non-breeders, SexM is males, LD is lay date, se is the standard error, lcl and ucl are the 95% lower and upper confidence limits.

Parameter	Coefficient	se	lcl	ucl
Intercept	2.706	0.104	2.502	2.910
SexM	-0.024	0.111	-0.242	0.194

NB	-0.349	0.341	-1.017	0.319
SexM:NB	-0.132	0.370	-0.858	0.594
1990s	0.047	0.192	-0.329	0.422
2000s	-0.281	0.138	-0.552	-0.010
2010s	-0.306	0.161	-0.621	0.010
NB:1990s	-0.393	0.406	-1.189	0.404
NB:2000s	0.286	0.361	-0.421	0.994
NB:2010s	-0.536	0.387	-1.294	0.223
SexM:1990s	-0.140	0.230	-0.591	0.310
SexM:2000s	0.075	0.172	-0.262	0.411
SexM:2010s	0.090	0.197	-0.296	0.476
SexM:NB:1990s	0.194	0.448	-0.683	1.071
SexM:NB:2000s	0.012	0.366	-0.705	0.729
SexM:NB:2010s	0.052	0.392	-0.716	0.820
LD	0.000	0.000	0.000	0.000
LD^2	0.000	0.000	0.000	0.000

Table S2 - Model-averaged coefficients, of models shown in Appendix S1: Table S7, for next-year's breeding probability. SexM is males, SE is stand error and P-values shown in bold are significant (i.e. $P<0.05$).

Parameter	Estimate	SE	P-value
(Intercept)	-1.131	1.033	0.274
SexM	1.188	0.984	0.227
NonBreeder	-3.551	1.222	**0.004**
Pre2000s	0.378	0.685	0.581
2010s	1.522	0.757	**0.044**
Age	0.626	0.110	**<0.001**
Age^2	-0.019	0.003	**<0.001**
NonBreeder:SexM	-1.313	1.149	0.253
Pre2000s:SexM	-0.526	0.883	0.551
2010s:SexM	-0.738	0.909	0.417
Pre2000s:NonBreeder	-0.950	0.873	0.277
2010s:NonBreeder	0.405	0.984	0.681
Age:NonBreeder	0.266	0.156	0.088
Age^2:NonBreeder	-0.014	0.006	**0.013**
Pre2000s:NonBreeder:SexM	1.024	1.198	0.393
2010s:NonBreeder:SexM	0.476	1.145	0.678
Age:Pre2000s	-0.001	0.039	0.978
Age:2010s	0.000	0.029	0.990
Pre2000s:Age^2	0.000	0.002	0.999
2010s:Age^2	0.000	0.001	0.981
Age:SexM	0.000	0.013	0.985

Age^2:SexM	0.000	0.000	0.980
Age:Pre2000s:SexM	0.000	0.023	0.985
Age:2010s:SexM	0.000	0.018	0.986
Pre2000s:Age^2:SexM	0.000	0.001	0.990
2010s:Age^2:SexM	0.000	0.000	0.995
Age:NonBreeder:SexM	0.000	0.015	0.987
Age^2:NonBreeder:SexM	0.000	0.001	0.987

SECTION S2 - RESULTS

Population growth rates were similar among the female, male and average IPMs (Table S3), which may be expected for a long-lived monogamous species (Heg and Treuren 1998) with near 50:50 sex ratio of hatchlings (Heg et al. 2000). In general, the decline of the females was worse than that of males, with the largest differences in the two most recent decades (0.008 in 2000s and 0.004 in 2010s). This is largely attributable to the lower survival in the last two decades (Figure S1) and in the 2000s, females also had a lower breeding probability but this difference disappears in the 2010s (Figure S1). The demographic drivers of changes in population growth rates remained similar in both the female and male IPMs (Figure S2; Figure S3). The slightly lower survival of females in the last two decades contribute to lower growth rates compared with males, but this is partially offset by higher breeding probabilities in the last two decades (Figure S2; Figure S3). These rather small effects of sex do not affect the conclusions of our main analyses.

Table S3: Population growth rates for males, females and the average shown in the main text. The average column is not the average of the male and female IPMs, but is the result of the main text, which uses all data including individuals of unknown sex. Population growth rates are shown for each decade, along with the average (Avg) for the entire study period.

Decade	Males	Females	Average
1980s	1.005	1.001	1.003
1990s	0.962	0.961	0.963
2000s	0.915	0.907	0.911
2010s	0.959	0.955	0.956
Avg	0.963	0.961	0.962

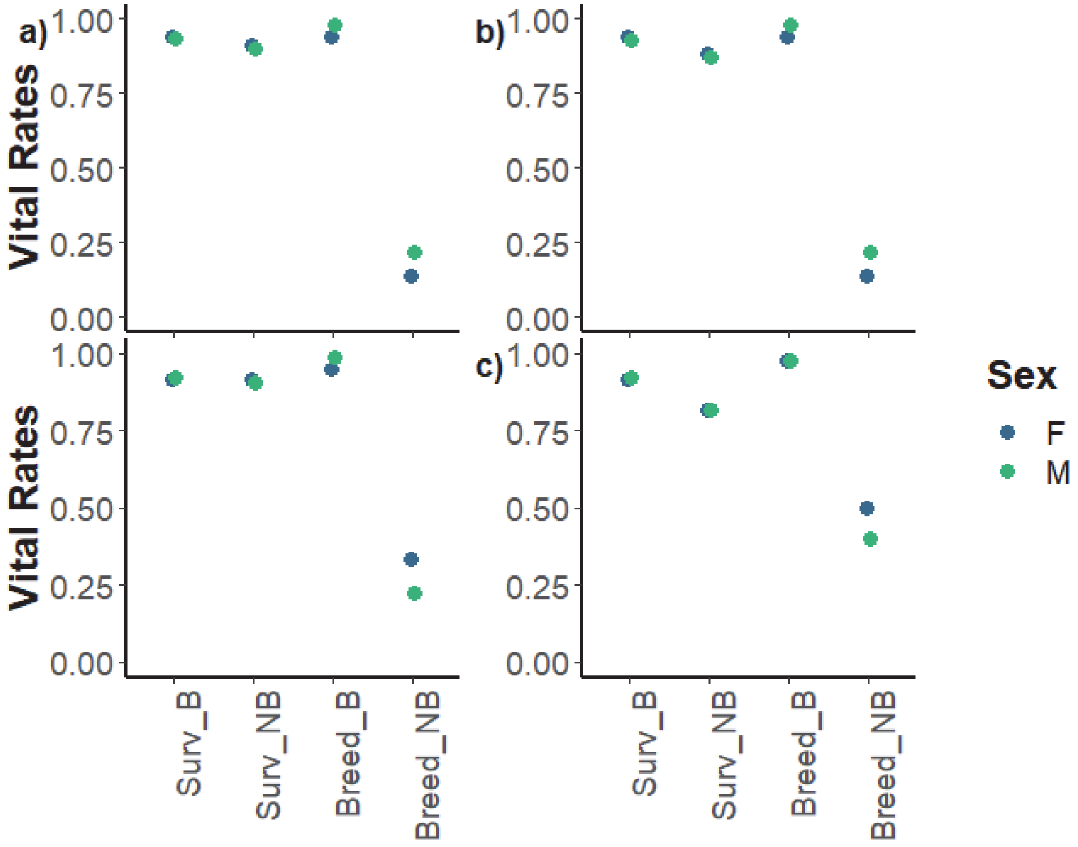

Figure S1: Comparison of male and female vital rates for a) 1980s, b) 1990s, c) 2000s and d) 2010s. The displayed results are from a simplified model structure that only includes sex, decade and breeding status (i.e. no lay date effects on survival nor age effects on breeding probability).

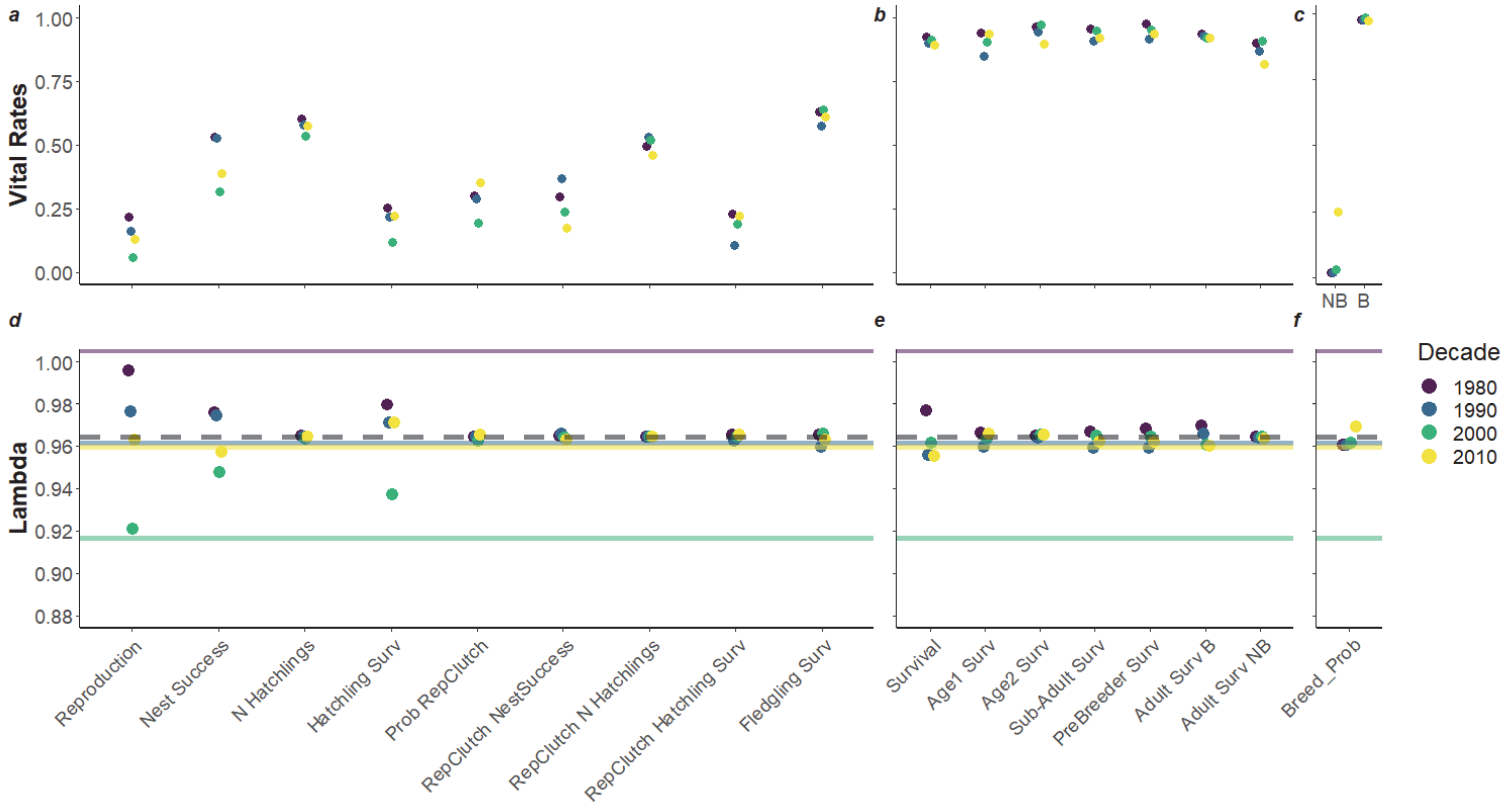

Figure S2: Vital rates (a, b, c) and population growth rates (lambda) (d, e, f) for males for each decade split between reproduction (a, d), survival (b, e) and breeding probability (e, f). Almost all vital rates (a, b, c) were bounded between 0 and 1, except number of hatchlings which was normalised for visualisation purposes by dividing by 4 (in general the maximum clutch size). Vital rates of breeding probability (c) are shown for non-breeders (NB) and breeders (B) but combined in the population growth rate simulations (f). Population growth rates (d, e, f): The coloured solid lines depict lambda from the IPMs for each decade, whilst the dashed line is the lambda from the average IPM for the entire study period. The coloured points should be compared to the dashed line (average IPM) and depict the change in lambda when a vital rate is changed from the average values of the study period, to the decade-specific parameter instead. Coloured points that are further from the dashed line indicate a greater relative contribution with either a higher/lower lambda (above/below the dashed line). The axis labels of "Reproduction" (a,d) and "Survival" (b,e) are the overall estimates, i.e. they combine all appropriate parameters that follow in the panel.

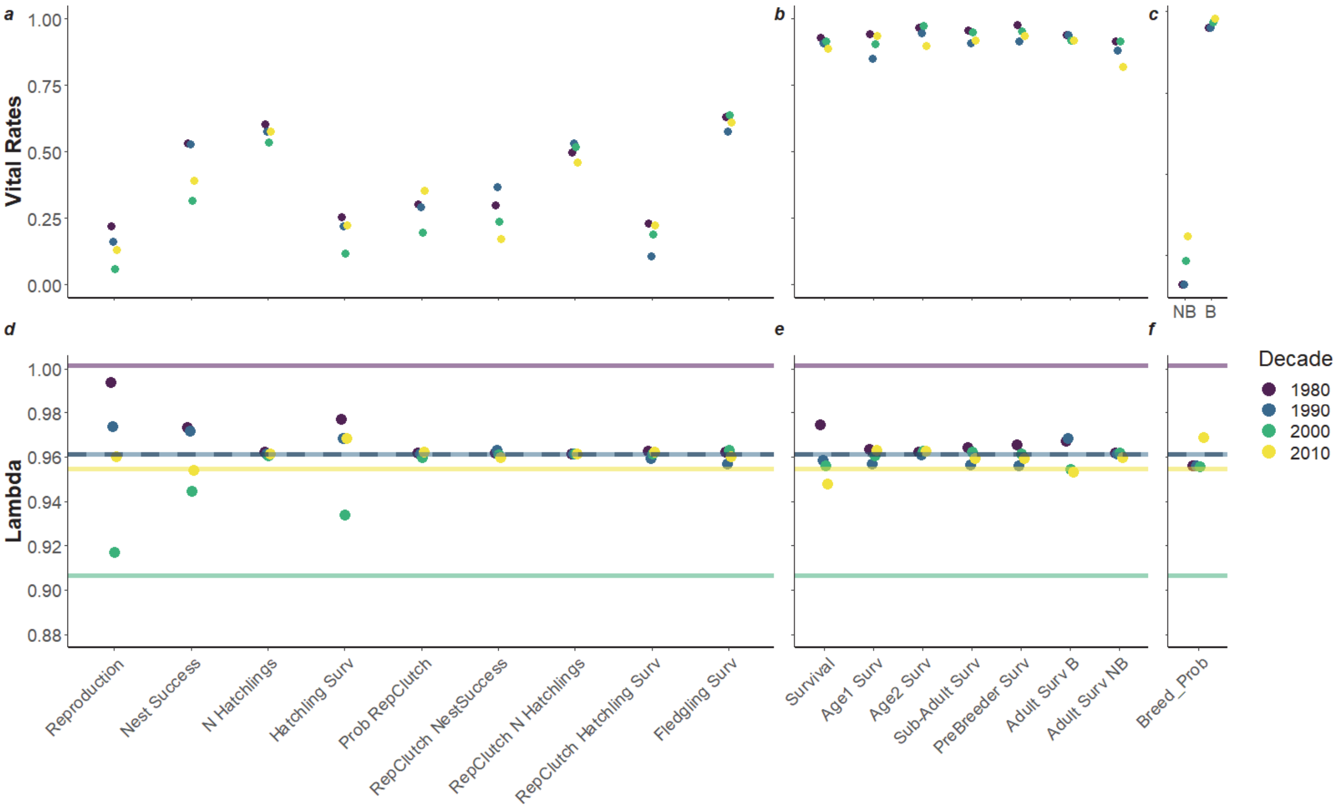

Figure S3: Vital rates (a, b, c) and population growth rates (lambda) (d, e, f) for females for each decade split between reproduction (a, d), survival (b, e) and breeding probability (e, f). Almost all vital rates (a, b, c) were bounded between 0 and 1, except number of hatchlings which was normalised for visualisation purposes by dividing by 4 (in general the maximum clutch size). Vital rates of breeding probability (c) are shown for non-breeders (NB) and breeders (B) but combined in the population growth rate simulations (f). Population growth rates (d, e, f): The coloured solid lines depict lambda from the IPMs for each decade, whilst the dashed line is the lambda from the average IPM for the entire study period. The coloured points should be compared to the dashed line (average IPM) and depict the change in lambda when a vital rate is changed from the average values of the study period, to the decade-specific parameter instead. Coloured points that are further from the dashed line indicate a greater relative contribution with either a higher/lower lambda (above/below the dashed line). The axis labels of “Reproduction” (a,d) and “Survival” (b,e) are the overall estimates, i.e. they combine all appropriate parameters that follow in the panel.

SECTION S3 - REFERENCES

Heg, D., N. J. Dingemanse, C. M. Lessells, and A. C. Mateman. 2000. Parental correlates of offspring sex ratio in Eurasian Oystercatchers. Auk 117:980–986.

Heg, D., and R. van Treuren. 1998. Female–female cooperation in polygynous oystercatchers. Nature 391:687–691.

Stubberud, M. W., Y. Vindenes, L. A. Vøllestad, I. J. Winfield, N. C. Stenseth, and Ø. Langangen. 2019. Effects of size- and sex-selective harvesting: An integral projection model approach. Ecology and Evolution 9:12556–12570.
